# Sequential Soybean Phenotypes for Grain Yield Prediction in Reciprocal Two-Season Validation

**DOI:** 10.3390/plants15182865

**Published:** 2026-09-19

**Authors:** Renan Falcioni, José Salvador Simoneti Foloni, Luis Guilherme Teixeira Crusiol, Marcos Rafael Nanni, José Renato Bouças Farias

**Affiliations:** 1 National Soybean Research Center, Brazilian Agricultural Research Corporation, Rodovia Carlos João Strass, s/nº, Acesso Orlando Amaral, Distrito de Warta, Londrina 86085-981, PR, Brazil; luis.crusiol@colaborador.embrapa.br (L.G.T.C.); joserenato.farias@embrapa.br (J.R.B.F.); 2 Graduate Program in Agronomy, State University of Maringá, Av. Colombo 5790, Maringá 87020-900, PR, Brazil; mrnanni@uem.br

**Keywords:** calibration, covariate shift, elastic net, field phenotyping, soybean, temporal validation, yield prediction

## Abstract

Whether sequential crop phenotypes improve grain yield prediction after transfer to another growing season remains poorly quantified. We evaluated soybean (*Glycine max* (L.) Merr.) at one experimental site over two seasons in a split-plot experiment with five sowing periods, two cultivars, four seeding rates, and four blocks (160 subplots per season). Information gates were cumulative predictor sets available through V4, R2, R5, and R6. They combined subplot means of leaf area, total dry matter, branching, main-stem nodes, and plant height from distinct destructive samples. Grain yield at R8 was the outcome. Elastic-net models estimated phenotypic corrections to development-season means for matching management combinations. Reciprocal whole-season evaluation used development-only grouped tuning and conditional within-season whole-block bootstrap intervals. The management comparator produced root mean squared errors (RMSEs) of 1398 and 1394 kg ha^−1^ and predictive R^2^ values of 0.282 and −0.182 for 2021/22 → 2022/23 and the reciprocal direction, respectively. Paired RMSE gains from cumulative phenotypes ranged from −0.419% to 0.038% and from −0.712% to −0.104%, respectively, where positive values denote lower independent-season error. Alternative management comparators yielded similarly small changes. Predictor distributions and calibration differed between transfer directions. These evaluations identify little incremental benefit from the measured phenotypes under the tested conditions, while demonstrating the importance of distinguishing yield ranking, calibration, and additional predictive information. Broader environmental validation is needed to determine when phenotyping improves forecasting beyond management expectations.

## 1. Introduction

Pre-harvest soybean (*Glycine max* (L.) Merr.) yield prediction can support production forecasting, risk assessment, and interpretation of crop performance before physiological maturity. Predictive accuracy within the conditions represented during model development, however, is not equivalent to transportability to a subsequent growing season. This distinction is particularly important in soybean systems managed across extended sowing windows. The sowing period affects exposure to seasonal weather and ENSO-related extremes, with consequences for yield and water productivity [1,2,3,4]. Cultivar maturity affects crop-stage timing and the environmental conditions encountered during yield formation [5,6,7], while genotype × environment interactions further limit extrapolation across production domains [8]. Low-latitude crop-model studies likewise show that sowing date and plant density cannot be interpreted independently of phenology and the environment [9,10,11]. Management information is therefore substantive prior predictive information rather than a set of nuisance variables. The contribution of in-season phenotyping should consequently be evaluated against a management comparator that represents the treatment structure known before crop measurements are collected [12,13,14,15].

Ground-measured crop traits provide a biologically plausible means of updating these management-based expectations. Leaf area, accumulated dry matter, branching, main-stem node number, and plant height describe complementary aspects of canopy construction, carbon acquisition, and crop architecture. Hyperspectral and multisensor studies have used related traits to predict soybean yield, maturity and stature and have demonstrated that phenotypic measurements can contain information beyond treatment labels [16,17,18]. Multitrait aerial and field phenotyping further emphasise parsimonious, interpretable feature sets rather than indiscriminate expansion of predictor dimensionality [19,20,21]. Trait relevance is also expected to change with development. Vegetative measurements characterise establishment and canopy expansion, whereas flowering, pod-set and grain-filling measurements increasingly reflect crop state during yield determination [22,23,24].

Nevertheless, yield formation in soybean emerges from a dynamic source–sink system rather than from a single static trait. Photosynthetic regulation, canopy light relationships, and reproductive source–sink sensitivity can modify the conversion of a crop state into final yield [25,26,27], whereas heat and water stress affect leaf area, growth, and reproductive performance [28,29,30,31]. Repeated phenotyping therefore presents an information-timing problem. Later measurements incorporate more of the realised growing-season history but reduce the forecast lead time, and correlated measurements at successive stages can either add genuinely new information or reproduce earlier crop-state signals. Ground and aerial studies have demonstrated the predictive potential of multitemporal soybean measurements [32,33,34], yet additional crop-state or spectral features have not invariably improved predictions beyond simpler representations [35]. The relevant predictive question is therefore not whether a stage-specific model can reproduce yield within a realised season but whether each newly available trait block corrects errors that remain after accounting for information already available.

Soybean yield prediction now spans municipality-scale forecasts based on weather and satellite time series [36,37,38], within-field and plot-scale models based on satellite or unmanned aerial vehicle data [39,40,41], image-based predictions from pods, leaves or multitemporal canopies [34,42], and large-scale transfer or tabular learning frameworks [43,44]. Hybrid and surrogate models increasingly combine biological structure, crop simulation, or feature selection with data-driven prediction [45,46,47]. Their spatial grains, forecast horizons, predictor sources, and validation targets differ markedly. High accuracy after random interpolation among plots from the same environments does not establish performance in a future season. Soybean-specific evidence shows that the cross-validation strategy materially affects apparent accuracy and transferability [48], and complete-year prediction studies likewise distinguish temporal forecasting from random record partitioning [49]. More broadly, row-wise partitions can underestimate error when observations are temporally, spatially, or hierarchically structured [50,51,52].

Validation should reproduce the intended deployment boundary. If the target is an unseen crop season, the decisive evaluation unit is the season rather than a randomly selected subplot. Imputation, scaling, variable filtering, tuning, and coefficient estimation must remain confined to development-season data to prevent independent-domain information from influencing model construction [53,54]. Even a rigorously separated model can lose accuracy or calibration when crop-state distributions change between development and evaluation seasons. Such a domain shift can expose a fitted model to trait combinations or ranges that were poorly represented during development, and its consequences can be directional because support from season A to season B does not need equal support from season B to season A [55,56,57]. The error magnitude should therefore be interpreted together with the calibration, mean bias, agreement and information supported by the available validation sample [58,59,60,61].

The field experiment analysed here previously supported a distinct agronomic study of seeding-rate responses across the same sowing window [13]. That study evaluated treatment means, final yield components, compensatory plasticity, and management recommendations. The present study addresses a different predictive question: whether direct measurements collected at V4, R2, R5 and R6 provide incremental cross-season information beyond the development-season grain yield expectation for the corresponding sowing-period × cultivar × seeding-rate combination.

Our objective was to quantify the stage-resolved incremental value of a compact set of ground-measured morphophysiological traits for soybean grain yield prediction under reciprocal independent-season evaluation between 2021/22 and 2022/23 at one site. We compared a development-season mean, a management-combination mean and cumulative phenotype-augmented models through V4, R2, R5, and R6. We hypothesised that at least one phenotypic information gate would provide a reproducible reduction in independent-season RMSE, that the contribution would be stage-dependent, and that calibration and error would differ between transfer directions in association with predictor-domain shift. We interpreted the continuous paired RMSE changes and used 2%, 5%, and 10% gains as empirical reference levels. We also evaluated whether the results depended on the management comparator and compared the compact representation directly with broader plant-scale representations. This design targets the additional predictive information supplied by stage-specific subplot measurements beyond management-combination means.

## 2. Results

### 2.1. Analytical Sample, Yield Distribution, and Predictor-Domain Shift

**Figure 1:**
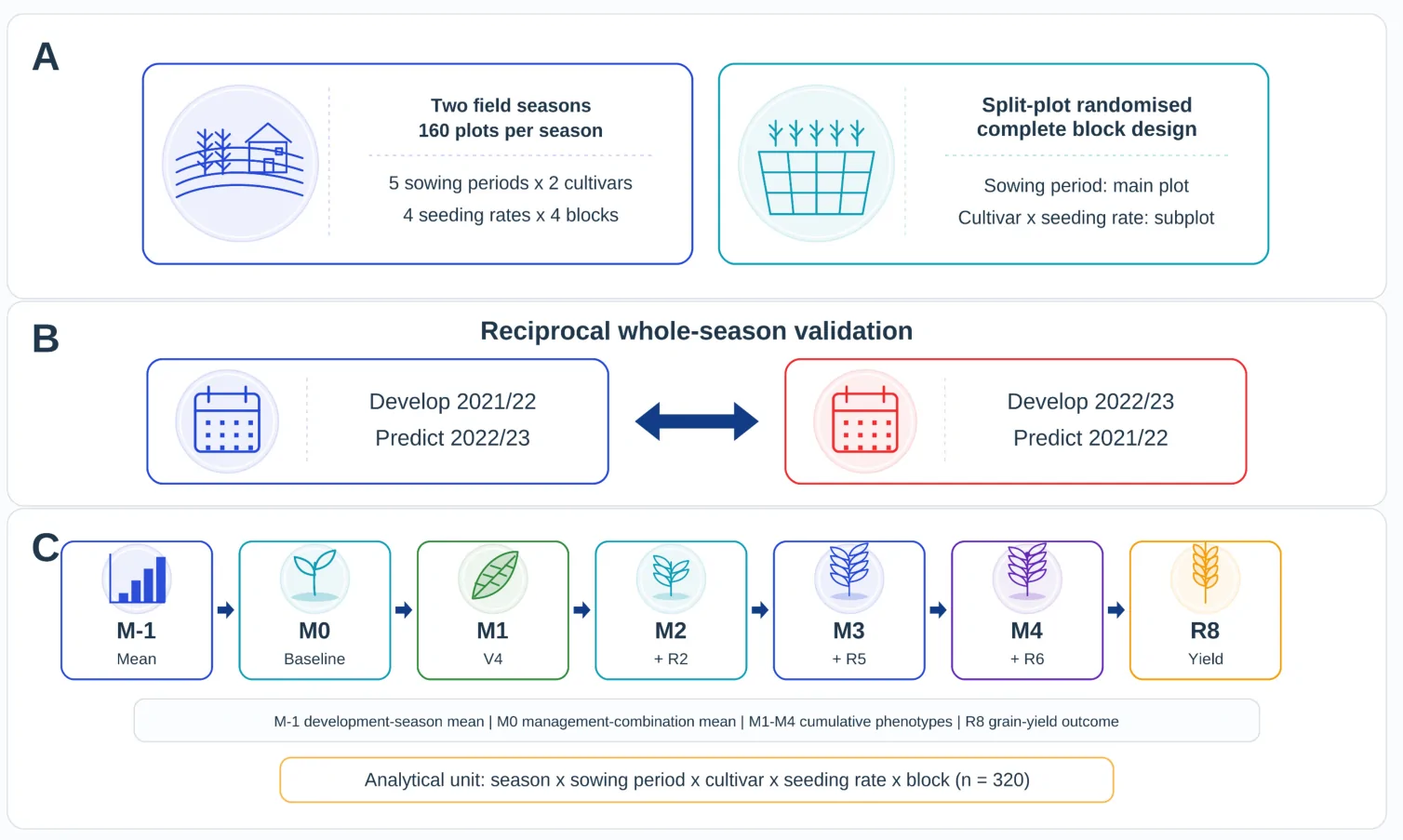
Experimental design and reciprocal temporal validation. Panel (**A**) summarises the two-season split-plot randomised complete block design: five sowing periods were assigned to main plots, and the factorial combination of two cultivars and four seeding rates was assigned to subplots within four blocks, resulting in 160 observations per season. Panel (**B**) depicts reciprocal temporal transfer, with each season serving in turn as the development and independent season. Panel (**C**) defines the information sequence from the development-season mean (M−1) and management-combination mean (M0) to cumulative phenotype gates through V4 (M1), R2 (M2), R5 (M3), and R6 (M4). The prediction window ended at R6, and the grain yield measured at R8 was the sole outcome. Created in BioRender. Falcioni, R. (2026) https://BioRender.com/qxs0jeh.

**Figure 2:**
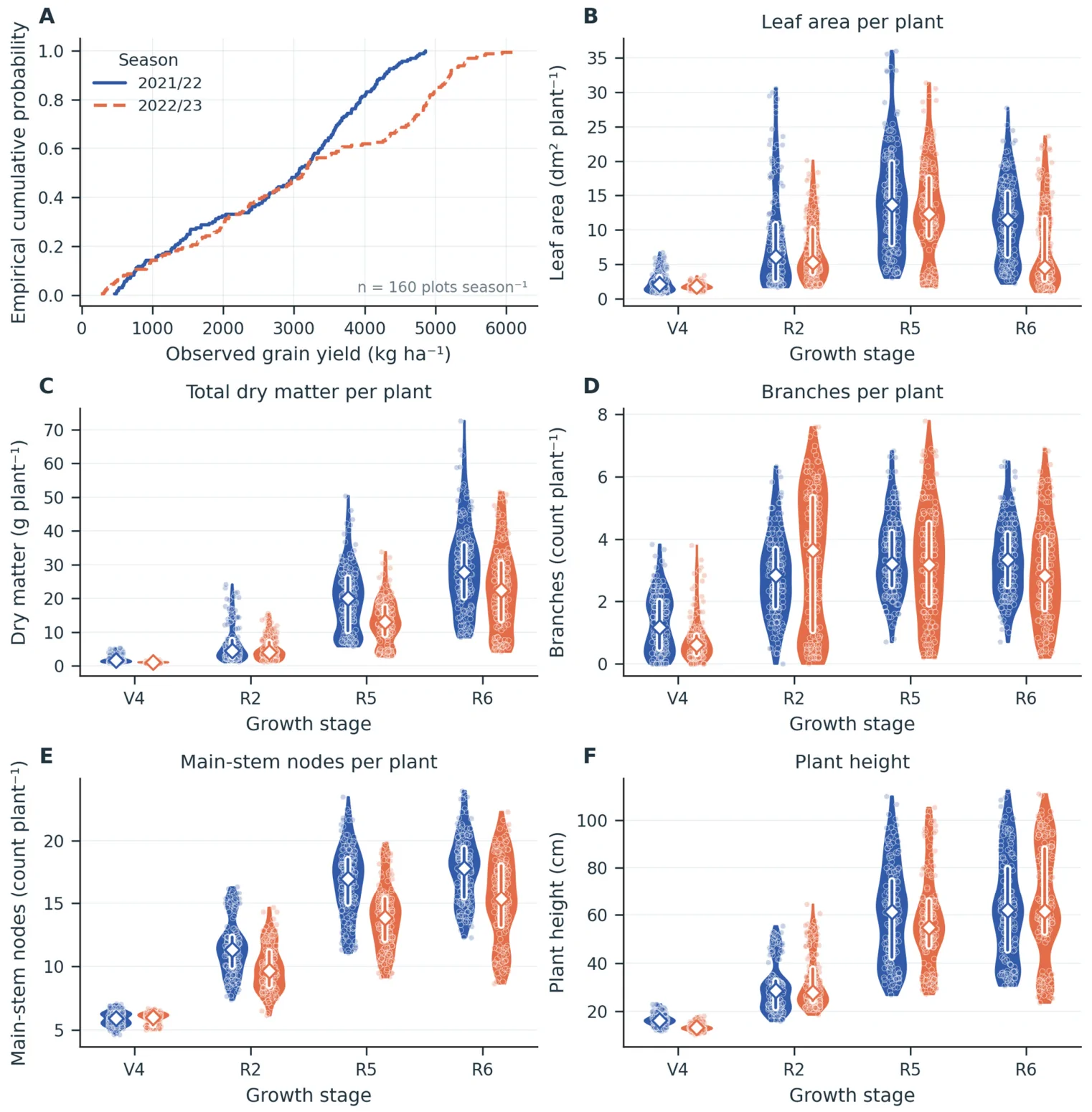
Observed yield and stage-resolved phenotypic distributions in the two seasons. Panel (**A**) shows the empirical cumulative distributions of the observed grain yield (n = 160 plots per season). Panels (**B**–**F**) show the observed distributions of leaf area per plant, total plant dry matter, branches per plant, architecture-assessment main-stem nodes, and plant height at V4, R2, R5, and R6. Split violin layers distinguish 2021/22 from 2022/23; points are individual plots, internal boxes identify the median and interquartile range, and each trait retains its measurement unit.

The reciprocal validation design and stage-resolved information sequence are summarised in Figure 1. The experiment included 320 subplot-level observations, which were equally divided across seasons. The predictor definitions are given in Table S1. The average grain yield was 2752.4 kg ha^−1^ (SD 1286.3; range 462.0–4862.4) in 2021/22 and 3152.9 kg ha^−1^ (SD 1654.9; range 270.0–6144.0) in 2022/23 (Figure 2 and Figure S1; Table 1).

The predictor distributions shifted directionally between seasons (Figure 2, Figures S2 and S3; Tables S2 and S3), whereas the within-season correlation structure of the primary predictors is shown in Figure S4. For 2021/22 → 2022/23, the median absolute standardised mean difference (SMD) across the 20 primary stage–trait variables was 0.430, 16 variables had at least one independent-season observation outside the development-season range, and the largest outside-range proportion was 21.875%. The V4 plant height had the greatest absolute SMD (1.220). The stage-specific median absolute SMDs were 0.568 at V4, 0.258 at R2, 0.194 at R5, and 0.498 at R6; the corresponding mean outside-range proportions across the five traits were 4.125%, 3.375%, 11.500%, and 15.750%, respectively.

For 2022/23 → 2021/22, the median absolute SMD was 0.472, 17 variables showed some range extrapolation, and the largest outside-range proportion was 42.500%. V4 had the largest stage-level shift, with a median absolute SMD of 1.297 and a mean outside-range proportion of 22.625%. The V4 total dry matter per plant had an SMD and a scaled Wasserstein distance of 3.490, a variance ratio of 17.440, and 42.500% of the independent-season values outside the development-season range, respectively. The median absolute SMDs at R2, R5, and R6 were 0.330, 0.208, and 0.529, respectively.

### 2.2. Absolute Cross-Season Performance

**Figure 3:**
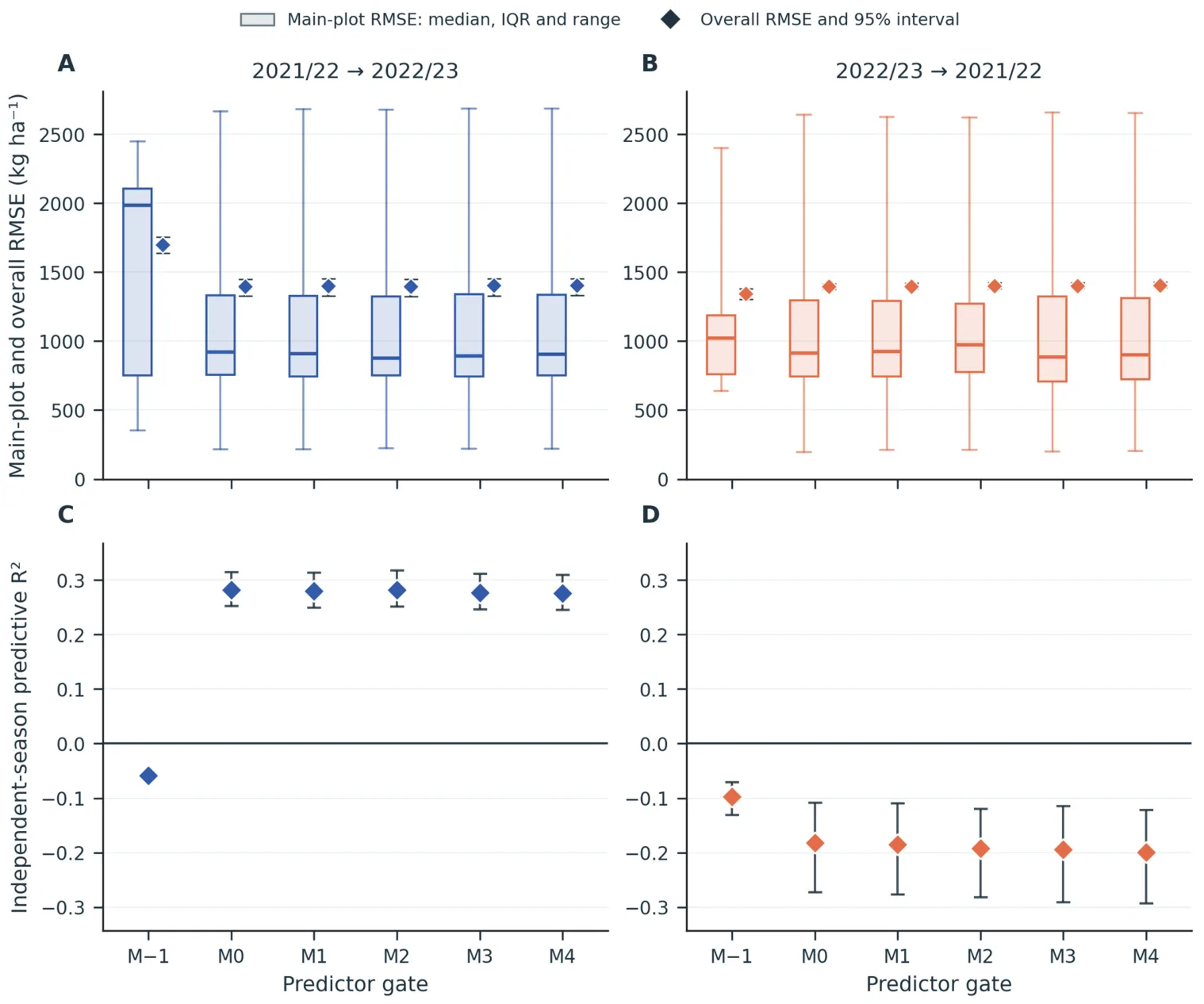
Independent-season error and predictive performance across information gates. Panels (**A**,**B**) show main-plot RMSE distributions for 2021/22 → 2022/23 and 2022/23 → 2021/22, respectively. Boxes span the interquartile range, central lines show medians, and whiskers show the full range across 20 block × sowing-period main plots per gate. Diamonds denote overall RMSE estimates. Panels (**C**,**D**) show predictive R^2^ for the corresponding directions. Error bars are conditional within-season whole-block bootstrap 95% intervals; each direction comprises 160 subplots within four complete blocks.

**Figure 4:**
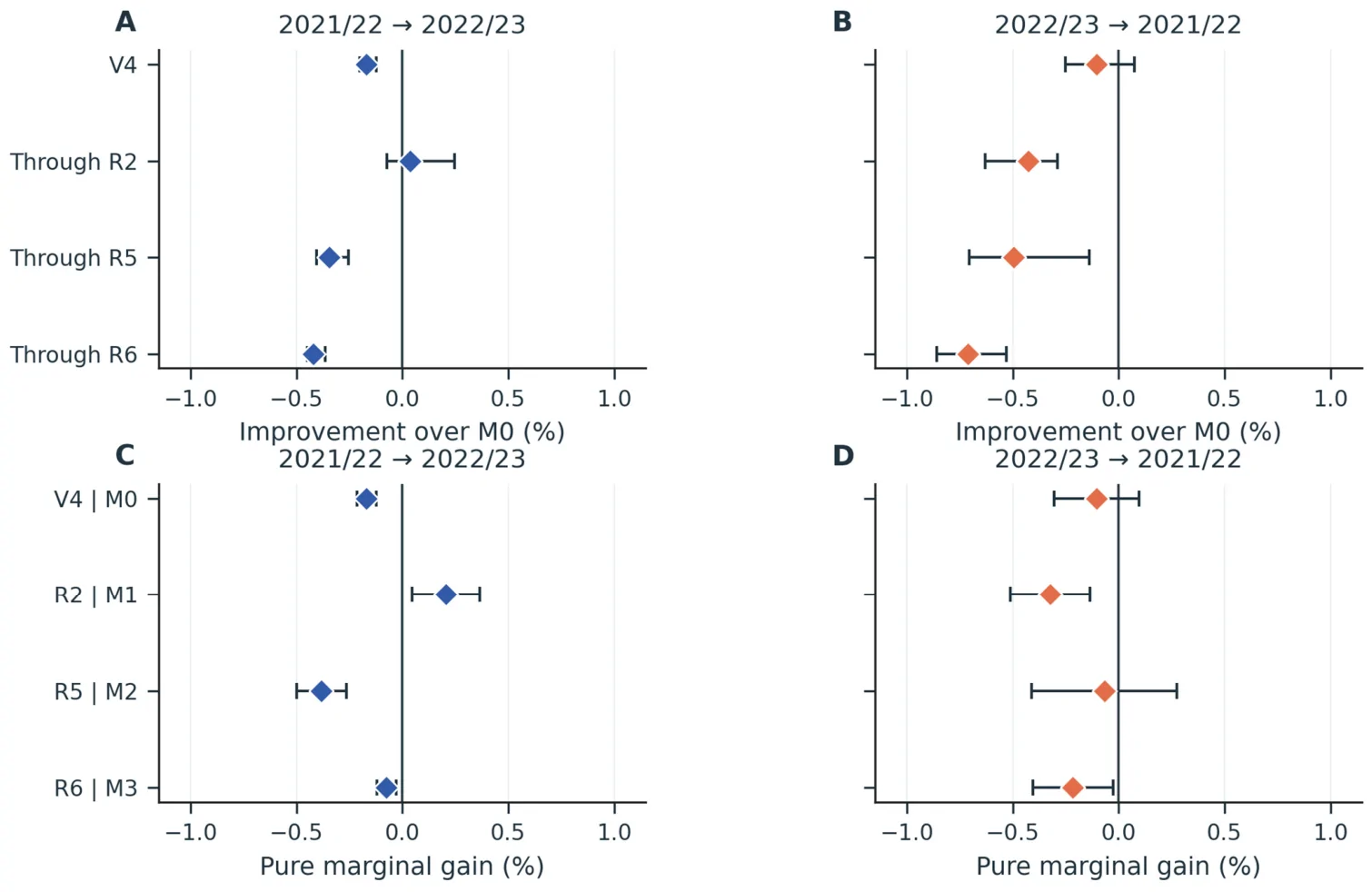
Cumulative and sequential incremental value of the phenotypes. Panels (**A**,**B**) show paired RMSE gains for M1–M4 relative to M0 in the 2021/22 → 2022/23 and reciprocal directions, with pointwise conditional within-season whole-block bootstrap 95% intervals. Panels (**C**,**D**) show the successive contrasts M1−M0, M2−M1, M3−M2, and M4−M3, with simultaneous 95% max-statistic intervals. Positive values favour the richer gate.

The absolute prediction error differed between transfer directions, whereas cumulative phenotypic augmentation produced little change within either direction (Figure 3 and Figure 4; Table 2). For 2021/22 → 2022/23, M0 had an RMSE of 1398.1 kg ha^−1^ (conditional within-season whole-block bootstrap 95% CI 1325.8–1448.1), an nRMSE of 44.3% (42.7–45.6), an MAE of 1057.0 kg ha^−1^ (1009.2–1110.1), and a predictive R^2^ of 0.282 (0.252–0.315). Across M1–M4, the RMSE ranged from 1397.6 to 1403.9 kg ha^−1^, and the predictive R^2^ ranged from 0.276 to 0.282. M4 had an RMSE of 1403.9 kg ha^−1^ (1330.6–1454.5) and a predictive R^2^ of 0.276 (0.245–0.310). For 2022/23 → 2021/22, M0 had an RMSE of 1394.3 kg ha^−1^ (1377.9–1416.4), an nRMSE of 50.7% (49.6–51.5), an MAE of 1052.7 kg ha^−1^ (1032.9–1074.6), and a predictive R^2^ of −0.182 (−0.273 to −0.109). The M1–M4 RMSE values ranged from 1395.8 to 1404.2 kg ha^−1^, and the predictive R^2^ ranged from −0.185 to −0.199. M4 had an RMSE of 1404.2 kg ha^−1^ (1387.1–1426.5) and a predictive R^2^ of −0.199 (−0.293 to −0.122). Complete design-aware intervals are reported in Table 2 and Table S4, and grouped inner-validation profiles are shown in Figure S5.

Within-season nested validation yielded M0 RMSEs of 244.7 and 280.3 kg ha^−1^ for development in 2021/22 and 2022/23, respectively; the corresponding independent-season RMSEs were 1398.1 and 1394.3 kg ha^−1^. M4 yielded nested RMSEs of 246.7 and 281.1 kg ha^−1^, compared with independent-season RMSEs of 1403.9 and 1404.2 kg ha^−1^. Thus, phenotype augmentation did not materially reduce the marked gap between within-season validation and season transfer. The nested M1–M4 RMSEs ranged from 242.6 to 247.7 kg ha^−1^ for development in 2021/22 and from 281.0 to 284.0 kg ha^−1^ for development in 2022/23 (Table 2 and Table S5).

**Figure 5:**
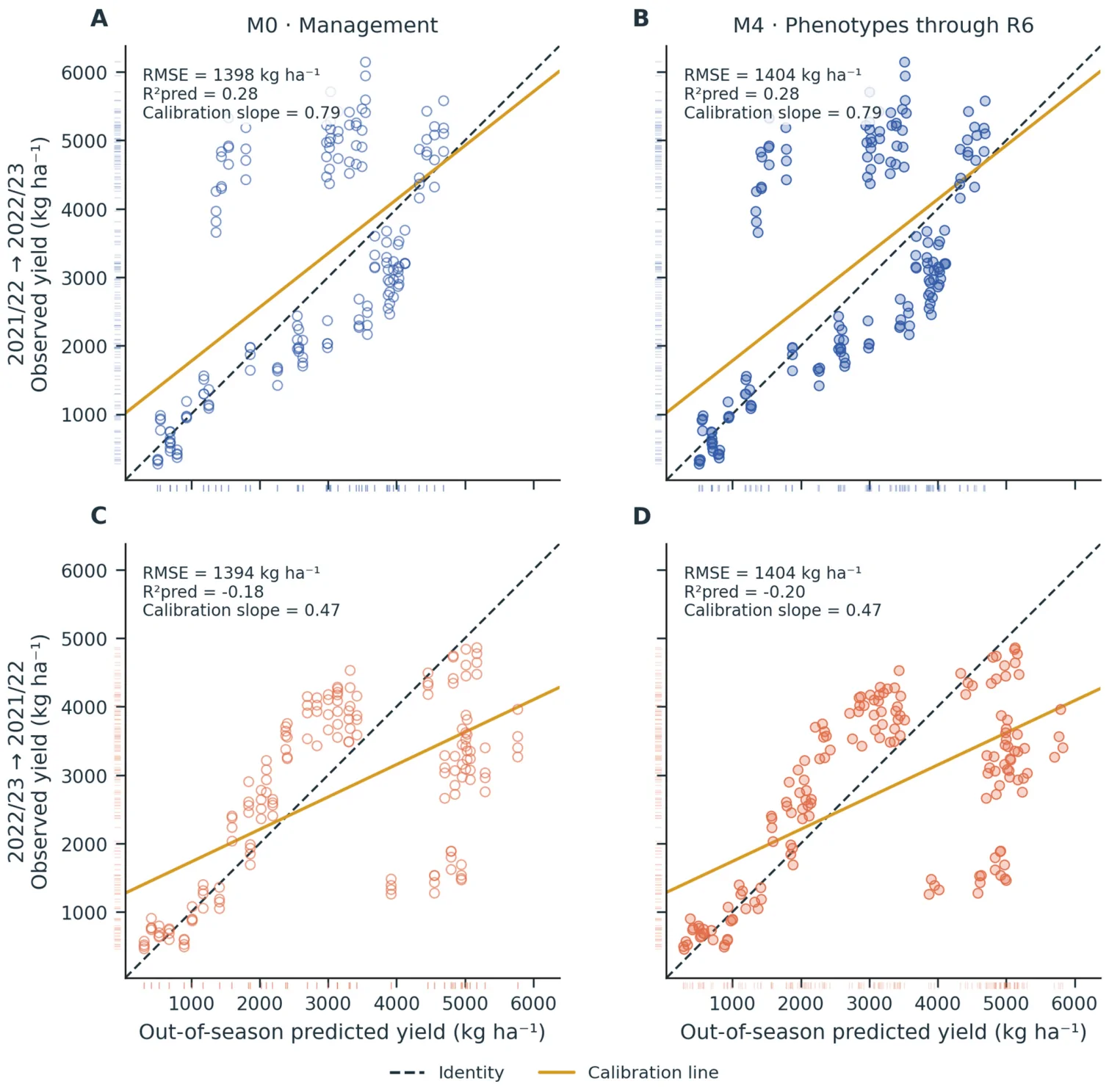
Observed and predicted grain yield for management and terminal phenotype models. Panels (**A**,**B**) show M0 and M4 for 2021/22 → 2022/23; panels (**C**,**D**) show the reciprocal direction. Points are independent-season subplot observations; the dashed line denotes 1:1 agreement, and the fitted line describes calibration. Each panel contains 160 subplots within four blocks.

The observed-versus-predicted patterns for M0 and M4 are shown in Figure 5. When the two independent-season prediction vectors were combined, M0 had an RMSE of 1396.2 kg ha^−1^, a predictive R^2^ of 0.123, and a CCC of 0.558. The combined RMSE increased from 1398.1 kg ha^−1^ at M1 to 1404.1 kg ha^−1^ at M4, whereas the predictive R^2^ decreased from 0.121 to 0.113 (Table S4). These combined summaries complement, but do not replace, direction-specific evaluation. These pooled statistics describe the two prediction vectors jointly. They do not represent an average transfer success or an additional independent validation, because pooling changes the reference yield distribution and combines models with different calibration and bias. Interpretation therefore rests on the two direction-specific evaluations.

### 2.3. Incremental Phenotype Contribution and Reference Gains

Relative to M0, the change in the RMSE for every cumulative phenotype gate remained below 1% in both directions (Figure 4; Table 3). For 2021/22 → 2022/23, the RMSE improvements were −0.168% (95% CI −0.200 to −0.124) at M1, 0.038% (−0.073 to 0.247) at M2, −0.345% (−0.405 to −0.254) at M3, and −0.419% (−0.449 to −0.363) at M4. For 2022/23 → 2021/22, the corresponding changes were −0.104% (−0.252 to 0.075), −0.427% (−0.630 to −0.288), −0.495% (−0.707 to −0.138), and −0.712% (−0.857 to −0.531). The combined reciprocal changes were −0.136%, −0.194%, −0.420%, and −0.565% for M1–M4, respectively. The changes in the MAEs of M0 were similarly small (Table S6).

None of the eight direction-specific point estimates reached a 2%, 5%, or 10% reduction in the RMSE. For every direction × gate comparison, none of the 2000 whole-block bootstrap resamples attained any of these thresholds (Table 3 and Table S6). These finite-resample counts are reported directly and are not interpreted as parameter probabilities. As a finite-support robustness check, all 256 ordered four-block resamples, corresponding to 35 unique block-multiplicity configurations, were also enumerated exactly; no configuration attained a 2%, 5%, or 10% gain in either direction at any gate. All three illustrative reference gains yielded the same threshold-attainment result within this experiment.

### 2.4. Management Structure and Stage-Marginal Contributions

The M0 management-combination mean reduced the RMSE relative to the M−1 development-season mean by 17.647% for 2021/22 → 2022/23 and changed the RMSE by −3.789% for 2022/23 → 2021/22; the combined management increment was 8.794% (Table 4 and Table S6). Because M0 estimated 40 management-combination means from four blocks per season, robustness was evaluated with a less saturated additive management model (M0A) containing main effects for the sowing period, cultivar, and seeding rate. M0A achieved RMSE values of 1376.7 and 1383.9 kg ha^−1^ in the two directions. Adding the compact phenotype gates to M0A changed the RMSE by −0.075%, −0.165%, −0.622%, and −0.380% for 2021/22 → 2022/23 and by 0.000%, −0.622%, −0.110%, and −0.243% for 2022/23 → 2021/22, respectively. The small incremental contribution was therefore not a consequence of using only the cell-specific management comparator.

The pure stage-marginal changes quantified the information added at each phenological transition. For 2021/22 → 2022/23, the RMSE changes were −0.168% for M1–M0, 0.206% for M2–M1, −0.382% for M3–M2, and −0.075% for M4–M3. For 2022/23 → 2021/22, the corresponding changes were −0.104%, −0.323%, −0.068%, and −0.215%, respectively. The combined marginal changes were −0.136%, −0.058%, −0.225%, and −0.145%. Simultaneous confidence intervals for the four-stage additions and counts of bootstrap resamples attaining 2%, 5%, and 10% improvements are provided in Table 4 and Table S6, respectively.

### 2.5. Phenotypic Correction, Calibration, and Management-Stratum Error

**Figure 6:**
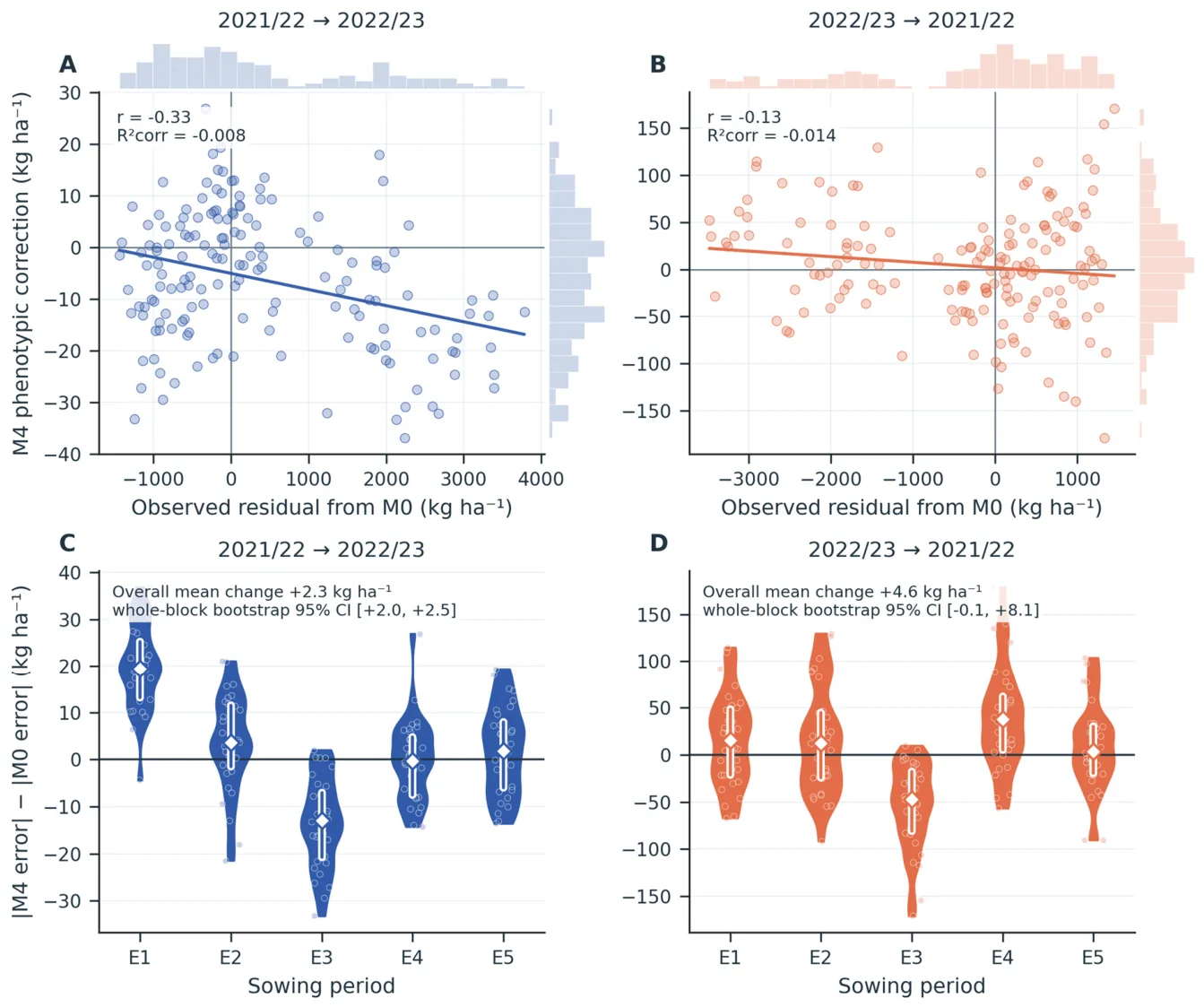
Phenotypic corrections and paired changes in absolute error. Panels (**A**,**B**) relate the grain yield residual remaining after M0 to the terminal phenotypic correction M4−M0 for each independent-season plot. Marginal histograms show the residual and corrected distributions; the coloured line is a descriptive least-squares fit, and the horizontal and vertical guides denote zero. Pearson’s r and the zero-correction skill score R^2^_corr_ = 1 − SSE(M4)/SSE(M0) are annotated. Panels (**C**,**D**) show the sowing-period distributions of |eM4|−|eM0|. Points are plot observations; internal marks identify the median and interquartile range, and annotations give the overall mean change with its conditional within-season whole-block bootstrap 95% interval. Negative values favour M4. Conditional whole-block intervals for the correction correlations are reported in Table S7.

The zero-correction skill, R^2^_corr_ = 1 − SSE(Mk)/SSE(M0), compares the squared error after phenotype augmentation with that of M0. Zero denotes no change, positive values indicate reduced error, and negative values indicate increased error; this score is not a squared correlation. For 2021/22 → 2022/23, the Pearson correlations between phenotypic corrections and M0 residuals were 0.093 (95% interval 0.035–0.161), 0.090 (0.035–0.181), −0.098 (−0.156 to 0.021), and −0.327 (−0.410 to −0.245) for M1–M4, respectively. For 2022/23 → 2021/22, the corresponding correlations were −0.118 (−0.201 to −0.009), −0.418 (−0.484 to −0.355), −0.013 (−0.131 to 0.063), and −0.135 (−0.245 to −0.064). Weak positive alignment at the first two gates in the first direction therefore did not develop into a consistently beneficial cumulative correction. The corrections are shown in Figure 6, and the skill estimates and correlation intervals are given in Table S7.

**Figure 7:**
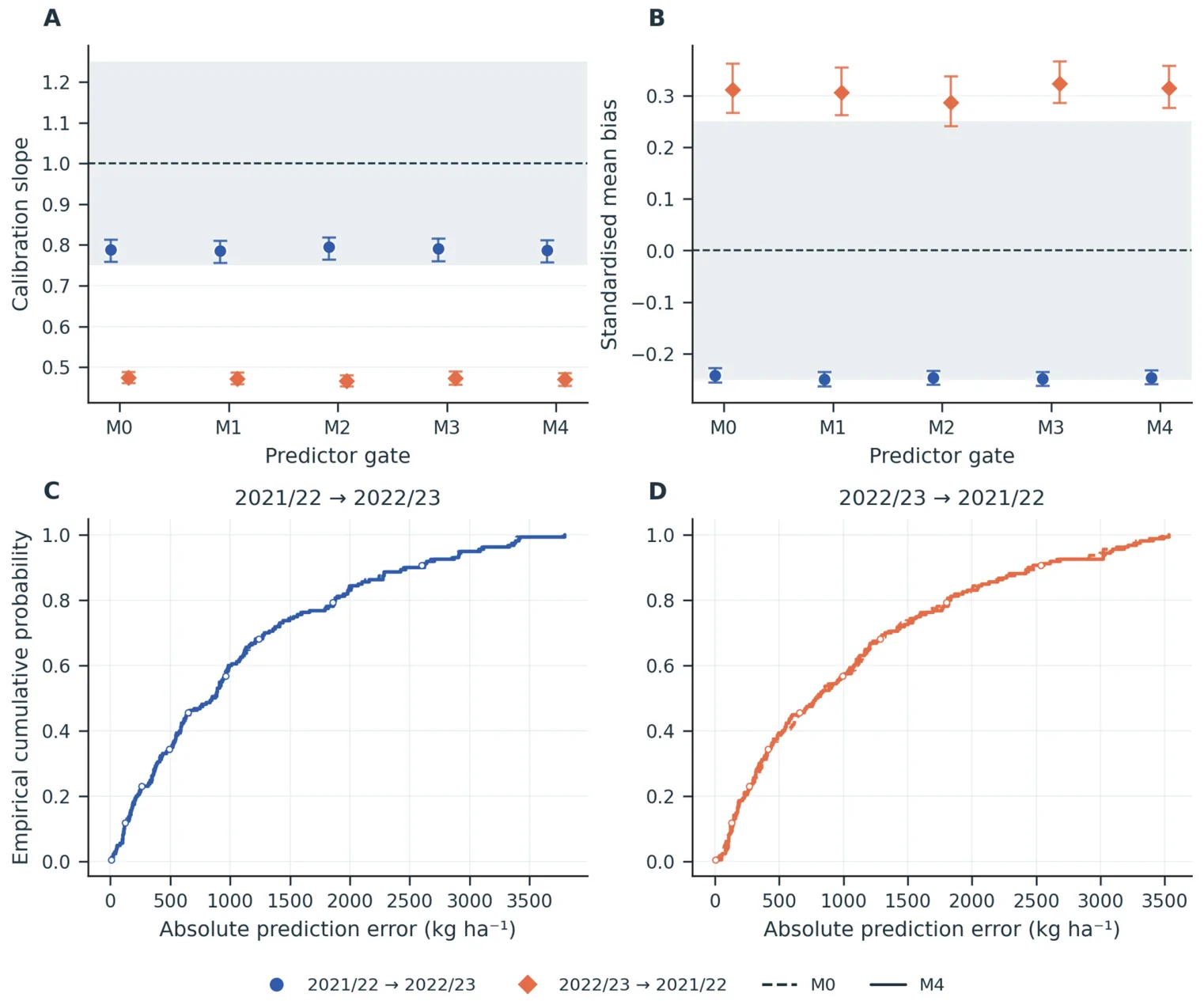
Calibration and absolute-error reliability across predictor gates. Panels (**A**,**B**) show the calibration slope and standardised mean bias for M0–M4 in both transfer directions; points are estimates, and intervals are conditional within-season whole-block bootstrap 95% intervals. Dashed lines mark ideal values of 1 and 0. The shaded zones are descriptive reference bands. Panels (**C**,**D**) show empirical cumulative distributions of absolute prediction error for M0 and M4 (n = 160 independent-season plots per gate and direction). In panels (**C**,**D**), dashed and solid curves represent M0 and M4, respectively, and largely overlap.

**Figure 8:**
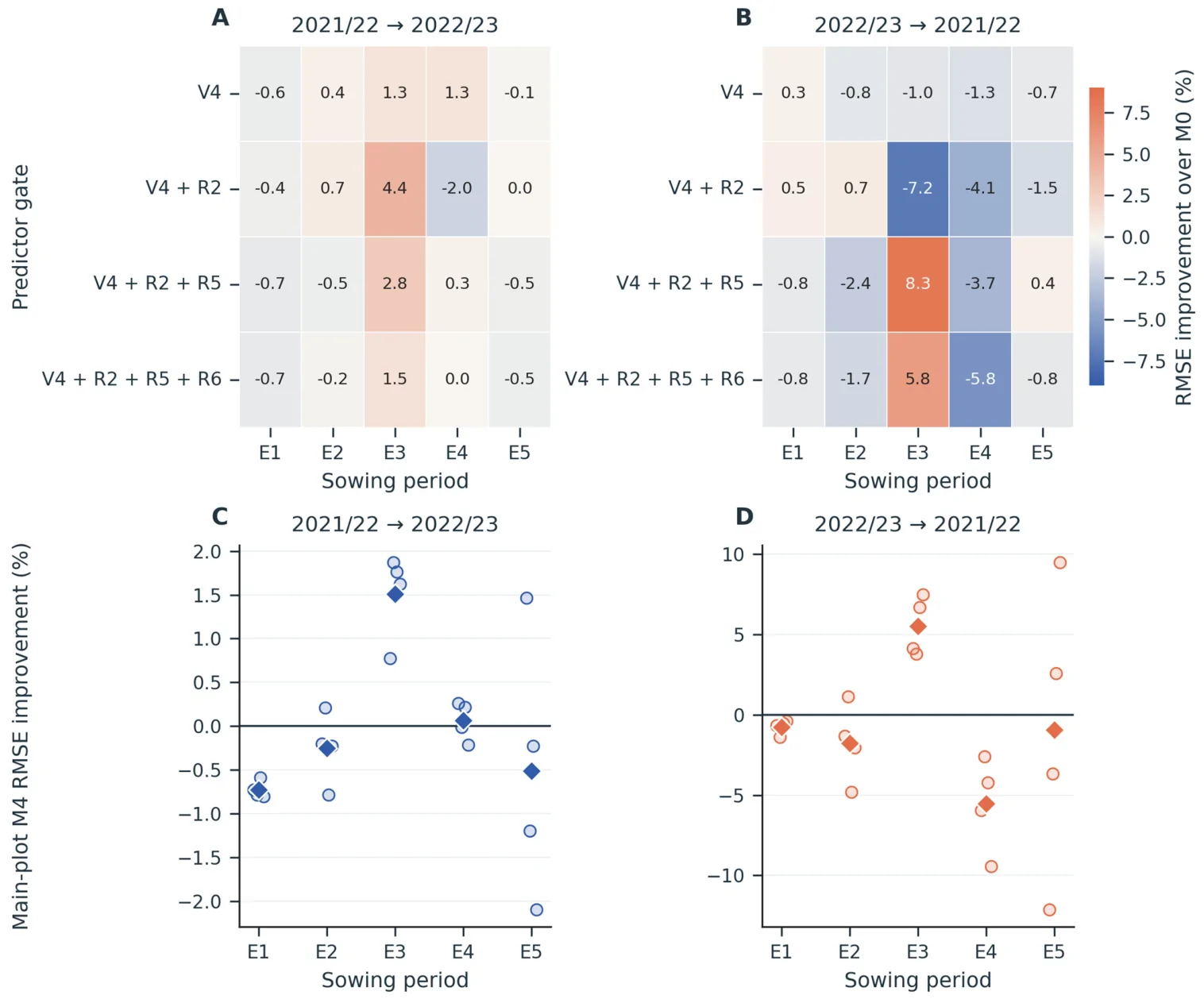
Sowing-period heterogeneity in incremental phenotype contribution. Panels (**A**,**B**) show the paired RMSE gains of M1–M4 relative to M0 within sowing periods E1–E5 in each transfer direction. Annotated cells are descriptive estimates from 32 independent-season subplot observations within four main plots per period. Panels (**C**,**D**) show the four block × sowing-period main-plot M4-versus-M0 RMSE changes for each period; diamonds are descriptive means. Positive values favour phenotype augmentation. E3 patterns are hypothesis-generating and do not establish a reproducible sowing-period-specific phenotype benefit. The summaries describe heterogeneity without period-specific model tuning. In panels (**C**,**D**), blue and orange symbols denote the 2021/22 → 2022/23 and 2022/23 → 2021/22 transfer directions, respectively. Circles represent the individual main-plot estimates, whereas diamonds represent their descriptive means.

The calibrations differed across the transfer directions (Table 2; Figure 7). In 2021/22 → 2022/23, the calibration-slope point estimates ranged from 0.786 to 0.795 for M0–M4, and the standardised mean bias ranged from −0.249 to −0.242. In 2022/23 → 2021/22, the slopes ranged from 0.466 to 0.474, and the standardised mean bias ranged from 0.286 to 0.323. The CCC remained between 0.561 and 0.566 across M0–M4 in each direction (Table S4). Direction-specific phenotype contributions varied among sowing periods but did not show a common sequence (Figure 8; Table S8). The largest descriptive changes occurred in E3, but each period-specific estimate comprised 32 observations within four main plots and was interpreted descriptively.

Despite negative predictive R^2^ in the reciprocal direction, yield ranking remained positively associated with observations: Spearman’s ρ was 0.588 (0.574–0.609) for M0 and 0.577 (0.559–0.598) for M4 in 2022/23 → 2021/22. In 2021/22 → 2022/23, ρ was 0.572 (0.541–0.599) for M0 and 0.571 (0.540–0.598) for M4. Thus, negative predictive R^2^ did not represent complete loss of ranking; moderate rank agreement coexisted with substantial calibration and absolute-error deficiencies. Similar ranking for M0 and M4 also indicates that phenotypes supplied little additional discrimination under this transfer design (Table S7).

### 2.6. Sensitivity Analyses

Sensitivity analyses were performed exclusively from direct plant-scale measurements available at V4–R6. The evaluated families included stage-only models, partial least-squares regression, expanded direct plant-scale and organ-component representations, alternative direct architecture measurements, log-scale outcome modelling, and restriction to sowing periods E1–E4 (Figures S6–S10; Table S9). Across these specifications, paired RMSE gains remained small relative to their matched management comparator. Partial least squares, the expanded direct plant-scale basis, and the organ-component basis produced negative RMSE-gain estimates in every direction and gate. The largest positive RMSE-gain estimate occurred under log-scale outcome modelling, whereas restriction to E1–E4 retained the complete-domain directional pattern.

**Figure 9:**
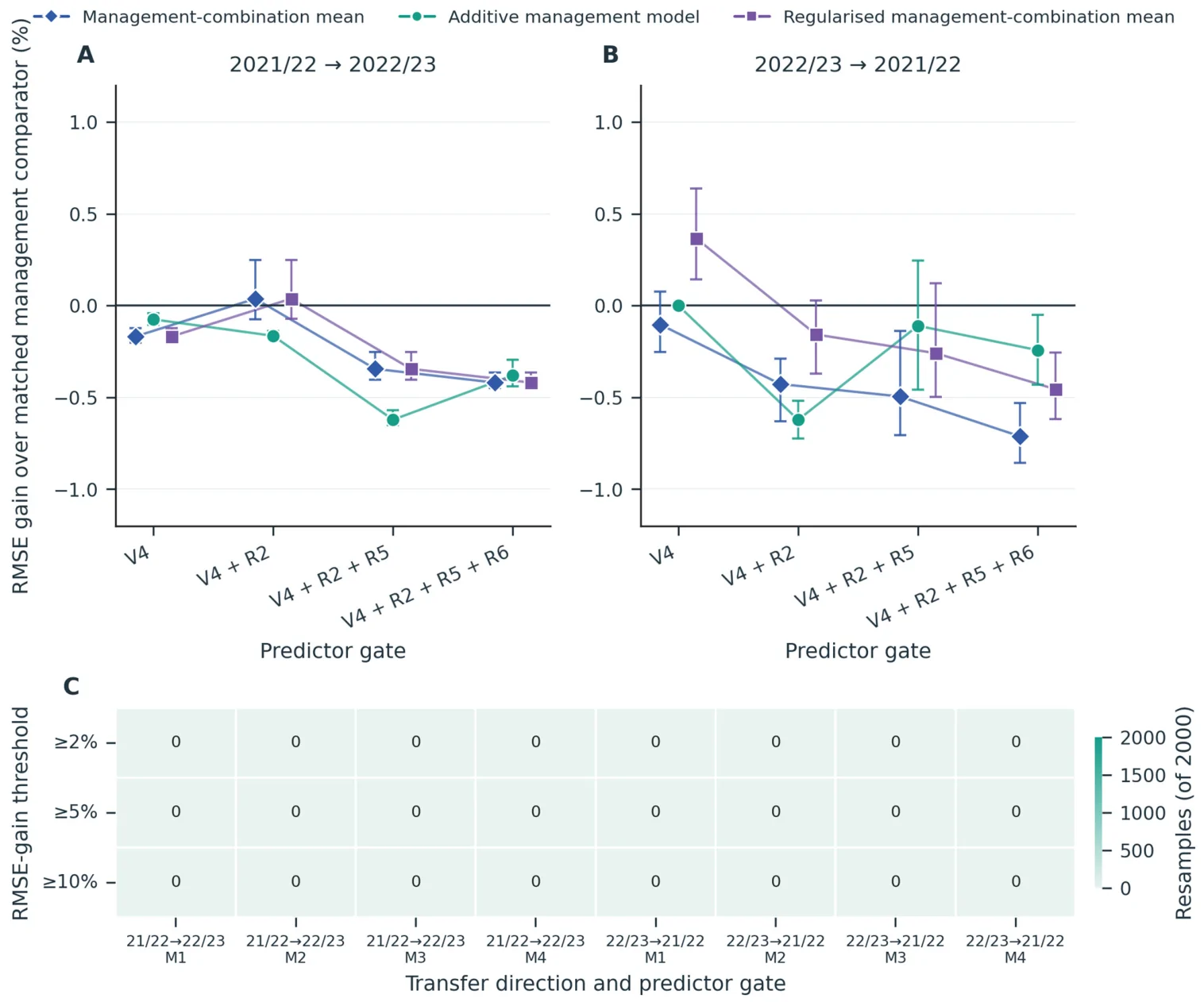
Robustness to management comparators and RMSE-gain thresholds. Panels (**A**,**B**) compare the incremental RMSE gain of the compact cumulative phenotype gates relative to three matched management expectations: the management-combination mean (M0), an additive main-effects management model (M0A), and a ridge-regularised management-combination model (M0P). Points are direction-specific estimates, and intervals are conditional within-season whole-block bootstrap 95% intervals. Positive values favour phenotype augmentation. Panel (**C**) reports the numbers of 2000 whole-block resamples attaining RMSE gains of at least 2%, 5%, and 10% for the primary M0 comparison in each direction and gate. The comparator and threshold analyses retained the same magnitude interpretation.

Direct contrasts between valid phenotype representations are reported separately from their comparisons with M0 (Table S9). They show whether a broader direct plant-scale representation changed performance relative to the compact basis, rather than inferring equivalence because both were individually compared with management. Across the sensitivity matrix, threshold support is reported as counts out of 2000 whole-block bootstrap resamples at 2%, 5%, and 10%. The alternative sowing-period-stratified main-plot bootstrap yielded the same substantive interpretation (Figure 9; Table S9). Residual diagnostics are shown in Figure S11.

## 3. Discussion

### 3.1. Incremental Phenotype Information Under Reciprocal Seasonal Validation

The principal finding was the small change in independent-season error after the measured crop states were added to the management comparator. Leaf area, total dry matter, branches, main-stem nodes and plant height through V4, R2, R5, or R6 changed RMSE by less than 1% in either transfer direction (Table 3; Figure 4). Stage-marginal contrasts likewise did not identify a phenological addition that improved performance in both directions. None of the primary point estimates or whole-block resamples attained the illustrative 2%, 5%, or 10% gain levels. These estimates characterise the fitted models and the two observed evaluation seasons. Their conditional intervals do not establish a tight upper bound on phenotype utility in a population of future seasons, locations or genotypes.

The incremental formulation helps explain why biologically informative measurements produced little additional predictive gain. M0 already represented the mean differences among the tested management combinations, so the phenotype models had to predict the remaining yield deviations rather than recover those treatment contrasts. Their corrections were small and showed limited or negative alignment with the independent-season M0 residuals (Figure 6; Table S7), indicating that the fitted residual relationships did not transfer reliably. Correlation among traits and stages can further limit the new information supplied by later measurements. Predictor-domain shift, changing crop-state–yield relationships and plant-sampling error are non-exclusive explanations consistent with the results, but the present design cannot estimate their separate contributions. This distinction between a biologically relevant association and an incremental transferable prediction is central to interpreting phenotyping models [48,50,54].

### 3.2. Management Expectations and the Value of a Second Comparator

The primary M0 comparator transferred the development-season mean of each sowing period × cultivar × seeding rate combination. It therefore represented the factorial treatment structure known before phenotyping and prevented crop traits from receiving predictive credit merely because they encoded differences already specified at sowing. This is an agronomically relevant benchmark because sowing timing, maturity and plant density structure affect soybean yield responses across environments [1,3,5,15]. The corresponding estimand is intentionally incremental: it asks whether crop measurements update a treatment-specific expectation, not whether they can reconstruct treatment contrasts.

The cell means can nevertheless be sensitive when each treatment combination is represented by four blocks. The additive M0A robustness model reduced this specificity by estimating the shared main effects of the sowing period, cultivar and seeding rate. Its absolute RMSE was slightly lower than that of M0 in both directions, yet the phenotype-associated changes ranged only from −0.622% to 0.000%. Thus, the central inference was retained under a comparator that pooled information across management combinations. This agreement is important: the limited incremental signal did not arise solely because the primary reference was a saturated set of 40 unshrunk cell means.

Transported management information itself was directional. Relative to the development-season grand mean, M0 substantially improved the prediction for 2021/22 → 2022/23 but not for the reciprocal direction. M0 achieved a predictive R^2^ = 0.282 when predicting 2022/23 and a predictive R^2^ = −0.182 when predicting 2021/22, although the CCC was similar in the two directions. The calibration slopes of approximately 0.79 and 0.47 and opposing mean biases show that direction-specific differences involved both dispersion and location. A negative predictive R^2^ indicates that the squared error exceeded that obtained from the observed independent-season mean; it does not imply the absence of rank agreement. Reporting error, calibration, bias, and concordance together therefore provides a more complete account of cross-season performance [58,59].

### 3.3. Predictor Domain Shift and Calibration Asymmetry

The predictor diagnostics provide a coherent, although not causal, context for the calibration asymmetry. When models were developed in 2022/23 and transferred to 2021/22, the V4 trait block occupied a substantially different domain. Its median absolute SMD was 1.30, and on average, 22.6% of the independent-season observations were outside the corresponding development-season ranges (Figure 2; Table S3). The total dry matter per plant was especially displaced (SMD 3.49; variance ratio 17.44; 42.5% outside range), as were the leaf area per plant and plant height. Because every cumulative gate retained V4, the same early-stage distributional mismatch remained part of all four predictor sets; however, this observation alone does not establish that V4 caused the transfer error.

The reciprocal direction presented a different support pattern, with less extreme V4 displacement but more frequent range extrapolation for several R5 and R6 variables. Consequently, the stronger 2021/22 → 2022/23 predictive R^2^ should not be interpreted as evidence that 2021/22 is intrinsically a better development season. Developmentally standardised shift measures are directional: broad coverage of season B by season A does not imply reciprocal coverage. Similar covariate mismatches constrain temporal, climatic and spatial transfer in crop-yield modelling and motivate support diagnostics before adaptation or recalibration is attempted [55,56,57].

Domain shift remains a diagnostic rather than a proof of mechanism. During the two seasons, changes in the predictor distributions, yield distributions, and unmeasured environmental processes occurred together. The differences in V4 coincided with the lower reverse-direction calibration slope, but its cause could not be identified. Greater model flexibility would not necessarily resolve this limitation because flexible estimators can amplify extrapolation where predictor support is weak [50]. Transfer-oriented development should therefore establish the intended evaluation domain and quantify common support before algorithmic complexity is increased [62,63].

### 3.4. Biological Relevance and Temporal Invariance Are Different Properties

The small observed increments do not imply that leaf area, biomass, branching, nodes, or plant height are biologically unimportant. These traits characterise soybean canopy development and yield formation [22,23], and experimental interventions affecting photosynthesis, canopy leaf area, source–sink balance and reproductive heat exposure can change yield [25,26,27,28]. Here, the fitted phenotype-based corrections provided little additional accuracy beyond management expectations in the two evaluated transfers. This result concerns a particular prediction target and validation design, rather than the usefulness of the simple traits for physiological explanation, treatment comparison, or selection in other germplasm and environments.

Several features of soybean development make such instability plausible. The same phenotype at a canonical stage can arise from different preceding temperatures, radiation and water histories. Crop-state measurements cannot encode all the subsequent conditions affecting pod retention, seed number, and grain filling, and compensatory yield components can translate similar biomass or architecture into different reproductive outcomes. Moreover, stage-based alignment does not equalise the calendar or thermal interval to maturity across sowing periods and cultivars. Later measurements can therefore approach yield determination without necessarily acquiring a more transportable relationship with final yield [1,5,22,27].

The destructive sampling design also defines the biological interpretation of the sequence. Each gate adds sample means from a new cohort within the same subplot; it does not reconstruct the physiological history of individual plants. Variation among the sampled plants may contribute error to each stage-specific mean and could weaken the estimated residual relationships or obscure individual carryover effects. Plant-level variances and covariances were not available for this analysis, so the contribution of sampling error cannot be separated from biological variation or model error. Whole-block resampling conditions on the observed subplot means and does not estimate this measurement component. For example, repeated non-destructive observations or retained individual measurements would be needed to test plant-level trajectories and propagate sampling uncertainty.

This distinction places multitemporal prediction studies in context. Reproductive-stage or multitemporal measurements can be highly predictive within specific sensor, irrigation or field domains [64,65], but stage proximity alone does not guarantee transfer. Additional satellite features beyond assimilated leaf area information have yielded marginal or unfavourable increases in hybrid soybean modelling [35], and multimodel evaluations report difficulty in reproducing in-season growth dynamics even when the final yield simulation is more satisfactory [24]. These findings reinforce the need to test the marginal information supplied by each data stream rather than infer cross-season values from biological plausibility or within-domain fit.

### 3.5. Robustness Across Modelling and Inferential Choices

The interpretation was retained across alternative algorithms, valid direct plant-scale representations, stage-only models, target transformations, and sowing-domain restrictions. The direct representation contrasts additionally addressed whether the compact and broader bases differed from one another rather than relying on separate comparisons with M0. Every predictor in the sensitivity set was a direct plant-scale measurement or a transformation of such measurements available within the stated V4–R6 information horizon. Together with the additive M0A analysis, these checks show that the result was not contingent on one trait encoding, one management benchmark, or one regularised estimator (Figure 9; Table S9).

Whole-block resampling retained all five sowing periods and the eight cultivar × seeding-rate subplots within every selected block. This preserved the complete-block structure, while the complementary sowing-period-stratified analysis examined sensitivity to a different main-plot resampling scheme. Both approaches condition on the observed seasons and fitted development models. Similar results across schemes demonstrate robustness to these resampling choices within the experiment; they do not supply additional year or site replication. With four blocks in an evaluation season, the narrow paired intervals must be interpreted in relation to that limited support rather than as evidence of precisely known future-season performance.

Reporting bootstrap threshold-attainment counts prevents their interpretation as posterior probabilities of an unknown parameter. The 2%, 5%, and 10% reference gains provide a scale for the observed changes, but none was calibrated to the cost of destructive sampling or the consequences of a forecasting decision. A consistent 0.5–1% improvement could have operational value where measurement costs are low or the decision benefits from small error reductions. Establishing that value would require repeated independent-environment validation and an explicit analysis of costs and decision loss [59,61]. The present estimates therefore support a small incremental change under the tested conditions, not a universal minimum improvement needed to justify phenotyping.

### 3.6. Scope and Implications

Reciprocal transfer across two seasons at one site supplies two direction-specific evaluations, not replication across a population of locations or climate regimes. Each evaluation holds one complete season outside model fitting, but both directions reuse the same pair of seasons and are not independent estimates of year-to-year variability. The bootstrap conditions on four complete blocks per evaluation season and on the fitted development model; it does not include development-season resampling, retuning, refitting, or variation among new sites and years. The conclusions therefore cannot establish a general upper bound on phenotype contribution, quantify how often a gain would occur across years, or determine performance in other germplasm, soils, and climates. Broader inference requires additional independent environments [50,52,61].

The management comparators restrict the target domain to the tested sowing periods, cultivars and seeding rates. Additional phenotyping is most likely to improve an incremental forecast when it measures crop-state differences not already encoded by management, those differences predict subsequent yield deviations, and the fitted relationship is represented in the development environments. These conditions can be tested by expanding the range of cultivars and stresses, improving the precision of subplot phenotypes, and recording sampling dates and crop development so that measurements and environmental exposure are aligned. Weather and soil–water information are plausible complementary predictors rather than demonstrated replacements for direct phenotypes in this experiment. Models combining these data streams should be evaluated prospectively at the intended forecast date across new seasons and sites, using only information then available [46,55,57,62].

The appropriate value criterion depends on the task. Destructive phenotyping can support physiological characterisation and the calibration of less costly non-destructive measurements [23,33]; breeding-screening utility would require validation in a broader set of lines than the two cultivars tested here. For operational forecasting, a useful data stream should improve the specified decision at an acceptable acquisition cost and lead time [59]. The present study provides a management-adjusted comparison that can be used for this evaluation, while its observed small increments do not justify a general recommendation to discontinue phenotyping or to prefer weather and soil measurements without a direct comparison.

## 4. Materials and Methods

### 4.1. Study Site and Environmental Conditions

Field experiments were conducted during the 2021/22 and 2022/23 soybean-growing seasons at the Embrapa Soybean experimental farm in Londrina, Paraná, southern Brazil (23°11′ S, 51°10′ W; 522 m above sea level). The regional climate is humid subtropical (Cfa in the Köppen classification), with warm, wet summers and no pronounced dry season. Rainfall and maximum and minimum air temperatures were recorded by an automatic weather station near the experimental area and summarised in 10-day intervals from September to April. Seasonal rainfall over this interval totalled 1105.3 mm in 2021/22 and 1283.7 mm in 2022/23, compared with a historical mean of 1112.5 mm. The mean maximum air temperature was 29.7 °C in 2021/22 and 27.0 °C in 2022/23, whereas the historical mean was 28.4 °C. Rainfall was relatively limited from November to February in 2021/22 and concentrated more strongly in March, whereas 2022/23 was wetter from December to February but dry in November.

The soil was a clayey dystroferric Red Latosol (Oxisol). Before experimental establishment, samples collected at 0–20 cm had a pH (CaCl_2_) of 5.9, an organic matter concentration of 29 g kg^−1^, an available P concentration of 26.15 mg dm^−3^, and exchangeable K, Ca, Mg, and Al concentrations of 0.34, 7.92, 2.13, and 0 cmolc dm^−3^, respectively. The potential acidity was 4.44 cmolc dm^−3^, the cation-exchange capacity was 14.74 cmolc dm^−3^, and the base saturation was 70.10%. The soil contained 714 g kg^−1^ clay, 98 g kg^−1^ silt, and 188 g kg^−1^ sand, and its field-capacity water content was 0.32 m^3^ m^−3^.

### 4.2. Experimental Design, Treatments, and Crop Management

Each season followed a split-plot randomised complete block design with four blocks. Five sowing dates were assigned to the main plots, and the eight combinations of two cultivars and four target seeding rates were assigned to subplots. The cultivars were BRS 1061 IPRO (relative maturity group 6.1; superearly) and DM 66i68 IPRO (relative maturity group 6.6; semiearly). The target rates were 200,000, 280,000, 360,000, and 440,000 viable seeds ha^−1^ and were corrected for the germination percentage of each seed lot. The design yielded 160 subplot observations per season and 320 across both seasons.

The sowing dates were 13 September, 12 October, 16 November, and 11 December 2021 and 15 January 2022 in the 2021/22 season and 29 September, 27 October, 30 November, and 26 December 2022 and 28 January 2023 in the 2022/23 season. January sowings complied with the sanitary calendar for Asian soybean rust in Paraná. Each experimental unit contained eight 12 m rows spaced 0.50 m apart (48 m^2^). The usable area was composed of six central rows that were 10 m in length (30 m^2^); 15 m^2^ was reserved for mechanical grain harvest, and 15 m^2^ was reserved for plant sampling.

The site had been managed under no-tillage for more than 10 years, with soybean and maize during spring–summer and wheat and black oat during autumn–winter. Soybean was sown into black-oat residue on every date during both seasons. Fertilisation followed soil test interpretation and regional recommendations. The germination and vigour of the seed lots were above 90%, and the seeds were treated with carbendazim and thiram and inoculated in the sowing furrow with *Bradyrhizobium japonicum* and *Bradyrhizobium diazoefficiens*. Soybean was irrigated from sowing until the V2 stage to achieve the target plant population and establish a uniform stand across all sowing dates in both growing seasons. Following establishment, all subplots were maintained under rainfed conditions from V3 until full maturity (R8). Irrigation was scheduled to replace 100% of estimated evapotranspiration losses and prevent water stress during establishment, based on daily weather monitoring and soil-water measurements. Water was applied uniformly across the experimental plots using self-propelled model HR G4-140 mobile equipment (Setorial Sistemas de Irrigação, Limeira, SP, Brazil) with boom-mounted sprinklers positioned approximately 1.0 m above the plant canopy. Weeds, insects, and diseases were managed according to regional recommendations and field monitoring.

### 4.3. Pre-Harvest Phenotyping and Grain Yield

The plants were assessed at V4, R2, R5, and R6 using the standard diagnostic criteria of the conventional soybean development scale [66]. At each stage, six plants were selected at random from the internal rows of the 15 m^2^ plant-sampling area within each subplot. Border plants, visibly damaged plants, and individuals not displaying the diagnostic morphology of the target stage were excluded from the eligible sampling population. The same six eligible plants supplied the direct architecture, stature, leaf-area, and biomass traits at that event; thus, the number of plants did not differ among the direct characteristics. Different plants were sampled at successive stages, so the temporal sequence was linked at the subplot level rather than representing repeated measurements of the same individuals. For each trait and stage, the arithmetic mean of the six plant-level observations constituted the subplot value.

The leaf area was measured with a LI-COR LI-3100 area meter (LI-COR Biosciences, Lincoln, NE, USA) and is expressed as dm^2^ plant^−1^. Before dissection, branches were defined as primary lateral stems and enumerated per plant; main-stem nodes were fully developed nodes counted along the principal axis; and plant height was measured from the soil surface to the main-stem apex. The architecture-assessment main-stem node count was the primary node trait. A separately recorded count obtained during stature assessment used the same anatomical definition and was evaluated only in the measurement-domain sensitivity analysis. The separated components were dried at 70 °C in a 210 L Marconi (Marconi Equipamentos para Laboratórios Ltda, Piracicaba, SP, Brazil) forced-air laboratory oven for approximately 72 h and, when necessary, for longer periods; drying and reweighing continued until successive determinations showed no detectable mass change. The component masses were summed as total dry matter and expressed as g plant^−1^.

Phenotyping was triggered by biological stage rather than by a common calendar date, a fixed number of days after sowing or a thermal-time threshold. Each sowing-period × cultivar cohort was monitored and sampled when the target diagnostic morphology was reached. Consequently, calendar dates, days after sowing and the interval from V4, R2, R5, or R6 to R8 varied with sowing period, cultivar and realised development; these timing quantities were not predictors in the present analysis.

At R8, the final plant population was determined by counting the plants in two 4 m row sections per subplot, totalling 8 m of rows. The yield components were measured for all the plants collected in two 1 m row sections and included pods plant^−1^, grains pod^−1^, pods m^−2^, grains m^−2^, and thousand-seed mass. Grain yield was determined by mechanically harvesting the net subplot area (15 m^2^) using a self-propelled combine designed for experimental plots (Nursery Master Elite; Wintersteiger, Ried im Innkreis, Austria). The harvested grain was cleaned and dried, and yield was expressed in kg ha^−1^ after adjustment to 13% moisture content. The grain yield was the sole maturity-stage outcome, and the predictive feature set comprised the direct V4–R6 measurements described above.

### 4.4. Analytical Population and Phenotypic Information Gates

Leaf area, dry matter, architecture, and height measurements were aligned one-to-one within each stage by season, sowing period, cultivar, seeding rate, and block. The grain yield was aligned by the same stage-free experimental key. The analytical population comprised 320 unique subplot contexts with complete primary predictors and outcomes; no primary numeric value was missing. The primary representation contained five directly measured plant-scale traits at each stage: leaf area per plant (dm^2^ plant^−1^), total dry matter per plant (g plant^−1^), branches per plant, main-stem node number obtained during the architecture assessment, and plant height (cm). The alternative main-stem node count obtained during the stature assessment was evaluated only as a measurement-domain sensitivity. Variable definitions, units, and representation membership are given in Table S1.

Predictor availability followed an ordered information sequence. M−1 was the development-season grand mean. M0 was the development-season mean of the corresponding sowing period × cultivar × seeding rate combination. M1 retained M0 as an unpenalised offset and added the five V4 traits; M2 added the five R2 traits; M3 added the five R5 traits; and M4 added the five R6 traits. The four cumulative phenotype gates therefore contained 5, 10, 15, and 20 numeric predictors. M4 was the terminal pre-harvest information gate. Predictor definitions were fixed from the measurement protocol and were not changed using independent-season performance.

### 4.5. Management Comparators, Phenotypic Correction, and Regularisation

Let s denote the development season, $s^{\prime }$ denote the independent season, and c(i) denote the sowing-period × cultivar × seeding-rate cell of observation i. The primary management prediction was

y^i,M0=μ^c(i),s=1nc,s∑j∈s:c(j)=c(i)yj.

Each development season contained 40 management combinations represented by four blocks; a leave-one-block-out inner fold therefore estimated each cell mean from three blocks. To assess whether inference depended on this cell-specific comparator, M0A was fitted by ordinary least squares with additive categorical main effects of sowing period, cultivar, and seeding rate:

y^i,M0A=γ^0,s+γ^E(i),s+γ^C(i),s+γ^D(i),s.

A ridge-regularised management-combination comparator (M0P) provided partial pooling across the same 40 cells. Unpenalised intercept and ridge-penalised cell indicators were used, with 25 λ values logarithmically spaced from 10^−4^ to 10^6^ selected by the same grouped inner cross-validation. The selected λ values were 0.0001 for development in 2021/22 and 0.0316228 for development in 2022/23. The near-zero first-direction penalty made M0P numerically identical to M0 at the reported precision, whereas the reciprocal fit applied modest shrinkage. Phenotype-augmented M0P models estimated the same type of elastic-net residual correction around the partially pooled offset (Figure 9; Tables S4, S9 and S10).

For the phenotype gate $M_{k}$, the model was fitted to the residual target rj,0=yj−y^j,M0, and its independent-season prediction was

y^i,Mk=y^i,M0+β^0,k+zi,kTβ^k,

where the predictor vector contains the standardised phenotypes available through gate k. The M0A and M0P robustness models use analogous residual targets and offsets. Management expectations remained fixed and unpenalised, whereas elastic-net regularisation acted only on the phenotypic correction [67]. The primary estimator minimised

$$\frac{1}{2n}\sum_{j=1}^{n}{\left(r_{j,0}-\beta_{0}-z_{j,k}^{T}\beta \right)}^{2}+\alpha \left[l\|\beta {\|}_{1}+\frac{1-l}{2}\|\beta {\|}_{2}^{2}\right],$$

where α is the overall penalty and ℓ is the L1 proportion [68]. The numerical predictors contained no missing values in the analytical population. A development-fold median-imputation step was nevertheless retained as a pipeline safeguard and was inactive in all reported fits. Predictors were subsequently standardised using the corresponding development-fold means and standard deviations.

### 4.6. Reciprocal Temporal Validation and Internal Tuning

Temporal transportability was evaluated in two reciprocal directions: models developed in 2021/22 were evaluated on all observations from 2022/23, and models developed in 2022/23 were evaluated on all observations from 2021/22. Management expectations, preprocessing quantities, hyperparameters, and coefficients were estimated exclusively within the development season. Combined reciprocal estimates were calculated only after the two independent-season prediction vectors were joined and did not replace direction-specific inference.

Pooled reciprocal statistics describe the concatenated prediction vectors; they are neither an average of the directional metrics nor an additional independent validation. Direction-specific results were therefore the basis for interpreting transfer.

Hyperparameters were selected within each development season by fourfold leave-one-block-out cross-validation. Each fold withheld one complete block, including its 5 sowing-period main plots and all 40 associated subplots. Management offsets, imputation medians, scaling quantities, and coefficients were re-estimated within every inner fold. This structure respected the experimental hierarchy and prevented observations from the same block from contributing simultaneously to fold-specific development and validation [50,54].

For elastic-net models on the original yield scale, the 20 penalty strengths were α_j_ = 10^−1 + 4.5j/19^, j = 0, …, 19, crossed with L1 proportions ℓ ∈ {0.05, 0.25, 0.50, 0.75, 0.95}, giving 100 candidate pairs. The pair minimising mean held-out-block RMSE was selected. Partial least-squares regression provided a correlated-predictor sensitivity analysis [69], with the component count selected from 1 to min(12, p), where p is the number of available phenotypic predictors. M0P used 25 ridge penalties from 10^−4^ to 10^6^. Each selected model was refitted to the full development season before independent-season evaluation. Selected penalties, L1 proportions, and component counts are reported separately for each direction and gate in Table S10 (Figure S5).

To distinguish within-season validation from independent-season transfer, an additional nested leave-one-block-out analysis was performed separately within each development season. Each of four outer folds withheld one complete block, including all five sowing periods and 40 subplots. For M1–M4, the remaining three blocks were used for a three-fold inner leave-one-block-out search over the original elastic-net grid. Management-combination means, imputation, standardisation, and model coefficients were estimated anew within every inner training split; the selected specification was then refitted to the three outer training blocks and evaluated on the untouched outer block. M−1 and M0 were re-estimated directly from those three blocks without tuning. Development-season RMSE was calculated from the 160 pooled outer-fold predictions. Original tuning-fold RMSE, which was used for hyperparameter selection, and apparent fitted RMSE are reported separately in Table S5. The nested estimates evaluate prediction of new blocks within the same season; they are not independent-season validation. Each outer fit used 120 subplots, whereas the final models used all 160 development-season subplots for independent-season transfer.

At M4, 20 phenotypic predictors were fitted to 160 development-season subplot records, a nominal ratio of eight records per predictor. Subplots were nested within 20 main plots and four blocks, and M0 additionally estimated 40 management-combination means. Original tuning fits used 120 records; nested outer and inner fits used 120 and 80, respectively. These counts do not imply independent records or 139 residual degrees of freedom. Effective complexity depends on shrinkage and predictor correlation; the conditional local sensitivity trace of the complete offset-plus-phenotype estimator is described in the Supplementary Methods.

### 4.7. Performance, Calibration, and Agreement

For observed values $y_{i}$, predictions y^i, independent-season mean ${\bar{y}}_{ind}$, and independent-season size n, performance was quantified as follows:

RMSE=1n∑i=1n(yi−y^i)2,MAE=1n∑i=1n|yi−y^i|,

nRMSE(%)=100RMSEy¯ind,Rpred2=1−∑i(yi−y^i)2∑i(yi−y¯ind)2.

The negative predictive R^2^ denotes a larger sum of squared errors than prediction by the observed independent-season mean. The calibration intercepts and slopes were obtained from the observed values regressed on the predictions. A positive mean bias denotes overprediction, and the standardised mean bias is divided by the sample standard deviation of the observed yield. Lin’s concordance correlation coefficient (CCC) was calculated as described above [70].

ρc=2syy^sy2+sy^2+(y¯−y^⃐)2.

The RMSE, MAE, and predictive R^2^ quantify the prediction error, whereas the calibration and CCC quantify the agreement [58,59]. The calibration slope and intercept were undefined for the constant M−1 predictor.

The paired-phenotype increment relative to a management comparator was

$$\Delta RMSE_{M_{k}:M0}(\%)=100\left(1-\frac{RMSE_{M_{k}}}{RMSE_{M0}}\right),$$

Positive paired gains denote lower independent-season RMSE after phenotype addition; MAE gains were defined analogously. The zero-correction skill score R^2^_corr_ = 1 − SSE(Mk)/SSE(M0) is zero when phenotype augmentation leaves squared error unchanged, positive when it reduces error and negative when it increases error. Pearson’s correlation describes alignment between the correction and the M0 residual but is not the correction skill itself.

ri,0=yi−y^i,M0,δi,k=y^i,Mk−y^i,M0,

$$R_{corr,k}^{2}=1-\frac{\sum_{i}(r_{i,0}-\delta_{i,k}{)}^{2}}{\sum_{i}r_{i,0}^{2}},$$

Spearman’s rank correlation between predicted and observed yield described ranking separately from calibration. Conditional within-season 95% percentile intervals for both Pearson and Spearman correlations used the same 2000 complete-block resamples as the performance analysis, with ranks recomputed within each resample and average ranks assigned to ties. Predictions remained fixed. Rank correlation is undefined for constant M−1 predictions. These statistics are predictive descriptors, not causal trait effects.

### 4.8. Design-Aware Bootstrap and Stage-Marginal Contrasts

Conditional within-season uncertainty was estimated using 2000 design-aware cluster-bootstrap resamples. The primary scheme resampled the four complete blocks with replacement within each independent season. Every selected block retained all five sowing-period main plots and all eight cultivar × seeding-rate subplots within each main plot. This scheme preserved a fixed representation of the five sowing periods, subplot composition, and dependence shared across main plots in the same block. Combined reciprocal resampling was stratified by transfer direction, with four complete blocks drawn independently from each evaluated season in every replicate. During independent-season resampling, development-season preprocessing quantities, selected hyperparameters, and fitted coefficients remained fixed.

A complementary sowing-period-stratified main-plot bootstrap resampled four complete block × sowing-period main plots with replacement within each of E1–E5 and retained their eight subplots. This scheme preserved the fixed number and proportion of observations from each sowing period while allowing the main plots to be resampled independently across periods. Agreement between the schemes was used as a robustness assessment. The reported intervals are conditional within-season intervals: they quantify the variation among observed independent-season blocks while the fitted development-season model is held fixed. They therefore do not include uncertainty from choosing a new development season, repeating hyperparameter selection or refitting, nor do they estimate between-year or between-site uncertainty.

Percentile 95% confidence intervals were calculated for absolute performance and row-matched paired contrasts. With four blocks, the primary bootstrap has 4^4^ = 256 ordered draws but only 35 unique block-multiplicity configurations when order is ignored; the 2000 Monte Carlo replicates repeatedly sample this finite support and are not 2000 independent experimental units. Exact enumeration of all 256 ordered configurations was therefore added as a threshold robustness check. For each direction, gate and comparison, counts at RMSE gains of at least 2%, 5%, and 10% describe resampled configurations and were not interpreted as probabilities of the unknown parameter. The 2%, 5%, and 10% gains were reference levels for describing effect magnitude.

Stage-marginal contrasts quantified the information added at M0 relative to M−1 and at each transition from M1–M0 through M4–M3. Positive values favour the richer or later gate. The same bootstrap draws were used for all contrasts within a direction. Simultaneous intervals for the family of four phenological increments were derived from a max statistic. For estimand g, let θ^g be the observed contrast, θ^gb* its bootstrap value in replicate b, and σ^g its empirical bootstrap standard error. The two-sided statistic was

Tb=maxg=1,…,4θ^gb*−θ^gσ^g,

and the simultaneous 95% interval was

θ^g±q0.95(Tb)σ^g.

One-sided simultaneous upper bounds used the 95th percentile of the maximum signed standardised deviation. The M0–M−1 management contrast was outside this four-increment family and retained its marginal percentile interval.

### 4.9. Cross-Season Predictor Domain Shift

The cross-season shift was quantified for each of the 20 primary stage–trait variables in both directions. For development-season values $x_{dev}$ and independent-season values $x_{ind}$, the SMD was

$$SMD=\frac{{\bar{x}}_{ind}-{\bar{x}}_{dev}}{s_{dev}},$$

The variance ratio was $s_{ind}^{2}/s_{dev}^{2}$, and the scaled first Wasserstein distance was $W_{1}(x_{dev},x_{ind})/s_{dev}$. Range extrapolation was the percentage of independent-season values below the development-season minimum or above the development-season maximum. These diagnostics characterised direction-specific support alongside prediction performance. Development-season Spearman correlations among the 20 primary predictors were ordered by average-linkage clustering of the dissimilarity 1−|ρ|, separately within seasons and without using grain yield (Figure S4).

### 4.10. Sensitivity and Statistical Analyses

Sensitivity analyses followed the same reciprocal temporal-transfer design. Model dependence was assessed by replacing elastic net with partial least squares and by fitting stage-only rather than cumulative phenotype blocks. Representation dependence was evaluated on an expanded direct plant-scale basis containing branch nodes and log-ratio biomass-allocation terms; an organ-component basis containing leaf, stem-plus-branch, and, at R5 and R6, reproductive dry matter; total-node and main-stem-node-fraction variables; alternative main-stem-node measures obtained during stature assessment; and observed internode length in place of plant height. The outcome was also modelled on the logarithmic scale with development-season smearing on return to kg ha^−1^, and the complete sowing domain was compared with E1–E4. Direct contrasts compared each valid alternative representation with the compact basis. All sensitivity representations comprised direct plant-scale measurements or transformations of them available within the V4–R6 information horizon.

Model fitting and grouped hyperparameter searches were deterministic. Bootstrap procedures used analysis-specific seeds derived deterministically from the resampling scheme and ordered analytical identifiers. Analyses were executed in Python version 3.13.9 (Python Software Foundation, Beaverton, OR, USA) with NumPy version 2.2.6, pandas version 2.3.3, SciPy version 1.16.3 and scikit-learn version 1.7.2.

## 5. Conclusions

Within this two-season experiment at one site, cumulative V4–R6 measurements of leaf area, dry matter, branching, main-stem nodes, and plant height changed independent-season RMSE by less than 1% relative to matching management-combination means. No stage addition improved prediction in both transfer directions, and alternative management comparators yielded similarly small increments. Management expectations themselves transferred asymmetrically: M0 improved on the development-season mean for 2021/22 → 2022/23 but not in the reciprocal direction, with different calibration and bias. These findings distinguish physiological trait relevance from the additional information supplied to a particular forecast. They do not establish a general bound on phenotype utility across environments. Wider environmental validation, more precise characterisation of subplot phenotypes, and aligned crop-state and environmental measurements are needed to identify when additional phenotyping provides a reproducible and operationally valuable improvement.

## Figures and Tables

**Table 1:** Observed grain yield distributions by season and sowing period.

Season	Sowing Period	Sowing Date	n	Mean ± SD, kg ha^−1^	Median [IQR], kg ha^−1^	Range, kg ha^−1^
2021/22	All	—	160	2752.4 ± 1286.3	3068.6 [1520.9–3810.2]	462.0–4862.4
2021/22	E1	13 September 2021	32	2390.6 ± 909.0	2304.3 [1520.9–3267.8]	1260.0–3960.0
2021/22	E2	12 October 2021	32	3874.5 ± 697.3	3843.0 [3235.2–4478.5]	2661.0–4862.4
2021/22	E3	16 November 2021	32	3931.9 ± 278.3	3934.2 [3726.5–4148.9]	3428.0–4530.0
2021/22	E4	11 December 2021	32	2737.1 ± 570.8	2624.6 [2395.8–3220.2]	1689.0–3753.6
2021/22	E5	15 January 2022	32	827.7 ± 275.4	759.0 [600.4–1051.0]	462.0–1399.3
2022/23	All	—	160	3152.9 ± 1654.9	3133.3 [1860.2–4767.8]	270.0–6144.0
2022/23	E1	29 September 2022	32	4894.4 ± 573.2	4907.2 [4603.9–5231.1]	3655.7–6144.0
2022/23	E2	27 October 2022	32	4906.4 ± 368.3	4897.5 [4668.7–5154.7]	4158.0–5579.0
2022/23	E3	30 November 2022	32	3108.1 ± 315.3	3133.3 [2921.7–3244.5]	2460.0–3687.5
2022/23	E4	26 December 2022	32	2050.2 ± 304.9	2005.0 [1860.2–2290.4]	1420.0–2680.0
2022/23	E5	28 January 2023	32	805.6 ± 375.7	754.5 [473.2–1093.8]	270.0–1558.6

The values are the observed plot-level grain yields adjusted to 13% moisture and are summarised for n = 32 observations per sowing period and n = 160 per season. SD, standard deviation; IQR, interquartile range.

**Table 2:** Within-development-season validation and independent-season predictive performance across the primary information sequence.

Transfer Direction	Gate	Nested RMSE kg ha^−1^	Independent RMSE kg ha^−1^ (95% CI)	Predictive R^2^ (95% CI)	Calibration Slope (95% CI)	Standardised Bias (95% CI)
2021/22 → 2022/23	M−1	1283.0	1697.7 (1636.7, 1756.5)	−0.059 (−0.066, −0.052)	—	−0.242 (−0.256, −0.228)
2021/22 → 2022/23	M0	244.7	1398.1 (1325.8, 1448.1)	0.282 (0.252, 0.315)	0.788 (0.759, 0.813)	−0.242 (−0.256, −0.228)
2021/22 → 2022/23	M1	242.6	1400.4 (1327.5, 1450.7)	0.279 (0.249, 0.313)	0.786 (0.756, 0.810)	−0.249 (−0.263, −0.236)
2021/22 → 2022/23	M2	245.7	1397.6 (1322.3, 1449.2)	0.282 (0.251, 0.318)	0.795 (0.764, 0.819)	−0.246 (−0.260, −0.233)
2021/22 → 2022/23	M3	247.7	1402.9 (1329.1, 1453.6)	0.277 (0.246, 0.311)	0.791 (0.760, 0.816)	−0.249 (−0.262, −0.235)
2021/22 → 2022/23	M4	246.7	1403.9 (1330.6, 1454.5)	0.276 (0.245, 0.310)	0.787 (0.756, 0.811)	−0.246 (−0.259, −0.232)
2022/23 → 2021/22	M−1	1650.1	1343.4 (1304.6, 1381.1)	−0.098 (−0.132, −0.071)	—	0.311 (0.266, 0.362)
2022/23 → 2021/22	M0	280.3	1394.3 (1377.9, 1416.4)	−0.182 (−0.273, −0.109)	0.474 (0.460, 0.488)	0.311 (0.266, 0.362)
2022/23 → 2021/22	M1	281.0	1395.8 (1378.3, 1418.6)	−0.185 (−0.277, −0.109)	0.472 (0.458, 0.487)	0.306 (0.262, 0.355)
2022/23 → 2021/22	M2	284.0	1400.3 (1381.8, 1425.2)	−0.192 (−0.282, −0.120)	0.466 (0.453, 0.480)	0.286 (0.241, 0.337)
2022/23 → 2021/22	M3	282.9	1401.2 (1382.6, 1423.6)	−0.194 (−0.291, −0.115)	0.473 (0.457, 0.489)	0.323 (0.285, 0.366)
2022/23 → 2021/22	M4	281.1	1404.2 (1387.1, 1426.5)	−0.199 (−0.293, −0.122)	0.470 (0.455, 0.485)	0.315 (0.276, 0.357)

M−1, development-season mean; M0, management-combination mean; M1–M4, cumulative V4, R2, R5, and R6 phenotype augmentation. Nested RMSE pools four outer held-out-block predictions per development season, with grouped inner tuning repeated for M1–M4. Parentheses give conditional within-season whole-block bootstrap 95% intervals for independent-season estimates (160 subplots within four blocks); models remained fixed during resampling. The nested outer fits used 120 subplots, and the final transferred fits used 160. The calibration slope is undefined for constant M−1 predictions. Standardised bias is mean prediction minus observation, divided by the observed independent-season SD. Extended performance and within-season errors are given in Tables S4 and S5.

**Table 3:** Paired incremental performance of cumulative phenotype gates relative to the management-combination mean.

Transfer Direction	Gate	RMSE Gain Over M0, % (95% CI)	MAE Gain Over M0, % (95% CI)	Delta Predictive R^2^ (95% CI)	Resamples ≥2/≥5/≥10%
2021/22 → 2022/23	M1	−0.168 (−0.200, −0.124)	0.282 (0.195, 0.370)	−0.002 (−0.003, −0.002)	0/0/0
2021/22 → 2022/23	M2	0.038 (−0.073, 0.247)	0.299 (0.041, 0.639)	0.001 (−0.001, 0.003)	0/0/0
2021/22 → 2022/23	M3	−0.345 (−0.405, −0.254)	−0.046 (−0.066, −0.015)	−0.005 (−0.006, −0.003)	0/0/0
2021/22 → 2022/23	M4	−0.419 (−0.449, −0.363)	−0.219 (−0.236, −0.198)	−0.006 (−0.007, −0.005)	0/0/0
2022/23 → 2021/22	M1	−0.104 (−0.252, 0.075)	−0.280 (−0.665, 0.082)	−0.002 (−0.006, 0.002)	0/0/0
2022/23 → 2021/22	M2	−0.427 (−0.630, −0.288)	−1.245 (−1.596, −1.032)	−0.010 (−0.015, −0.007)	0/0/0
2022/23 → 2021/22	M3	−0.495 (−0.707, −0.138)	0.264 (−0.219, 1.001)	−0.012 (−0.018, −0.003)	0/0/0
2022/23 → 2021/22	M4	−0.712 (−0.857, −0.531)	−0.433 (−0.765, 0.007)	−0.017 (−0.022, −0.012)	0/0/0
Pooled reciprocal predictions	M1	−0.136 (−0.214, −0.035)	0.002 (−0.184, 0.187)	−0.002 (−0.004, −0.001)	0/0/0
Pooled reciprocal predictions	M2	−0.194 (−0.309, −0.077)	−0.472 (−0.691, −0.269)	−0.003 (−0.005, −0.001)	0/0/0
Pooled reciprocal predictions	M3	−0.420 (−0.533, −0.242)	0.109 (−0.135, 0.499)	−0.007 (−0.010, −0.004)	0/0/0
Pooled reciprocal predictions	M4	−0.565 (−0.643, −0.469)	−0.326 (−0.496, −0.081)	−0.010 (−0.011, −0.008)	0/0/0

Positive values indicate a lower RMSE, lower MAE or higher predictive R^2^ after phenotype augmentation. Each comparison uses identical independent-season observations. The last column reports the numbers of 2000 whole-block bootstrap resamples attaining RMSE gains of at least 2%, 5%, and 10%, respectively.

**Table 4:** Management and successive phenological contrasts, with simultaneous intervals for the four stage additions.

Transfer Direction	Contrast	Information Added	RMSE Gain, % (95% CI)	One-Sided Upper 95%, %	Distance Below 10%, pp	Resamples ≥2/≥5/≥10%
2021/22 → 2022/23	M0 vs. M−1	Management combination	17.647 (15.918, 19.318)	—	—	2000/2000/2000
2022/23 → 2021/22	M0 vs. M−1	Management combination	−3.789 (−6.058, −1.722)	—	—	0/0/0
Pooled reciprocal predictions	M0 vs. M−1	Management combination	8.794 (7.664, 9.820)	—	—	2000/2000/18
2021/22 → 2022/23	M1 vs. M0	V4 conditional on management	−0.168 (−0.214, −0.123)	−0.123	10.123	0/0/0
2021/22 → 2022/23	M2 vs. M1	R2 conditional on V4	0.206 (0.046, 0.365)	0.365	9.635	0/0/0
2021/22 → 2022/23	M3 vs. M2	R5 conditional on V4 and R2	−0.382 (−0.500, −0.265)	−0.265	10.265	0/0/0
2021/22 → 2022/23	M4 vs. M3	R6 conditional on V4, R2 and R5	−0.075 (−0.120, −0.030)	−0.030	10.030	0/0/0
2022/23 → 2021/22	M1 vs. M0	V4 conditional on management	−0.104 (−0.304, 0.097)	0.091	9.909	0/0/0
2022/23 → 2021/22	M2 vs. M1	R2 conditional on V4	−0.323 (−0.511, −0.135)	−0.141	10.141	0/0/0
2022/23 → 2021/22	M3 vs. M2	R5 conditional on V4 and R2	−0.068 (−0.411, 0.276)	0.266	9.734	0/0/0
2022/23 → 2021/22	M4 vs. M3	R6 conditional on V4, R2 and R5	−0.215 (−0.406, −0.025)	−0.031	10.031	0/0/0
Pooled reciprocal predictions	M1 vs. M0	V4 conditional on management	−0.136 (−0.241, −0.031)	−0.034	10.034	0/0/0
Pooled reciprocal predictions	M2 vs. M1	R2 conditional on V4	−0.058 (−0.177, 0.060)	0.056	9.944	0/0/0
Pooled reciprocal predictions	M3 vs. M2	R5 conditional on V4 and R2	−0.225 (−0.408, −0.041)	−0.048	10.048	0/0/0
Pooled reciprocal predictions	M4 vs. M3	R6 conditional on V4, R2 and R5	−0.145 (−0.244, −0.046)	−0.049	10.049	0/0/0

For G(A:R) = 100 × [1 − RMSE(A)/RMSE(R)], positive values favour the augmented model A. The management contrast uses A = M0 and R = M−1; each phenological contrast uses the later gate as A and the preceding gate as R. The phenological intervals are simultaneous max-statistic intervals; M0−M−1 has a paired percentile interval. D10 = 10 − U95 is the distance between the simultaneous one-sided upper bound and the 10% reference gain. The last column reports resample counts at the 2%, 5%, and 10% thresholds.

## Data Availability

The original contributions presented in the study are included in the article and Supplementary Materials; further enquiries can be directed to the corresponding authors.

## Supplementary Materials

The following supporting information can be downloaded at: https://www.mdpi.com/article/10.3390/plants15182865/s1.
