# Supplementary material for: Sequential Soybean Phenotypes for Grain Yield Prediction in Reciprocal Two-Season Validation
Source: Plants (Basel). 2026 Sep 19;15(18):2865. doi: 10.3390/plants15182865 (PMC13610698; doi:10.3390/plants15182865)
Supplement: Supplementary file 1 [file plants-15-02865-s001.zip › plants-4567901-supplementary.pdf]

Supplementary Materials

Sequential Soybean Phenotypes for Grain Yield Prediction in Reciprocal Two-Season Validation

Renan Falcioni[1,2,*], José Salvador Simoneti Foloni[1,*], Luis Guilherme Teixeira Crusiol[1], Marcos Rafael Nanni[2] and José Renato Bouças Farias[1]

[1] National Soybean Research Center—Brazilian Agricultural Research Corporation, Rodovia Carlos João Strass, s/nº, Acesso Orlando Amaral, Distrito de Warta, Londrina 86085-981, PR, Brazil; salvador.foloni@embrapa.br (J.S.S.F.); luis.crusiol@colaborador.embrapa.br (L.G.T.C.); joserenato.farias@embrapa.br (J.R.B.F.)
[2] Graduate Program in Agronomy, State University of Maringá, Av. Colombo 5790, Maringá 87020-900, PR, Brazil; mrnanni@uem.br (M.R.N.);
* Correspondence: salvador.foloni@embrapa.br (J.S.S.F.); renanfalcioni@gmail.com or rfalcioni2@uem.br (R.F.); Tel.: +55-43-3371-6000 (J.S.S.F.); +55-44-3011-1359 (R.F.)

Supplementary Information

This document provides extended statistical methods, ten supplementary tables and eleven supplementary figures supporting the manuscript.

Supplementary Methods

Experimental population, phenotypic measurements and analytical unit

The analysis involved soybean field experiments conducted during the 2021/22 and 2022/23 crop seasons at a single site in Londrina, Paraná, Brazil. The experiments followed a split-plot randomised complete block design with four blocks. Five sowing periods (E1–E5) were assigned to the main plots, and the factorial combination of two cultivars and four target seeding rates (200,000, 280,000, 360,000 and 440,000 viable seeds ha^{-1}) was assigned to the subplots. The sowing dates were 13 September, 12 October, 16 November and 11 December 2021 and 15 January 2022 in the 2021/22 season and 29 September, 27 October, 30 November and 26 December 2022 and 28 January 2023 in the 2022/23 season.

Phenotypic measurements were obtained at V4, R2, R5 and R6 using the conventional soybean development scale. At each stage, six plants were selected at random from the internal rows of the 15 m^2 sampling area within each subplot; border plants, visibly damaged plants and plants not displaying the diagnostic morphology of the target stage were excluded from eligibility. The same six eligible plants supplied the architecture, stature, leaf area and biomass traits at that event; thus, the number of plants did not differ among the direct characteristics. Successive stages were represented by different destructively sampled plants rather than repeated measurements of the same individuals. For every trait and stage, the subplot value was the arithmetic mean of the six plant-level observations. Sampling was triggered by target-stage morphology within each sowing period × cultivar cohort, not by a common calendar date, fixed days after sowing or thermal-time threshold; the interval from each phenotyping event to R8 therefore followed realised development.

Cumulative gates concatenate means from successive samples within the same subplot. They do not follow individual plants through time. Plant-level variances and covariances were not available for this analysis; the whole-block intervals condition on the observed subplot means and do not propagate the sampling variance of the six-plant means.

The leaf area was measured with a LI-COR LI-3100 area meter (LI-COR Biosciences, Lincoln, NE, USA). Before dissection, primary lateral stems were counted as branches, fully developed main-stem nodes were counted along the principal axis, and plant height was measured from the soil surface to the main-stem apex. The primary node trait came from the architecture assessment; a separate stature-assessment count used the same anatomical definition and was entered only in the sensitivity analysis. The separated components were dried at 70 °C in a 210-L Marconi forced-air laboratory oven for approximately 72 h and, when necessary, for longer until the mass did not change. The analytical unit was season × sowing period × cultivar × seeding rate × block observation, yielding 160 observations per season and 320 observations in total. The grain yield at R8, adjusted to 13% moisture (kg ha^{-1}), was the sole outcome; the predictor window ended at R6.

Reciprocal whole-season validation and information gates

Two reciprocal temporal transfers were evaluated: model development on 2021/22, which was followed by an independent whole-season assessment on 2022/23, and model development on 2022/23, which was followed by an independent whole-season assessment on 2021/22. Management-comparator estimation, standardisation, hyperparameter selection and model fitting were performed exclusively with development-season observations. The primary analytical matrix had no missing numeric predictors or outcomes; a development-fold median-imputation safeguard was retained in the pipeline but was inactive in every reported fit. The two out-of-season prediction vectors were combined only after both direction-specific analyses had been completed; pooled reciprocal estimates were secondary to the direction-specific results.

Pooled reciprocal statistics are descriptive summaries of concatenated predictions, not averages of transfer success or additional independent validation; directional calibration and error remain the primary evidence.

The information sequence began with M−1, the development-season overall mean grain yield. M0 was the development-season mean for the matching sowing-period × cultivar × seeding-rate combination. Every management combination occurred in both seasons; M0 was therefore estimated from four development-season block observations and applied to the corresponding combination in the independent season. M1 retained the management prediction as a fixed offset and added V4 phenotypes; M2 added cumulative V4 and R2 phenotypes; M3 added V4, R2 and R5 phenotypes; and M4 added all measurements through R6. M4 was the terminal information gate defined for the analysis, irrespective of independent-season performance.

Two less saturated management comparators assessed whether conclusions depended on the four-observation management–combination mean. M0A was an ordinary least-squares model containing additive categorical main effects of the sowing period, cultivar and target seeding rate. M0P represents the 40 sowing-period × cultivar × seeding-rate cells by indicator variables, and ridge shrinkage is applied toward the overall development-season mean. Twenty-five λ values logarithmically spaced from 10^{-4} to 10^{6} were selected by grouped inner cross-validation. The selected λ values were 0.0001 for 2021/22 → 2022/23 and 0.0316228 for 2022/23 → 2021/22; the near-zero first-direction penalty made M0P numerically identical to M0 at the reported precision. For each

comparator, the corresponding M1–M4 models estimated an elastic-net phenotypic correction around that comparator.

Plant-scale predictor representations

The primary compact representation contained five directly measured variables at each stage: leaf area per plant, total dry matter per plant, branches per plant, main-stem node number from the plant-architecture assessment and plant height. The cumulative M1–M4 models therefore contained 5, 10, 15 and 20 numeric phenotypic predictors, respectively.

Five alternative plant-scale bases were evaluated for sensitivity to biological detail, algebraic structure and measurement definition without the introduction of area-converted quantities:

The expanded plant representation added branch nodes and the natural logarithm of the leaf-to-stem-plus-branch dry matter ratio at every stage, with the reproductive-to-vegetative dry matter log ratio additionally included at R5 and R6. The organ-component representation replaced total dry matter and allocation ratios with leaf and stem-plus-branch dry matter and additionally included reproductive dry matter at R5 and R6; branch nodes were retained. The total-node representation used total nodes and the main-stem-node fraction in place of architecture-assessment node counts. The stature-assessment representation substituted the separately recorded main-stem node count, whereas the internode representation substituted observed mean internode length for height while retaining architecture-assessment main-stem and branch node counts.

For the allocation coordinates,

$$L_{leaf:stem} = \log\left(\frac{M_{leaf}}{M_{stem+branch}}\right)$$

and

$$L_{reproductive:vegetative} = \log\left(\frac{M_{reproductive}}{M_{leaf} + M_{stem+branch}}\right).$$

All logarithms were natural logarithms. Exact totals and their components, duplicated node measures, and algebraically dependent height–node–internode combinations were not entered together in one model. Table S1 identifies the membership of every measured or derived predictor in each valid representation.

Offset models and preprocessing

For phenotype-augmented models on the original yield scale, the selected management prediction was retained as a fixed, unpenalised offset. The response supplied to the phenotype model was the residual from that offset, so regularisation estimated only the incremental phenotypic correction:

$$y_i = \hat{y}_{i,M} + f(x_i) + \varepsilon_i,$$

where the offset denotes the development-season prediction from M0, M0A or M0P and the second term is the phenotype-based correction. No numeric predictor was missing in the analytical population. The modelling pipeline nevertheless retained a development-fold median-imputation step as a safeguard, followed by standardisation using the corresponding development-fold means and standard deviations; consequently, no observed value was imputed in the reported analyses. The management comparator, preprocessing quantities and phenotype model were re-estimated within every inner fold.

Elastic-net regression was the primary phenotype model. For standardised predictor matrix X, residual target r, intercept β_0, regularisation strength α and L1 proportion ℓ, the fitted coefficients minimised

$$\frac{1}{2n}\|r - \beta_0\mathbf{1} - X\boldsymbol{\beta}\|_2^2 + \alpha\left[\ell\|\boldsymbol{\beta}\|_1 + \frac{1-\ell}{2}\|\boldsymbol{\beta}\|_2^2\right].$$

Partial least-squares (PLS) regression was used as a correlated-predictor sensitivity model and was fitted to the same standardised residual target. Coefficients from both algorithms were interpreted as conditional predictive parameters rather than causal effects or universal measures of biological importance.

Grouped inner cross-validation and hyperparameter selection

Hyperparameters were selected using four leave-one-block-out folds within the development season. In each fold, one complete block formed the assessment fold, comprising all five sowing-period main plots and 40 subplot observations; the remaining three blocks comprised 120 observations. This structure retained each block × sowing-period main plot intact and preserved all five sowing periods in every fold. The management comparator, imputation, standardisation and phenotype models were estimated from the corresponding three-block development subset.

For the elastic net on the original yield scale, 20 values of α followed $\alpha_j = 10^{-1+4.5j/19}$, j = 0, …, 19 and crossed with L1 proportions of 0.05, 0.25, 0.50, 0.75 and 0.95. For the log-yield elastic net, 17 α values were logarithmically spaced from 10^{-4} to 1 with the same L1 proportions. The PLS models considered one-to-min(12, p) components, where p was the number of phenotypic predictors at the relevant gate. M0P considered 25 ridge penalties from 10^{-4} to 10^{6}. The combination with the smallest mean inner-fold RMSE was refitted to the complete development season and applied once to the independent season.

The log-yield sensitivity was fitted to $\log(y)$, using the development-season management-combination mean of the log yield as its fixed offset. The predictions were returned to kg ha^{-1} using a Duan smearing factor estimated exclusively from the complete development season,

$$\hat{y} = \exp(\hat{\log y}) \times \frac{1}{n_{dev}} \sum_{i=1}^{n_{dev}} \exp[\log(y_i) - \hat{\log(y_i)}].$$

All target-scale sensitivities were evaluated on the original yield scale during the independent season.

To distinguish within-season validation from independent-season transfer, an additional nested leave-one-block-out analysis was performed separately within each development season. Each of four outer folds withheld one complete block, including all five sowing periods and 40 subplots. For M1–M4, the remaining three blocks were used for a three-fold inner leave-one-block-out search over the original elastic-net grid. Management-combination means, imputation, standardisation and model coefficients were estimated anew within every inner training split; the selected specification was then refitted to the three outer training blocks and evaluated on the untouched outer block. M−1 and M0 were re-estimated directly from those three blocks without tuning. Development-season RMSE was calculated from the 160 pooled outer-fold predictions. Original tuning-fold RMSE, which was used for hyperparameter selection, and apparent fitted RMSE are reported separately. The nested estimates evaluate prediction of new blocks within the same season; they are not independent-season validation. Each outer fit used 120 subplots, whereas the final models used all 160 development-season subplots for independent-season transfer.

Conditional fitting complexity

At M4, 20 phenotype predictors were fitted using 160 development-season subplot records (eight records per predictor). The records were nested within four blocks and 20 main plots; the management offset additionally estimated 40 cell means. All 20 M4 phenotype coefficients were non-zero in both development seasons. Original tuning fits used 120 records; nested outer and inner fits used 120 and 80 records, respectively. Neither nominal record count nor parameter count defines an effective independent sample size or unpenalised residual degrees of freedom.

Let P be the projection matrix for the 40 management-cell means and Z_A the centred, standardised predictor matrix restricted to active coefficients. Conditional on fixed predictors, selected penalties, the active coefficient set and coefficient signs, the local derivative of fitted values with respect to development outcomes is:
$H = P + Z_A [Z_A'Z_A + n\alpha(1 - \ell)I]^{-1} Z_A'(I - P)$.

Here, n is the development sample size, α is the penalty strength, ℓ is the L1 proportion and I is the identity matrix of the required dimension. Local complexity is tr(H), and its incremental phenotype component is tr(H) − 40. At M4, the incremental values were 1.175 and 4.259 for development in 2021/22 and 2022/23, giving total values of 41.175 and 44.259. The intercept is already represented in P; the residual target has mean zero. These are conditional local fitting-complexity descriptors, not inferential residual degrees of freedom or effective sample sizes, and they exclude hyperparameter-selection uncertainty.

Predictive-performance estimands and paired comparisons

For the observed grain yield y_i, prediction $\hat{y}_i$, independent-season mean $\overline{y}$, independent-season standard deviation s_y and sample size n, the performance was quantified as follows:

$$RMSE = \sqrt{\frac{1}{n}\sum_{i=1}^{n}(y_i - \hat{y}_i)^2}, MAE = \frac{1}{n}\sum_{i=1}^{n}|y_i - \hat{y}_i|,$$

$$R^2_{pred} = 1 - \frac{\sum_i (y_i - \hat{y}_i)^2}{\sum_i (y_i - \overline{y})^2}, nRMSE(\%) = 100 \times \frac{RMSE}{\overline{y}}.$$

Lin's concordance correlation coefficient was

$$CCC = \frac{2s_{y\hat{y}}}{s_y^2 + s_{\hat{y}}^2 + (\overline{y} - \overline{\hat{y}})^2}.$$

The calibration intercept and slope were estimated from $y_i = a + b\hat{y}_i + \varepsilon_i$. The mean bias was $\overline{(\hat{y} - y)}$; thus, positive values denoted overprediction, and the standardised mean bias was $\overline{(\hat{y} - y)}/s_y$. The calibration parameters were undefined for a constant prediction.

Pearson's correlation quantified alignment between the phenotypic correction, ŷMk − ŷM0, and the observed yield residual, y − ŷM0. Spearman's rank correlation between predicted and observed yield described ranking independently of absolute calibration. For each statistic, conditional within-season 95% percentile intervals used the same 2000 complete-block resamples as the performance analysis, with all five sowing periods and their subplots retained in every sampled block. Ranks were recomputed within each resample, using average ranks for ties. Predictions and fitted models remained fixed. These intervals describe uncertainty among the four observed blocks in the evaluated season and do not estimate between-year or between-site uncertainty. Rank correlation is undefined for the constant M−1 predictions.

For an error metric Q, where Q is the RMSE or mean absolute error (MAE), the paired gain of an added-information model A relative to its row-matched reference R is

$$G_Q(A:R) = 100\left[1 - \frac{Q(A)}{Q(R)}\right].$$

Positive values indicate lower independent-season errors after information is added. For direct cumulative comparisons, M0 was used as the reference for M1–M4. The sequential comparisons were M0 relative to M−1, M1 relative to M0, M2 relative to M1, and M3 relative to M2 and M4 relative to M3. M0A and M0P, and the phenotype-augmented models fitted around each comparator, were evaluated analogously.

Alternative phenotype representations were also compared directly with the compact representation at the same information gate, transfer direction, algorithm,

management comparator, sowing domain and target scale. In these contrasts, a positive gain indicated lower error for the alternative representation than for the compact representation. Direct representation contrasts prevent conclusions from relying solely on separate comparisons of each representation with M0.

Conditional resampling and RMSE-gain reference levels

Conditional within-season uncertainty was estimated from 2000 paired bootstrap resamples under the two schemes. The primary scheme resampled the four complete independent-season blocks with replacement; every selected block retained all five sowing-period main plots and all eight cultivar × seeding-rate subplots within each main plot. The complementary scheme was stratified by the sowing period: within each E1–E5 stratum, four block × sowing-period main plots were resampled with replacement, and all eight subplots in a selected main plot were retained. Both schemes preserved the fixed sowing-period representation and relevant subplot dependence. During independent-season resampling, development-season preprocessing quantities, selected hyperparameters and fitted coefficients remained fixed.

Model and reference predictions were paired within every resample. For pooled reciprocal estimates, resampling was performed separately within the two transfer directions before sufficient statistics were combined. Primary intervals were conditional within-season 95% percentile intervals: they quantify variation among observed independent-season blocks while holding the fitted development-season model fixed and do not include development-season refitting or tuning uncertainty, between-year uncertainty or between-site uncertainty. With four blocks, the whole-block scheme has 4^4 = 256 ordered draws and 35 unique block-multiplicity configurations; the 2000 Monte Carlo replicates repeatedly sample this finite support and are not 2000 independent units. Exact enumeration of all 256 ordered configurations confirmed that no primary gate attained a 2%, 5% or 10% RMSE gain in either direction.

The four phenological increments—M1−M0, M2−M1, M3−M2 and M4−M3—formed one simultaneous family within each transfer direction and for pooled reciprocal predictions. The same bootstrap draws were used for all four contrasts. Each centred bootstrap estimate was divided by its bootstrap standard error. The 95th percentile of the replicatewise maximum absolute standardised deviation supplied the two-sided max-statistic critical value, whereas the 95th percentile of the replicatewise maximum signed deviation supplied the simultaneous one-sided upper critical value. Simultaneous intervals were the point estimate plus or minus the two-sided critical value multiplied by its bootstrap standard error. The M0−M−1 contrast was outside this four-contrast family and received a marginal interval.

Support at three illustrative RMSE-gain reference levels was summarised as the number of bootstrap resamples attaining at least 2%, 5% and 10% improvement. For threshold t,

$$N_{\geq t} = \sum_{b=1}^{2000} \mathbb{I}\{ G_{RMSE}^{(b)} \geq t\}, t \in \{2,5,10\}.$$

These finite-resample counts and exact-enumeration counts were reported without extrapolation to parameter probabilities. The distance below a reference gain was

$$D_t = t - U_{95},$$

where U_{95} is the relevant marginal or simultaneous one-sided upper 95% bound. A positive D_t indicated that the interval's upper bound remained below the corresponding reference gain; it did not imply that the true gain was exactly zero.

The 2%, 5% and 10% reference gains were not calibrated to phenotyping costs, yield prices or decision loss. Continuous paired estimates therefore provide the primary description of effect magnitude; the counts do not define an economic minimum benefit or an upper bound for future years and sites.

Directional domain shift and predictor redundancy

Domain shift was quantified separately for each of the 20 compact trait–stage predictors and each transfer direction. Let $\overline{x}_{dev}$, $\overline{x}_{ind}$, s_{dev} and s_{ind} denote the development- and independent-season means and standard deviations. The signed standardised mean difference was

$$SMD = \frac{\overline{x}_{ind} - \overline{x}_{dev}}{s_{dev}},$$

and the variance ratio was s_{ind}^2/s_{dev}^2. The first Wasserstein distance between the empirical development and independent-season distributions was divided by s_{dev}. Range extrapolation was the number and percentage of independent-season observations below the development-season minimum or above the development-season maximum. Stage-level summaries included the median absolute SMD and the mean outside-range percentage across the five compact traits. These measures characterised direction-specific distributional support and did not determine model inclusion.

Development-season redundancy was described using Spearman rank correlations among the 20 compact trait–stage variables. Variables were ordered separately within a season by average-linkage hierarchical clustering of the dissimilarity $1 - |\rho|$. Each matrix was constructed independently within one potential development season to characterise response-independent predictor redundancy; the representation was associational rather than causal.

Sensitivity analyses and subgroup summaries

The complete specification set contained the primary compact cumulative elastic-net model on the original yield scale and eleven alternatives: compact cumulative PLS; compact stage-only elastic net; expanded plant cumulative elastic net; organ-component cumulative elastic net; total-node cumulative elastic net; stature-assessment node cumulative elastic net; internode cumulative elastic net; compact cumulative elastic net restricted to E1–E4; compact cumulative elastic net fitted to log yield with development-only smearing retransformation; compact cumulative elastic net around M0A; and compact cumulative elastic net around M0P. Every phenotype-augmented specification was compared with its matched management comparator on identical independent-season observations.

The performance of the primary M0–M4 sequence was additionally summarised by sowing period, cultivar and seeding rate. Within each transfer direction, the subgroup sample sizes were n = 32 per sowing period, n = 80 per cultivar and n = 40 per seeding rate level. Model selection remained at the complete development-season level, and subgroup summaries were interpreted descriptively.

Model fitting and grouped hyperparameter searches were deterministic. Bootstrap procedures used analysis-specific seeds derived deterministically from the resampling scheme and ordered analytical identifiers. Analyses were executed in Python version 3.13.9 (Python Software Foundation, Beaverton, OR, USA) with NumPy 2.2.6, pandas 2.3.3, SciPy 1.16.3 and scikit-learn 1.7.2.

Supplementary Tables

Table S1. Traits, units and predictor membership in the six plant-scale representations.

Trait	Stages	Unit	C	E	O	T	S	I
Leaf area	V4–R6	dm^2 $plant^{-1}$	✓	✓	✓	✓	✓	✓
Total dry matter	V4–R6	g $plant^{-1}$	✓	✓	—	✓	✓	✓
Leaf dry matter	V4–R6	g $plant^{-1}$	—	—	✓	—	—	—
Stem + branch dry matter	V4–R6	g $plant^{-1}$	—	—	✓	—	—	—

Trait	Stages	Unit	C	E	O	T	S	I
Reproductive dry matter	R5, R6	g plant^{-1}	—	—	✓	—	—	—
Branches	V4–R6	plant^{-1}	✓	✓	✓	✓	✓	✓
Main-stem nodes (architecture)	V4–R6	plant^{-1}	✓	✓	✓	—	—	✓
Branch nodes	V4–R6	plant^{-1}	—	✓	✓	—	—	✓
Total nodes	V4–R6	plant^{-1}	—	—	—	✓	—	—
Main-stem-node fraction	V4–R6	ratio	—	—	—	✓	—	—
Plant height	V4–R6	cm	✓	✓	✓	✓	✓	—
Main-stem nodes (stature)	V4–R6	plant^{-1}	—	—	—	—	✓	—
Mean main-stem internode length	V4	cm	—	—	—	—	—	✓
Mean internode length	R2, R5, R6	cm	—	—	—	—	—	✓
ln[leaf/(stem + branch) dry matter]	V4–R6	log ratio	—	✓	—	—	—	—
ln(reproductive/vegetative dry matter)	R5, R6	log ratio	—	✓	—	—	—	—

✓, included; —, not included. C, compact; E, expanded; O, organ components; T, total-node basis; S, stature-assessment nodes; I, internode basis. Stage groups indicate inclusion at each listed stage when it is available to the cumulative gate. Architecture and stature counts use the same anatomical node definition but represent separately recorded measurements. Leaf and stem-plus-branch mass form the vegetative denominator; reproductive mass is pod-plus-grain mass. All logarithms are natural. Ratios and node fractions are dimensionless. Stage-specific variables are concatenated, not averaged across stages.

Table S2. Observed descriptive statistics for the compact phenotypic traits.

Trait	Stage	Unit	Season	n	Mean ± SD	Median [IQR]	Range
Leaf area	V4	dm^2 plant^{-1}	2021/22	160	2.457 ± 1.383	2.115 [1.325–3.121]	0.720–6.773
Leaf area	V4	dm^2 plant^{-1}	2022/23	160	1.837 ± 0.478	1.782 [1.523–2.152]	1.024–3.362
Total dry matter	V4	g plant^{-1}	2021/22	160	1.981 ± 1.032	1.678 [1.256–2.205]	0.757–5.350
Total dry matter	V4	g plant^{-1}	2022/23	160	1.118 ± 0.247	1.127 [0.934–1.266]	0.670–1.832
Branches	V4	count plant^{-1}	2021/22	160	1.296 ± 0.936	1.167 [0.487–2.000]	0.000–3.833
Branches	V4	count plant^{-1}	2022/23	160	0.764 ± 0.710	0.600 [0.325–0.900]	0.000–3.800
Main-stem nodes	V4	count plant^{-1}	2021/22	160	5.921 ± 0.540	5.920 [5.537–6.300]	4.650–7.050
Main-stem nodes	V4	count plant^{-1}	2022/23	160	5.857 ± 0.454	6.000 [5.400–6.200]	5.000–6.700
Plant height	V4	cm	2021/22	160	16.593 ± 2.465	16.250 [15.075–18.000]	11.800–23.000
Plant height	V4	cm	2022/23	160	13.587 ± 1.604	13.333 [12.500–14.667]	10.333–18.000
Leaf area	R2	dm^2 plant^{-1}	2021/22	160	8.390 ± 7.231	6.058 [2.777–10.816]	1.683–30.621
Leaf area	R2	dm^2 plant^{-1}	2022/23	160	6.964 ± 4.314	5.315 [3.747–10.098]	1.637–20.114
Total dry matter	R2	g plant^{-1}	2021/22	160	6.523 ± 5.373	4.556 [2.671–7.537]	1.200–24.236
Total dry matter	R2	g plant^{-1}	2022/23	160	5.139 ± 3.342	4.109 [2.604–7.021]	1.175–15.547
Branches	R2	count plant^{-1}	2021/22	160	2.810 ± 1.330	2.833 [1.833–3.667]	0.000–6.333
Branches	R2	count plant^{-1}	2022/23	160	3.358 ± 2.224	3.633 [1.050–5.333]	0.000–7.600
Main-stem nodes	R2	count plant^{-1}	2021/22	160	11.509 ± 2.155	11.317 [10.000–12.333]	7.400–16.333
Main-stem nodes	R2	count plant^{-1}	2022/23	160	9.918 ± 1.807	9.667 [8.500–11.167]	6.167–14.667
Plant height	R2	cm	2021/22	160	29.930 ± 10.202	28.583 [21.500–32.500]	16.000–55.500
Plant height	R2	cm	2022/23	160	32.424 ± 11.154	27.750 [24.333–37.708]	18.600–64.500
Leaf area	R5	dm^2 plant^{-1}	2021/22	160	14.338 ± 7.448	13.620 [7.973–19.778]	3.036–36.060

Trait	Stage	Unit	Season	n	Mean ± SD	Median [IQR]	Range
Leaf area	R5	dm² plant⁻¹	2022/23	160	12.891 ± 6.948	12.315 [9.077–17.596]	1.802–31.386
Total dry matter	R5	g plant⁻¹	2021/22	160	19.620 ± 9.615	20.102 [10.453–26.159]	5.842–50.458
Total dry matter	R5	g plant⁻¹	2022/23	160	13.207 ± 6.538	13.009 [9.163–17.462]	2.828–33.844
Branches	R5	count plant⁻¹	2021/22	160	3.439 ± 1.246	3.200 [2.500–4.211]	0.700–6.833
Branches	R5	count plant⁻¹	2022/23	160	3.231 ± 1.785	3.167 [1.900–4.500]	0.200–7.800
Main-stem nodes	R5	count plant⁻¹	2021/22	160	16.715 ± 2.746	17.000 [15.000–18.542]	11.092–23.500
Main-stem nodes	R5	count plant⁻¹	2022/23	160	13.867 ± 2.473	13.833 [12.125–15.333]	9.167–19.833
Plant height	R5	cm	2021/22	160	60.381 ± 20.083	61.267 [42.475–74.542]	26.800–110.200
Plant height	R5	cm	2022/23	160	57.831 ± 19.161	54.917 [47.125–66.042]	27.167–105.400
Leaf area	R6	dm² plant⁻¹	2021/22	160	11.330 ± 5.677	11.490 [6.323–15.379]	2.203–27.748
Leaf area	R6	dm² plant⁻¹	2022/23	160	7.568 ± 6.033	4.571 [2.828–11.696]	1.007–23.657
Total dry matter	R6	g plant⁻¹	2021/22	160	28.952 ± 12.896	27.703 [20.291–35.956]	8.502–72.670
Total dry matter	R6	g plant⁻¹	2022/23	160	22.526 ± 12.144	22.413 [13.684–30.621]	4.240–51.531
Branches	R6	count plant⁻¹	2021/22	160	3.359 ± 1.193	3.333 [2.500–4.175]	0.700–6.500
Branches	R6	count plant⁻¹	2022/23	160	2.921 ± 1.597	2.817 [1.767–4.025]	0.200–6.900
Main-stem nodes	R6	count plant⁻¹	2021/22	160	17.697 ± 2.623	17.833 [15.458–19.425]	12.300–24.000
Main-stem nodes	R6	count plant⁻¹	2022/23	160	15.385 ± 3.349	15.333 [13.259–18.000]	8.667–22.333
Plant height	R6	cm	2021/22	160	63.234 ± 21.589	62.000 [45.400–79.875]	30.667–112.500
Plant height	R6	cm	2022/23	160	65.639 ± 23.634	61.500 [52.833–88.042]	23.600–111.167

Values are unstandardised observations in the stated scientific units from n = 160 plots per season and stage. SD, standard deviation; IQR, interquartile range.

Table S3. Directional distribution shift of the compact predictors.

Direction	Stage	Trait	Development mean ± SD	Independent-season mean ± SD	SMD	Variance ratio	Scaled W1	Outside range, %
2021/22 → 2022/23	V4	Leaf area	2.457 ± 1.383	1.837 ± 0.478	−0.448	0.119	0.542	0.0
2021/22 → 2022/23	V4	Total dry matter	1.981 ± 1.032	1.118 ± 0.247	−0.836	0.057	0.836	9.4
2021/22 → 2022/23	V4	Branches	1.296 ± 0.936	0.764 ± 0.710	−0.568	0.575	0.575	0.0
2021/22 → 2022/23	V4	Main-stem nodes	5.921 ± 0.540	5.857 ± 0.454	−0.119	0.705	0.214	0.0
2021/22 → 2022/23	V4	Plant height	16.593 ± 2.465	13.587 ± 1.604	−1.22	0.424	1.22	11.2

Direction	Stage	Trait	Development mean ± SD	Independent-season mean ± SD	SMD	Variance ratio	Scaled W1	Outside range, %
2021/22 → 2022/23	R2	Leaf area	8.390 ± 7.231	6.964 ± 4.314	−0.197	0.356	0.278	0.6
2021/22 → 2022/23	R2	Total dry matter	6.523 ± 5.373	5.139 ± 3.342	−0.258	0.387	0.269	0.6
2021/22 → 2022/23	R2	Branches	2.810 ± 1.330	3.358 ± 2.224	0.412	2.798	0.765	6.9
2021/22 → 2022/23	R2	Main-stem nodes	11.509 ± 2.155	9.918 ± 1.807	−0.739	0.703	0.739	5.0
2021/22 → 2022/23	R2	Plant height	29.930 ± 10.202	32.424 ± 11.154	0.244	1.195	0.256	3.8
2021/22 → 2022/23	R5	Leaf area	14.338 ± 7.448	12.891 ± 6.948	−0.194	0.87	0.219	12.5
2021/22 → 2022/23	R5	Total dry matter	19.620 ± 9.615	13.207 ± 6.538	−0.667	0.462	0.667	18.8
2021/22 → 2022/23	R5	Branches	3.439 ± 1.246	3.231 ± 1.785	−0.167	2.054	0.379	10.6
2021/22 → 2022/23	R5	Main-stem nodes	16.715 ± 2.746	13.867 ± 2.473	−1.037	0.81	1.037	15.6
2021/22 → 2022/23	R5	Plant height	60.381 ± 20.083	57.831 ± 19.161	−0.127	0.91	0.207	0.0
2021/22 → 2022/23	R6	Leaf area	11.330 ± 5.677	7.568 ± 6.033	−0.663	1.129	0.663	19.4
2021/22 → 2022/23	R6	Total dry matter	28.952 ± 12.896	22.526 ± 12.144	−0.498	0.887	0.498	18.8
2021/22 → 2022/23	R6	Branches	3.359 ± 1.193	2.921 ± 1.597	−0.368	1.793	0.443	8.1
2021/22 → 2022/23	R6	Main-stem nodes	17.697 ± 2.623	15.385 ± 3.349	−0.881	1.63	0.881	21.9
2021/22 → 2022/23	R6	Plant height	63.234 ± 21.589	65.639 ± 23.634	0.111	1.198	0.198	10.6
2022/23 → 2021/22	V4	Leaf area	1.837 ± 0.478	2.457 ± 1.383	1.297	8.376	1.568	31.9
2022/23 → 2021/22	V4	Total dry matter	1.118 ± 0.247	1.981 ± 1.032	3.49	17.44	3.49	42.5
2022/23 → 2021/22	V4	Branches	0.764 ± 0.710	1.296 ± 0.936	0.749	1.739	0.758	0.6
2022/23 → 2021/22	V4	Main-stem nodes	5.857 ± 0.454	5.921 ± 0.540	0.142	1.419	0.255	13.8
2022/23 → 2021/22	V4	Plant height	13.587 ± 1.604	16.593 ± 2.465	1.874	2.36	1.874	24.4
2022/23 → 2021/22	R2	Leaf area	6.964 ± 4.314	8.390 ± 7.231	0.33	2.81	0.467	9.4
2022/23 → 2021/22	R2	Total dry matter	5.139 ± 3.342	6.523 ± 5.373	0.414	2.585	0.432	9.4
2022/23 → 2021/22	R2	Branches	3.358 ± 2.224	2.810 ± 1.330	−0.246	0.357	0.457	0.0
2022/23 → 2021/22	R2	Main-stem nodes	9.918 ± 1.807	11.509 ± 2.155	0.881	1.422	0.881	13.8
2022/23 → 2021/22	R2	Plant height	32.424 ± 11.154	29.930 ± 10.202	−0.224	0.837	0.234	7.5

Direction	Stage	Trait	Development mean ± SD	Independent-season mean ± SD	SMD	Variance ratio	Scaled W1	Outside range, %
2022/23 → 2021/22	R5	Leaf area	12.891 ± 6.948	14.338 ± 7.448	0.208	1.149	0.235	3.8
2022/23 → 2021/22	R5	Total dry matter	13.207 ± 6.538	19.620 ± 9.615	0.981	2.163	0.981	6.2
2022/23 → 2021/22	R5	Branches	3.231 ± 1.785	3.439 ± 1.246	0.116	0.487	0.264	0.0
2022/23 → 2021/22	R5	Main-stem nodes	13.867 ± 2.473	16.715 ± 2.746	1.152	1.234	1.152	11.2
2022/23 → 2021/22	R5	Plant height	57.831 ± 19.161	60.381 ± 20.083	0.133	1.099	0.217	1.9
2022/23 → 2021/22	R6	Leaf area	7.568 ± 6.033	11.330 ± 5.677	0.623	0.885	0.623	1.9
2022/23 → 2021/22	R6	Total dry matter	22.526 ± 12.144	28.952 ± 12.896	0.529	1.128	0.529	5.6
2022/23 → 2021/22	R6	Branches	2.921 ± 1.597	3.359 ± 1.193	0.275	0.558	0.331	0.0
2022/23 → 2021/22	R6	Main-stem nodes	15.385 ± 3.349	17.697 ± 2.623	0.69	0.614	0.69	3.8
2022/23 → 2021/22	R6	Plant height	65.639 ± 23.634	63.234 ± 21.589	−0.102	0.834	0.181	0.6

Positive SMD values indicate a higher independent-season mean. The variance ratio is the independent-season variance divided by the development-season variance; scaled W1 is the first Wasserstein distance divided by the development-season SD. The outside range is the percentage of independent-season observations beyond the development-season minimum or maximum.

Table S4. Independent-season predictive performance of the information sequence and management comparators.

Transfer direction	Gate/comparator	n	RMSE, kg ha⁻¹ (95% CI)	MAE, kg ha⁻¹ (95% CI)	Predictive R^2 (95% CI)	CCC (95% CI)	Slope (95% CI)	Std. bias (95% CI)
2021/22 → 2022/23	M−1	160	1697.7 (1636.7, 1756.5)	1468.8 (1424.6, 1510.3)	−0.059 (−0.066, −0.052)	−0.000 (−0.000, 0.000)	—	−0.242 (−0.256, −0.228)
2021/22 → 2022/23	M0	160	1398.1 (1325.8, 1448.1)	1057.0 (1009.2, 1110.1)	0.282 (0.252, 0.315)	0.565 (0.547, 0.590)	0.788 (0.759, 0.813)	−0.242 (−0.256, −0.228)
2021/22 → 2022/23	M0A	160	1376.7 (1306.6, 1431.2)	1076.6 (1020.0, 1133.0)	0.304 (0.286, 0.329)	0.568 (0.555, 0.590)	0.827 (0.808, 0.849)	−0.242 (−0.256, −0.228)
2021/22 → 2022/23	M0P	160	1398.1 (1325.8, 1448.1)	1057.0 (1009.2, 1110.1)	0.282 (0.252, 0.315)	0.565 (0.547, 0.590)	0.788 (0.759, 0.813)	−0.242 (−0.256, −0.228)
2021/22 → 2022/23	M1	160	1400.4 (1327.5, 1450.7)	1054.1 (1006.8, 1107.4)	0.279 (0.249, 0.313)	0.566 (0.548, 0.592)	0.786 (0.756, 0.810)	−0.249 (−0.263, −0.236)
2021/22 → 2022/23	M2	160	1397.6 (1322.3, 1449.2)	1053.9 (1003.3, 1107.7)	0.282 (0.251, 0.318)	0.563 (0.545, 0.590)	0.795 (0.764, 0.819)	−0.246 (−0.260, −0.233)
2021/22 → 2022/23	M3	160	1402.9 (1329.1, 1453.6)	1057.5 (1009.3, 1110.7)	0.277 (0.246, 0.311)	0.561 (0.543, 0.587)	0.791 (0.760, 0.816)	−0.249 (−0.262, −0.235)
2021/22 → 2022/23	M4	160	1403.9 (1330.6, 1454.5)	1059.4 (1011.2, 1112.5)	0.276 (0.245, 0.310)	0.561 (0.543, 0.586)	0.787 (0.756, 0.811)	−0.246 (−0.259, −0.232)
2022/23 → 2021/22	M−1	160	1343.4 (1304.6, 1381.1)	1090.0 (1046.9, 1133.1)	−0.098 (−0.132, −0.071)	0.000 (−0.000, 0.000)	—	0.311 (0.266, 0.362)
2022/23 → 2021/22	M0	160	1394.3 (1377.9, 1416.4)	1052.7 (1032.9, 1074.6)	−0.182 (−0.273, −0.109)	0.566 (0.559, 0.576)	0.474 (0.460, 0.488)	0.311 (0.266, 0.362)

Transfer direction	Gate/comparator	n	RMSE, kg ha^{-1} (95% CI)	MAE, kg ha^{-1} (95% CI)	Predictive R^2 (95% CI)	CCC (95% CI)	Slope (95% CI)	Std. bias (95% CI)
2022/23 → 2021/22	M0A	160	1383.9 (1367.7, 1407.4)	1036.8 (1023.7, 1053.5)	−0.165 (−0.252, −0.093)	0.566 (0.558, 0.576)	0.479 (0.465, 0.492)	0.311 (0.266, 0.362)
2022/23 → 2021/22	M0P	160	1386.4 (1369.9, 1408.8)	1044.9 (1024.9, 1067.7)	−0.169 (−0.258, −0.097)	0.567 (0.560, 0.577)	0.478 (0.464, 0.491)	0.311 (0.266, 0.362)
2022/23 → 2021/22	M1	160	1395.8 (1378.3, 1418.6)	1055.7 (1035.1, 1081.1)	−0.185 (−0.277, −0.109)	0.566 (0.558, 0.575)	0.472 (0.458, 0.487)	0.306 (0.262, 0.355)
2022/23 → 2021/22	M2	160	1400.3 (1381.8, 1425.2)	1065.8 (1043.4, 1092.0)	−0.192 (−0.282, −0.120)	0.561 (0.553, 0.570)	0.466 (0.453, 0.480)	0.286 (0.241, 0.337)
2022/23 → 2021/22	M3	160	1401.2 (1382.6, 1423.6)	1049.9 (1032.9, 1073.0)	−0.194 (−0.291, −0.115)	0.566 (0.560, 0.576)	0.473 (0.457, 0.489)	0.323 (0.285, 0.366)
2022/23 → 2021/22	M4	160	1404.2 (1387.1, 1426.5)	1057.3 (1040.1, 1080.1)	−0.199 (−0.293, −0.122)	0.565 (0.559, 0.573)	0.470 (0.455, 0.485)	0.315 (0.276, 0.357)
Pooled reciprocal predictions	M−1	320	1530.8 (1493.0, 1565.4)	1279.4 (1247.2, 1309.1)	−0.054 (−0.060, −0.049)	−0.035 (−0.041, −0.030)	−1.000 (−1.142, −0.842)	0.000 (−0.020, 0.021)
Pooled reciprocal predictions	M0	320	1396.2 (1359.0, 1423.7)	1054.9 (1029.2, 1081.4)	0.123 (0.098, 0.146)	0.558 (0.547, 0.572)	0.563 (0.549, 0.576)	0.000 (−0.020, 0.021)
Pooled reciprocal predictions	M0A	320	1380.3 (1346.1, 1407.8)	1056.7 (1029.0, 1083.3)	0.143 (0.123, 0.162)	0.559 (0.549, 0.571)	0.576 (0.563, 0.589)	−0.000 (−0.020, 0.021)
Pooled reciprocal predictions	M0P	320	1392.3 (1355.0, 1419.9)	1051.0 (1025.0, 1077.8)	0.128 (0.103, 0.151)	0.558 (0.547, 0.572)	0.566 (0.552, 0.579)	0.000 (−0.020, 0.021)
Pooled reciprocal predictions	M1	320	1398.1 (1360.1, 1426.0)	1054.9 (1028.8, 1082.4)	0.121 (0.095, 0.145)	0.558 (0.547, 0.572)	0.561 (0.547, 0.575)	−0.006 (−0.026, 0.014)
Pooled reciprocal predictions	M2	320	1398.9 (1360.3, 1427.9)	1059.9 (1032.4, 1088.0)	0.120 (0.095, 0.143)	0.555 (0.544, 0.569)	0.561 (0.547, 0.575)	−0.013 (−0.034, 0.007)
Pooled reciprocal predictions	M3	320	1402.1 (1363.7, 1430.0)	1053.7 (1027.5, 1080.5)	0.116 (0.088, 0.141)	0.555 (0.544, 0.569)	0.559 (0.544, 0.573)	0.001 (−0.017, 0.020)
Pooled reciprocal predictions	M4	320	1404.1 (1366.0, 1431.9)	1058.3 (1032.2, 1084.9)	0.113 (0.087, 0.138)	0.554 (0.544, 0.569)	0.557 (0.543, 0.571)	−0.001 (−0.019, 0.018)

M−1 is the development-season mean; M0 is the management-combination mean; M0A is the additive management model; M0P is the ridge-regularised management-combination model; and M1–M4 cumulatively add the V4, R2, R5 and R6 phenotypes to M0. Intervals are conditional within-season 95% percentiles from 2000 whole-block resamples. RMSE, root mean squared error; MAE, mean absolute error; CCC, Lin's concordance correlation coefficient. Calibration is undefined for constant M−1 predictions. Pooled rows are descriptive summaries of concatenated reciprocal predictions, not an average of directional transfer success. The intervals condition on the four observed blocks in each evaluation season and fixed development fits.

Table S5. Apparent fit, original tuning, nested within-season validation and independent-season RMSE.

Transfer direction	Gate	Apparent RMSE	Selected tuning RMSE	Nested RMSE	Outer-fold RMSE range	Independent RMSE	Transfer gap
2021/22 → 2022/23	M−1	1282.3	1281.8	1283.0	1220.3–1338.7	1697.7	414.7
2021/22 → 2022/23	M0	183.5	240.0	244.7	166.5–299.6	1398.1	1153.4
2021/22 → 2022/23	M1	181.4	237.4	242.6	159.4–299.9	1400.4	1157.8
2021/22 → 2022/23	M2	176.7	236.9	245.7	159.3–299.6	1397.6	1151.9
2021/22 → 2022/23	M3	179.1	238.9	247.7	164.7–299.6	1402.9	1155.2
2021/22 → 2022/23	M4	179.9	239.1	246.7	164.7–299.6	1403.9	1157.2
2022/23 → 2021/22	M−1	1649.7	1649.2	1650.1	1575.0–1726.3	1343.4	−306.6
2022/23 → 2021/22	M0	210.2	280.3	280.3	276.8–283.4	1394.3	1114.0
2022/23 → 2021/22	M1	207.1	279.4	281.0	279.5–283.8	1395.8	1114.7
2022/23 → 2021/22	M2	206.3	278.7	284.0	276.9–294.0	1400.3	1116.3
2022/23 → 2021/22	M3	200.7	276.4	282.9	270.7–300.9	1401.2	1118.3
2022/23 → 2021/22	M4	200.8	276.9	281.1	274.7–291.7	1404.2	1123.1

Apparent RMSE uses predictions from the full development-season fit on the data used to fit it. Selected tuning RMSE is the mean held-out-block RMSE used to choose the original hyperparameters and can be optimistic after model selection. Nested RMSE pools predictions from four outer held-out blocks, with three-block inner tuning repeated within each outer training set for M1–M4. The outer-fold range is descriptive, not a confidence interval. The transfer gap is independent-season RMSE minus nested development-season RMSE. Nested outer fits use 120 subplots; final transferred fits use 160. All errors are in kg ha^{-1}. For untuned M−1 and M0, the tuning column reports the original mean held-out-block score without selection.

Table S6. Direct and sequential RMSE and MAE gains across the primary information sequence.

Contrast family	Transfer direction	Contrast	Information added	RMSE gain, % (95% CI)	MAE gain, % (95% CI)	Delta predictive R^2 (95% CI)	Resamples ≥2/≥5/≥10%
Management transport	2021/22 → 2022/23	M0 vs M−1	Management combination	17.647 (15.918, 19.318)	28.033 (26.173, 29.939)	0.341 (0.310, 0.367)	2000/2000/2000
Management transport	2022/23 → 2021/22	M0 vs M−1	Management combination	−3.789 (−6.058, −1.722)	3.421 (0.831, 5.814)	−0.085 (−0.141, −0.037)	0/0/0
Management transport	Pooled reciprocal predictions	M0 vs M−1	Management combination	8.794 (7.664, 9.820)	17.549 (16.193, 18.977)	0.177 (0.156, 0.196)	2000/2000/18
Cumulative gate vs M0	2021/22 → 2022/23	M1 vs M0	M1	−0.168 (−0.200, −0.124)	0.282 (0.195, 0.370)	−0.002 (−0.003, −0.002)	0/0/0
Cumulative gate vs M0	2021/22 → 2022/23	M2 vs M0	M2	0.038 (−0.073, 0.247)	0.299 (0.041, 0.639)	0.001 (−0.001, 0.003)	0/0/0
Cumulative gate vs M0	2021/22 → 2022/23	M3 vs M0	M3	−0.345 (−0.405, −0.254)	−0.046 (−0.066, −0.015)	−0.005 (−0.006, −0.003)	0/0/0
Cumulative gate vs M0	2021/22 → 2022/23	M4 vs M0	M4	−0.419 (−0.449, −0.363)	−0.219 (−0.236, −0.198)	−0.006 (−0.007, −0.005)	0/0/0
Cumulative gate vs M0	2022/23 → 2021/22	M1 vs M0	M1	−0.104 (−0.252, 0.075)	−0.280 (−0.665, 0.082)	−0.002 (−0.006, 0.002)	0/0/0
Cumulative gate vs M0	2022/23 → 2021/22	M2 vs M0	M2	−0.427 (−0.630, −0.288)	−1.245 (−1.596, −1.032)	−0.010 (−0.015, −0.007)	0/0/0
Cumulative gate vs M0	2022/23 → 2021/22	M3 vs M0	M3	−0.495 (−0.707, −0.138)	0.264 (−0.219, 1.001)	−0.012 (−0.018, −0.003)	0/0/0
Cumulative gate vs M0	2022/23 → 2021/22	M4 vs M0	M4	−0.712 (−0.857, −0.531)	−0.433 (−0.765, 0.007)	−0.017 (−0.022, −0.012)	0/0/0
Cumulative gate vs M0	Pooled reciprocal predictions	M1 vs M0	M1	−0.136 (−0.214, −0.035)	0.002 (−0.184, 0.187)	−0.002 (−0.004, −0.001)	0/0/0

Contrast family	Transfer direction	Contrast	Information added	RMSE gain, % (95% CI)	MAE gain, % (95% CI)	Delta predictive R^2 (95% CI)	Resamples ≥2/≥5/≥10%
Cumulative gate vs M0	Pooled reciprocal predictions	M2 vs M0	M2	−0.194 (−0.309, −0.077)	−0.472 (−0.691, −0.269)	−0.003 (−0.005, −0.001)	0/0/0
Cumulative gate vs M0	Pooled reciprocal predictions	M3 vs M0	M3	−0.420 (−0.533, −0.242)	0.109 (−0.135, 0.499)	−0.007 (−0.010, −0.004)	0/0/0
Cumulative gate vs M0	Pooled reciprocal predictions	M4 vs M0	M4	−0.565 (−0.643, −0.469)	−0.326 (−0.496, −0.081)	−0.010 (−0.011, −0.008)	0/0/0
Sequential information	2021/22 → 2022/23	M1 vs M0	V4 conditional on management	−0.168 (−0.214, −0.123)	—	—	0/0/0
Sequential information	2021/22 → 2022/23	M2 vs M1	R2 conditional on V4	0.206 (0.046, 0.365)	—	—	0/0/0
Sequential information	2021/22 → 2022/23	M3 vs M2	R5 conditional on V4 and R2	−0.382 (−0.500, −0.265)	—	—	0/0/0
Sequential information	2021/22 → 2022/23	M4 vs M3	R6 conditional on V4, R2 and R5	−0.075 (−0.120, −0.030)	—	—	0/0/0
Sequential information	2022/23 → 2021/22	M1 vs M0	V4 conditional on management	−0.104 (−0.304, 0.097)	—	—	0/0/0
Sequential information	2022/23 → 2021/22	M2 vs M1	R2 conditional on V4	−0.323 (−0.511, −0.135)	—	—	0/0/0
Sequential information	2022/23 → 2021/22	M3 vs M2	R5 conditional on V4 and R2	−0.068 (−0.411, 0.276)	—	—	0/0/0
Sequential information	2022/23 → 2021/22	M4 vs M3	R6 conditional on V4, R2 and R5	−0.215 (−0.406, −0.025)	—	—	0/0/0
Sequential information	Pooled reciprocal predictions	M1 vs M0	V4 conditional on management	−0.136 (−0.241, −0.031)	—	—	0/0/0
Sequential information	Pooled reciprocal predictions	M2 vs M1	R2 conditional on V4	−0.058 (−0.177, 0.060)	—	—	0/0/0
Sequential information	Pooled reciprocal predictions	M3 vs M2	R5 conditional on V4 and R2	−0.225 (−0.408, −0.041)	—	—	0/0/0
Sequential information	Pooled reciprocal predictions	M4 vs M3	R6 conditional on V4, R2 and R5	−0.145 (−0.244, −0.046)	—	—	0/0/0

Positive gains favour the richer model. Direct gate and management contrasts use paired conditional within-season percentile intervals; sequential stage contrasts use simultaneous max-statistic intervals. The final column reports the numbers of the 2000 whole-block resamples attaining RMSE gains of at least 2%, 5% and 10%, respectively. MAE and predictive-R^2 contrasts were not part of the simultaneous stage family and are therefore shown only for direct and management contrasts.

Table S7. Direction-specific correction alignment, correction skill and predicted–observed rank agreement.

Transfer direction	Gate	Correction Pearson r (95% CI)	Zero-correction skill	Prediction Spearman ϱ (95% CI)
2021/22 → 2022/23	M0	—	—	0.572 (0.541, 0.599)
2021/22 → 2022/23	M1	0.093 (0.035, 0.161)	−0.0034	0.572 (0.541, 0.600)
2021/22 → 2022/23	M2	0.090 (0.035, 0.181)	0.0008	0.569 (0.536, 0.598)
2021/22 → 2022/23	M3	−0.098 (−0.156, 0.021)	−0.0069	0.571 (0.540, 0.599)
2021/22 → 2022/23	M4	−0.327 (−0.410, −0.245)	−0.0084	0.571 (0.540, 0.598)
2022/23 → 2021/22	M0	—	—	0.588 (0.574, 0.609)
2022/23 → 2021/22	M1	−0.118 (−0.201, −0.009)	−0.0021	0.584 (0.568, 0.604)
2022/23 → 2021/22	M2	−0.418 (−0.484, −0.355)	−0.0086	0.578 (0.561, 0.599)
2022/23 → 2021/22	M3	−0.013 (−0.131, 0.063)	−0.0099	0.578 (0.560, 0.600)
2022/23 → 2021/22	M4	−0.135 (−0.245, −0.064)	−0.0143	0.577 (0.559, 0.598)

Pearson's r relates $\hat{y}Mk - \hat{y}M0$ to $y - \hat{y}M0$. The zero-correction skill is 1 − SSE(Mk)/SSE(M0); a positive value indicates lower squared error than M0. Spearman's ϱ relates predicted to observed grain yield. Parentheses give conditional within-season 95% percentile intervals from 2000 complete-block resamples. M0 has no phenotypic correction, so its correction correlation and skill are not applicable. Each direction contains 160 subplots nested within four blocks. No between-season or between-site uncertainty is estimated.

Table S8. Sowing-period RMSE and incremental phenotype contribution across M0–M4.

Direction	Period	Gate	n	RMSE, kg ha^{-1}	RMSE gain over M0, %
2021/22 → 2022/23	E1	M0	32	2584.9	0.0
2021/22 → 2022/23	E1	M1	32	2600.9	−0.619
2021/22 → 2022/23	E1	M2	32	2596.1	−0.431
2021/22 → 2022/23	E1	M3	32	2603.8	−0.731
2021/22 → 2022/23	E1	M4	32	2603.9	−0.732
2021/22 → 2022/23	E2	M0	32	1272.9	0.0
2021/22 → 2022/23	E2	M1	32	1267.8	0.404
2021/22 → 2022/23	E2	M2	32	1263.8	0.717
2021/22 → 2022/23	E2	M3	32	1279.6	−0.524
2021/22 → 2022/23	E2	M4	32	1275.7	−0.218
2021/22 → 2022/23	E3	M0	32	889.4	0.0
2021/22 → 2022/23	E3	M1	32	877.7	1.324
2021/22 → 2022/23	E3	M2	32	850.7	4.356
2021/22 → 2022/23	E3	M3	32	864.4	2.82
2021/22 → 2022/23	E3	M4	32	876.4	1.467
2021/22 → 2022/23	E4	M0	32	790.0	0.0
2021/22 → 2022/23	E4	M1	32	779.6	1.318
2021/22 → 2022/23	E4	M2	32	806.1	−2.031
2021/22 → 2022/23	E4	M3	32	787.4	0.337
2021/22 → 2022/23	E4	M4	32	789.6	0.048
2021/22 → 2022/23	E5	M0	32	236.1	0.0
2021/22 → 2022/23	E5	M1	32	236.4	−0.134
2021/22 → 2022/23	E5	M2	32	236.0	0.022
2021/22 → 2022/23	E5	M3	32	237.2	−0.47
2021/22 → 2022/23	E5	M4	32	237.2	−0.492
2022/23 → 2021/22	E1	M0	32	2578.1	0.0
2022/23 → 2021/22	E1	M1	32	2570.3	0.3
2022/23 → 2021/22	E1	M2	32	2564.4	0.529
2022/23 → 2021/22	E1	M3	32	2597.5	−0.753

Direction	Period	Gate	n	RMSE, kg ha⁻¹	RMSE gain over M0, %
2022/23 → 2021/22	E1	M4	32	2597.7	−0.76
2022/23 → 2021/22	E2	M0	32	1259.0	0.0
2022/23 → 2021/22	E2	M1	32	1269.7	−0.846
2022/23 → 2021/22	E2	M2	32	1250.7	0.662
2022/23 → 2021/22	E2	M3	32	1289.7	−2.437
2022/23 → 2021/22	E2	M4	32	1280.3	−1.69
2022/23 → 2021/22	E3	M0	32	899.1	0.0
2022/23 → 2021/22	E3	M1	32	907.8	−0.962
2022/23 → 2021/22	E3	M2	32	963.9	−7.209
2022/23 → 2021/22	E3	M3	32	824.6	8.284
2022/23 → 2021/22	E3	M4	32	846.6	5.841
2022/23 → 2021/22	E4	M0	32	790.5	0.0
2022/23 → 2021/22	E4	M1	32	800.9	−1.316
2022/23 → 2021/22	E4	M2	32	822.7	−4.066
2022/23 → 2021/22	E4	M3	32	819.5	−3.662
2022/23 → 2021/22	E4	M4	32	836.1	−5.764
2022/23 → 2021/22	E5	M0	32	236.0	0.0
2022/23 → 2021/22	E5	M1	32	237.7	−0.713
2022/23 → 2021/22	E5	M2	32	239.5	−1.482
2022/23 → 2021/22	E5	M3	32	235.0	0.43
2022/23 → 2021/22	E5	M4	32	237.9	−0.805

Each sowing period contains n = 32 independent-season subplot observations within four main plots. A positive gain indicates a lower RMSE than the matched M0 prediction does. The estimates are descriptive because model tuning uses the complete development season.

Table S9. Robustness across algorithms, plant-scale representations, management comparators, accumulation rules, sowing domains, target scales and bootstrap schemes.

Bootstrap scheme	Analysis	Direction	Specification	Gate-wise gain range, %	Gate with largest gain	Largest gain, % (95% CI)	Maximum resamples ≥2/≥5/≥10%
Whole-block bootstrap	Phenotype model vs matched management comparator	2021/22 → 2022/23	Primary compact cumulative elastic net	−0.419 to 0.038	M2	0.038 (−0.073, 0.247)	0/0/0
Sowing-period-stratified main-plot bootstrap	Phenotype model vs matched management comparator	2021/22 → 2022/23	Primary compact cumulative elastic net	−0.419 to 0.038	M2	0.038 (−0.141, 0.248)	0/0/0
Whole-block bootstrap	Phenotype model vs matched management comparator	2022/23 → 2021/22	Primary compact cumulative elastic net	−0.712 to −0.104	M1	−0.104 (−0.252, 0.075)	0/0/0
Sowing-period-stratified main-plot bootstrap	Phenotype model vs matched management comparator	2022/23 → 2021/22	Primary compact cumulative elastic net	−0.712 to −0.104	M1	−0.104 (−0.428, 0.229)	0/0/0
Whole-block bootstrap	Phenotype model vs matched management comparator	Pooled reciprocal predictions	Primary compact cumulative elastic net	−0.565 to −0.136	M1	−0.136 (−0.214, −0.035)	0/0/0

Bootstrap scheme	Analysis	Direction	Specification	Gate-wise gain range, %	Gate with largest gain	Largest gain, % (95% CI)	Maximum resamples ≥2/≥5/≥10%
Sowing-period-stratified main-plot bootstrap	Phenotype model vs matched management comparator	Pooled reciprocal predictions	Primary compact cumulative elastic net	−0.565 to −0.136	M1	−0.136 (−0.302, 0.030)	0/0/0
Whole-block bootstrap	Phenotype model vs matched management comparator	2021/22 → 2022/23	PLS, compact cumulative basis	−0.484 to −0.213	M2	−0.213 (−0.252, −0.128)	0/0/0
Sowing-period-stratified main-plot bootstrap	Phenotype model vs matched management comparator	2021/22 → 2022/23	PLS, compact cumulative basis	−0.484 to −0.213	M2	−0.213 (−0.295, −0.096)	0/0/0
Whole-block bootstrap	Phenotype model vs matched management comparator	2022/23 → 2021/22	PLS, compact cumulative basis	−1.094 to −0.071	M4	−0.071 (−0.329, 0.192)	0/0/0
Sowing-period-stratified main-plot bootstrap	Phenotype model vs matched management comparator	2022/23 → 2021/22	PLS, compact cumulative basis	−1.094 to −0.071	M4	−0.071 (−0.789, 0.705)	0/0/0
Whole-block bootstrap	Phenotype model vs matched management comparator	Pooled reciprocal predictions	PLS, compact cumulative basis	−0.720 to −0.239	M1	−0.239 (−0.354, −0.081)	0/0/0
Sowing-period-stratified main-plot bootstrap	Phenotype model vs matched management comparator	Pooled reciprocal predictions	PLS, compact cumulative basis	−0.720 to −0.239	M1	−0.239 (−0.486, 0.014)	0/0/0
Whole-block bootstrap	Phenotype model vs matched management comparator	2021/22 → 2022/23	Stage-only compact elastic net	−0.168 to 0.572	M4	0.572 (0.293, 0.770)	0/0/0
Sowing-period-stratified main-plot bootstrap	Phenotype model vs matched management comparator	2021/22 → 2022/23	Stage-only compact elastic net	−0.168 to 0.572	M4	0.572 (0.372, 0.789)	0/0/0
Whole-block bootstrap	Phenotype model vs matched management comparator	2022/23 → 2021/22	Stage-only compact elastic net	−0.563 to 0.060	M2	0.060 (0.042, 0.072)	0/0/0
Sowing-period-stratified main-plot bootstrap	Phenotype model vs matched management comparator	2022/23 → 2021/22	Stage-only compact elastic net	−0.563 to 0.060	M2	0.060 (0.042, 0.078)	0/0/0
Whole-block bootstrap	Phenotype model vs matched management comparator	Pooled reciprocal predictions	Stage-only compact elastic net	−0.281 to 0.274	M4	0.274 (0.140, 0.373)	0/0/0

Bootstrap scheme	Analysis	Direction	Specification	Gate-wise gain range, %	Gate with largest gain	Largest gain, % (95% CI)	Maximum resamples ≥2/≥5/≥10%
Sowing-period-stratified main-plot bootstrap	Phenotype model vs matched management comparator	Pooled reciprocal predictions	Stage-only compact elastic net	−0.281 to 0.274	M4	0.274 (0.183, 0.371)	0/0/0
Whole-block bootstrap	Phenotype model vs matched management comparator	2021/22 → 2022/23	Expanded plant basis	−0.529 to −0.129	M1	−0.129 (−0.163, −0.096)	0/0/0
Sowing-period-stratified main-plot bootstrap	Phenotype model vs matched management comparator	2021/22 → 2022/23	Expanded plant basis	−0.529 to −0.129	M1	−0.129 (−0.186, −0.062)	0/0/0
Whole-block bootstrap	Phenotype model vs matched management comparator	2022/23 → 2021/22	Expanded plant basis	−0.507 to −0.347	M1	−0.347 (−0.468, −0.209)	0/0/0
Sowing-period-stratified main-plot bootstrap	Phenotype model vs matched management comparator	2022/23 → 2021/22	Expanded plant basis	−0.507 to −0.347	M1	−0.347 (−0.610, −0.081)	0/0/0
Whole-block bootstrap	Phenotype model vs matched management comparator	Pooled reciprocal predictions	Expanded plant basis	−0.484 to −0.238	M1	−0.238 (−0.301, −0.166)	0/0/0
Sowing-period-stratified main-plot bootstrap	Phenotype model vs matched management comparator	Pooled reciprocal predictions	Expanded plant basis	−0.484 to −0.238	M1	−0.238 (−0.374, −0.103)	0/0/0
Whole-block bootstrap	Phenotype model vs matched management comparator	2021/22 → 2022/23	Organ-component basis	−0.489 to −0.128	M1	−0.128 (−0.158, −0.090)	0/0/0
Sowing-period-stratified main-plot bootstrap	Phenotype model vs matched management comparator	2021/22 → 2022/23	Organ-component basis	−0.489 to −0.128	M1	−0.128 (−0.178, −0.062)	0/0/0
Whole-block bootstrap	Phenotype model vs matched management comparator	2022/23 → 2021/22	Organ-component basis	−0.683 to −0.427	M2	−0.427 (−0.630, −0.288)	0/0/0
Sowing-period-stratified main-plot bootstrap	Phenotype model vs matched management comparator	2022/23 → 2021/22	Organ-component basis	−0.683 to −0.427	M2	−0.427 (−0.857, 0.009)	0/0/0
Whole-block bootstrap	Phenotype model vs matched management comparator	Pooled reciprocal predictions	Organ-component basis	−0.464 to −0.310	M1	−0.310 (−0.389, −0.225)	0/0/0

Bootstrap scheme	Analysis	Direction	Specification	Gate-wise gain range, %	Gate with largest gain	Largest gain, % (95% CI)	Maximum resamples ≥2/≥5/≥10%
Sowing-period-stratified main-plot bootstrap	Phenotype model vs matched management comparator	Pooled reciprocal predictions	Organ-component basis	−0.464 to −0.310	M1	−0.310 (−0.485, −0.132)	0/0/0
Whole-block bootstrap	Phenotype model vs matched management comparator	2021/22 → 2022/23	Total-node basis	−0.462 to −0.053	M4	−0.053 (−0.081, −0.031)	0/0/0
Sowing-period-stratified main-plot bootstrap	Phenotype model vs matched management comparator	2021/22 → 2022/23	Total-node basis	−0.462 to −0.053	M4	−0.053 (−0.080, −0.024)	0/0/0
Whole-block bootstrap	Phenotype model vs matched management comparator	2022/23 → 2021/22	Total-node basis	−0.288 to −0.202	M4	−0.202 (−0.263, −0.133)	0/0/0
Sowing-period-stratified main-plot bootstrap	Phenotype model vs matched management comparator	2022/23 → 2021/22	Total-node basis	−0.288 to −0.202	M4	−0.202 (−0.299, −0.101)	0/0/0
Whole-block bootstrap	Phenotype model vs matched management comparator	Pooled reciprocal predictions	Total-node basis	−0.336 to −0.128	M4	−0.128 (−0.162, −0.090)	0/0/0
Sowing-period-stratified main-plot bootstrap	Phenotype model vs matched management comparator	Pooled reciprocal predictions	Total-node basis	−0.336 to −0.128	M4	−0.128 (−0.179, −0.074)	0/0/0
Whole-block bootstrap	Phenotype model vs matched management comparator	2021/22 → 2022/23	Stature-assessment node basis	−0.420 to 0.037	M2	0.037 (−0.075, 0.247)	0/0/0
Sowing-period-stratified main-plot bootstrap	Phenotype model vs matched management comparator	2021/22 → 2022/23	Stature-assessment node basis	−0.420 to 0.037	M2	0.037 (−0.142, 0.248)	0/0/0
Whole-block bootstrap	Phenotype model vs matched management comparator	2022/23 → 2021/22	Stature-assessment node basis	−0.709 to −0.104	M1	−0.104 (−0.252, 0.075)	0/0/0
Sowing-period-stratified main-plot bootstrap	Phenotype model vs matched management comparator	2022/23 → 2021/22	Stature-assessment node basis	−0.709 to −0.104	M1	−0.104 (−0.428, 0.229)	0/0/0
Whole-block bootstrap	Phenotype model vs matched management comparator	Pooled reciprocal predictions	Stature-assessment node basis	−0.564 to −0.136	M1	−0.136 (−0.214, −0.035)	0/0/0

Bootstrap scheme	Analysis	Direction	Specification	Gate-wise gain range, %	Gate with largest gain	Largest gain, % (95% CI)	Maximum resamples ≥2/≥5/≥10%
Sowing-period-stratified main-plot bootstrap	Phenotype model vs matched management comparator	Pooled reciprocal predictions	Stature-assessment node basis	−0.564 to −0.136	M1	−0.136 (−0.302, 0.030)	0/0/0
Whole-block bootstrap	Phenotype model vs matched management comparator	2021/22 → 2022/23	Internode basis	−0.104 to 0.440	M2	0.440 (0.285, 0.612)	0/0/0
Sowing-period-stratified main-plot bootstrap	Phenotype model vs matched management comparator	2021/22 → 2022/23	Internode basis	−0.104 to 0.440	M2	0.440 (0.282, 0.607)	0/0/0
Whole-block bootstrap	Phenotype model vs matched management comparator	2022/23 → 2021/22	Internode basis	−0.267 to −0.113	M2	−0.113 (−0.233, 0.007)	0/0/0
Sowing-period-stratified main-plot bootstrap	Phenotype model vs matched management comparator	2022/23 → 2021/22	Internode basis	−0.267 to −0.113	M2	−0.113 (−0.384, 0.149)	0/0/0
Whole-block bootstrap	Phenotype model vs matched management comparator	Pooled reciprocal predictions	Internode basis	−0.185 to 0.164	M2	0.164 (0.072, 0.261)	0/0/0
Sowing-period-stratified main-plot bootstrap	Phenotype model vs matched management comparator	Pooled reciprocal predictions	Internode basis	−0.185 to 0.164	M2	0.164 (0.007, 0.315)	0/0/0
Whole-block bootstrap	Phenotype model vs matched management comparator	2021/22 → 2022/23	E1–E4 compact cumulative elastic net	−0.729 to −0.012	M2	−0.012 (−0.108, 0.147)	0/0/0
Sowing-period-stratified main-plot bootstrap	Phenotype model vs matched management comparator	2021/22 → 2022/23	E1–E4 compact cumulative elastic net	−0.729 to −0.012	M2	−0.012 (−0.186, 0.175)	0/0/0
Whole-block bootstrap	Phenotype model vs matched management comparator	2022/23 → 2021/22	E1–E4 compact cumulative elastic net	−0.158 to 0.514	M2	0.514 (0.290, 0.710)	0/0/0
Sowing-period-stratified main-plot bootstrap	Phenotype model vs matched management comparator	2022/23 → 2021/22	E1–E4 compact cumulative elastic net	−0.158 to 0.514	M2	0.514 (0.084, 0.936)	0/0/0
Whole-block bootstrap	Phenotype model vs matched management comparator	Pooled reciprocal predictions	E1–E4 compact cumulative elastic net	−0.412 to 0.250	M2	0.250 (0.129, 0.373)	0/0/0

Bootstrap scheme	Analysis	Direction	Specification	Gate-wise gain range, %	Gate with largest gain	Largest gain, % (95% CI)	Maximum resamples ≥2/≥5/≥10%
Sowing-period-stratified main-plot bootstrap	Phenotype model vs matched management comparator	Pooled reciprocal predictions	E1–E4 compact cumulative elastic net	−0.412 to 0.250	M2	0.250 (0.018, 0.487)	0/0/0
Whole-block bootstrap	Phenotype model vs matched management comparator	2021/22 → 2022/23	Log-yield compact cumulative elastic net	−0.012 to 1.497	M2	1.497 (1.359, 1.643)	0/0/0
Sowing-period-stratified main-plot bootstrap	Phenotype model vs matched management comparator	2021/22 → 2022/23	Log-yield compact cumulative elastic net	−0.012 to 1.497	M2	1.497 (1.213, 1.813)	1/0/0
Whole-block bootstrap	Phenotype model vs matched management comparator	2022/23 → 2021/22	Log-yield compact cumulative elastic net	−0.329 to −0.145	M4	−0.145 (−0.647, 0.389)	0/0/0
Sowing-period-stratified main-plot bootstrap	Phenotype model vs matched management comparator	2022/23 → 2021/22	Log-yield compact cumulative elastic net	−0.329 to −0.145	M4	−0.145 (−0.998, 0.720)	0/0/0
Whole-block bootstrap	Phenotype model vs matched management comparator	Pooled reciprocal predictions	Log-yield compact cumulative elastic net	−0.159 to 0.600	M2	0.600 (0.432, 0.779)	0/0/0
Sowing-period-stratified main-plot bootstrap	Phenotype model vs matched management comparator	Pooled reciprocal predictions	Log-yield compact cumulative elastic net	−0.159 to 0.600	M2	0.600 (0.252, 0.926)	0/0/0
Whole-block bootstrap	Phenotype model vs matched management comparator	2021/22 → 2022/23	Compact basis with additive management comparator	−0.622 to −0.075	M1	−0.075 (−0.105, −0.041)	0/0/0
Sowing-period-stratified main-plot bootstrap	Phenotype model vs matched management comparator	2021/22 → 2022/23	Compact basis with additive management comparator	−0.622 to −0.075	M1	−0.075 (−0.115, −0.024)	0/0/0
Whole-block bootstrap	Phenotype model vs matched management comparator	2022/23 → 2021/22	Compact basis with additive management comparator	−0.622 to 0.000	M1	0.000 (0.000, 0.000)	0/0/0
Sowing-period-stratified main-plot bootstrap	Phenotype model vs matched management comparator	2022/23 → 2021/22	Compact basis with additive management comparator	−0.622 to 0.000	M1	0.000 (0.000, 0.000)	0/0/0
Whole-block bootstrap	Phenotype model vs matched management comparator	Pooled reciprocal predictions	Compact basis with additive management comparator	−0.395 to −0.037	M1	−0.037 (−0.054, −0.019)	0/0/0

Bootstrap scheme	Analysis	Direction	Specification	Gate-wise gain range, %	Gate with largest gain	Largest gain, % (95% CI)	Maximum resamples ≥2/≥5/≥10%
Sowing-period-stratified main-plot bootstrap	Phenotype model vs matched management comparator	Pooled reciprocal predictions	Compact basis with additive management comparator	−0.395 to −0.037	M1	−0.037 (−0.058, −0.012)	0/0/0
Whole-block bootstrap	Phenotype model vs matched management comparator	2021/22 → 2022/23	Compact basis with ridge-regularised management comparator	−0.419 to 0.038	M2	0.038 (−0.073, 0.247)	0/0/0
Sowing-period-stratified main-plot bootstrap	Phenotype model vs matched management comparator	2021/22 → 2022/23	Compact basis with ridge-regularised management comparator	−0.419 to 0.038	M2	0.038 (−0.141, 0.248)	0/0/0
Whole-block bootstrap	Phenotype model vs matched management comparator	2022/23 → 2021/22	Compact basis with ridge-regularised management comparator	−0.456 to 0.365	M1	0.365 (0.144, 0.639)	0/0/0
Sowing-period-stratified main-plot bootstrap	Phenotype model vs matched management comparator	2022/23 → 2021/22	Compact basis with ridge-regularised management comparator	−0.456 to 0.365	M1	0.365 (0.070, 0.687)	0/0/0
Whole-block bootstrap	Phenotype model vs matched management comparator	Pooled reciprocal predictions	Compact basis with ridge-regularised management comparator	−0.437 to 0.096	M1	0.096 (−0.012, 0.231)	0/0/0
Sowing-period-stratified main-plot bootstrap	Phenotype model vs matched management comparator	Pooled reciprocal predictions	Compact basis with ridge-regularised management comparator	−0.437 to 0.096	M1	0.096 (−0.064, 0.261)	0/0/0
Whole-block bootstrap	Direct representation vs compact plant basis	2021/22 → 2022/23	expanded plant basis	−0.567 to 0.046	M4	0.046 (−0.025, 0.094)	0/0/0
Sowing-period-stratified main-plot bootstrap	Direct representation vs compact plant basis	2021/22 → 2022/23	expanded plant basis	−0.567 to 0.046	M4	0.046 (−0.029, 0.110)	0/0/0
Whole-block bootstrap	Direct representation vs compact plant basis	2022/23 → 2021/22	expanded plant basis	−0.243 to 0.203	M4	0.203 (0.078, 0.287)	0/0/0
Sowing-period-stratified main-plot bootstrap	Direct representation vs compact plant basis	2022/23 → 2021/22	expanded plant basis	−0.243 to 0.203	M4	0.203 (−0.096, 0.491)	0/0/0

Bootstrap scheme	Analysis	Direction	Specification	Gate-wise gain range, %	Gate with largest gain	Largest gain, % (95% CI)	Maximum resamples ≥2/≥5/≥10%
Whole-block bootstrap	Direct representation vs compact plant basis	Pooled reciprocal predictions	expanded plant basis	−0.289 to 0.124	M4	0.124 (0.052, 0.182)	0/0/0
Sowing-period-stratified main-plot bootstrap	Direct representation vs compact plant basis	Pooled reciprocal predictions	expanded plant basis	−0.289 to 0.124	M4	0.124 (−0.028, 0.279)	0/0/0
Whole-block bootstrap	Direct representation vs compact plant basis	2021/22 → 2022/23	organ-component basis	−0.527 to 0.123	M4	0.123 (0.091, 0.155)	0/0/0
Sowing-period-stratified main-plot bootstrap	Direct representation vs compact plant basis	2021/22 → 2022/23	organ-component basis	−0.527 to 0.123	M4	0.123 (0.078, 0.166)	0/0/0
Whole-block bootstrap	Direct representation vs compact plant basis	2022/23 → 2021/22	organ-component basis	−0.389 to 0.118	M4	0.118 (−0.019, 0.226)	0/0/0
Sowing-period-stratified main-plot bootstrap	Direct representation vs compact plant basis	2022/23 → 2021/22	organ-component basis	−0.389 to 0.118	M4	0.118 (−0.176, 0.400)	0/0/0
Whole-block bootstrap	Direct representation vs compact plant basis	Pooled reciprocal predictions	organ-component basis	−0.263 to 0.121	M4	0.121 (0.053, 0.181)	0/0/0
Sowing-period-stratified main-plot bootstrap	Direct representation vs compact plant basis	Pooled reciprocal predictions	organ-component basis	−0.263 to 0.121	M4	0.121 (−0.027, 0.274)	0/0/0
Whole-block bootstrap	Direct representation vs compact plant basis	2021/22 → 2022/23	total-node basis	−0.500 to 0.365	M4	0.365 (0.310, 0.409)	0/0/0
Sowing-period-stratified main-plot bootstrap	Direct representation vs compact plant basis	2021/22 → 2022/23	total-node basis	−0.500 to 0.365	M4	0.365 (0.282, 0.428)	0/0/0
Whole-block bootstrap	Direct representation vs compact plant basis	2022/23 → 2021/22	total-node basis	−0.185 to 0.506	M4	0.506 (0.307, 0.658)	0/0/0
Sowing-period-stratified main-plot bootstrap	Direct representation vs compact plant basis	2022/23 → 2021/22	total-node basis	−0.185 to 0.506	M4	0.506 (−0.012, 1.012)	0/0/0

Bootstrap scheme	Analysis	Direction	Specification	Gate-wise gain range, %	Gate with largest gain	Largest gain, % (95% CI)	Maximum resamples ≥2/≥5/≥10%
Whole-block bootstrap	Direct representation vs compact plant basis	Pooled reciprocal predictions	total-node basis	−0.184 to 0.435	M4	0.435 (0.336, 0.516)	0/0/0
Sowing-period-stratified main-plot bootstrap	Direct representation vs compact plant basis	Pooled reciprocal predictions	total-node basis	−0.184 to 0.435	M4	0.435 (0.176, 0.704)	0/0/0
Whole-block bootstrap	Direct representation vs compact plant basis	2021/22 → 2022/23	stature-assessment node basis	−0.001 to 0.000	M1	0.000 (0.000, 0.000)	0/0/0
Sowing-period-stratified main-plot bootstrap	Direct representation vs compact plant basis	2021/22 → 2022/23	stature-assessment node basis	−0.001 to 0.000	M1	0.000 (0.000, 0.000)	0/0/0
Whole-block bootstrap	Direct representation vs compact plant basis	2022/23 → 2021/22	stature-assessment node basis	−0.002 to 0.003	M3	0.003 (0.002, 0.005)	0/0/0
Sowing-period-stratified main-plot bootstrap	Direct representation vs compact plant basis	2022/23 → 2021/22	stature-assessment node basis	−0.002 to 0.003	M3	0.003 (0.001, 0.005)	0/0/0
Whole-block bootstrap	Direct representation vs compact plant basis	Pooled reciprocal predictions	stature-assessment node basis	−0.001 to 0.002	M3	0.002 (0.001, 0.002)	0/0/0
Sowing-period-stratified main-plot bootstrap	Direct representation vs compact plant basis	Pooled reciprocal predictions	stature-assessment node basis	−0.001 to 0.002	M3	0.002 (0.000, 0.003)	0/0/0
Whole-block bootstrap	Direct representation vs compact plant basis	2021/22 → 2022/23	internode basis	0.065 to 0.403	M2	0.403 (0.307, 0.493)	0/0/0
Sowing-period-stratified main-plot bootstrap	Direct representation vs compact plant basis	2021/22 → 2022/23	internode basis	0.065 to 0.403	M2	0.403 (0.325, 0.481)	0/0/0
Whole-block bootstrap	Direct representation vs compact plant basis	2022/23 → 2021/22	internode basis	−0.164 to 0.525	M4	0.525 (0.430, 0.698)	0/0/0
Sowing-period-stratified main-plot bootstrap	Direct representation vs compact plant basis	2022/23 → 2021/22	internode basis	−0.164 to 0.525	M4	0.525 (0.139, 0.887)	0/0/0

Bootstrap scheme	Analysis	Direction	Specification	Gate-wise gain range, %	Gate with largest gain	Largest gain, % (95% CI)	Maximum resamples ≥2/≥5/≥10%
Whole-block bootstrap	Direct representation vs compact plant basis	Pooled reciprocal predictions	internode basis	−0.049 to 0.433	M4	0.433 (0.376, 0.519)	0/0/0
Sowing-period-stratified main-plot bootstrap	Direct representation vs compact plant basis	Pooled reciprocal predictions	internode basis	−0.049 to 0.433	M4	0.433 (0.241, 0.626)	0/0/0

Rows summarise the M1–M4 RMSE-gain range within each specification and bootstrap scheme, identify the gate with the largest observed gain and report the maximum threshold-attainment counts across the four gates. Direct representation contrasts are summarised analogously relative to the row-matched compact plant basis. The selected gate is descriptive and does not denote model selection. For the primary M0 comparison, exact enumeration of all 256 ordered whole-block resamples (35 unique multiplicity configurations) produced zero configurations at or above 2%, 5% or 10% in either direction and every gate.

Table S10. Hyperparameters selected by grouped development-season cross-validation.

Direction	Model/gate	Numeric predictors	Selected penalty or components	L1 proportion	Inner-CV RMSE, kg ha^{-1}
2021/22 → 2022/23	M0P	40 cell indicators	ridge λ = 0.0001	—	240.0
2021/22 → 2022/23	Elastic net M1	5	α = 0.5135	0.05	237.4
2021/22 → 2022/23	Elastic net M2	10	α = 1.528	0.95	236.9
2021/22 → 2022/23	Elastic net M3	15	α = 0.8859	0.05	238.9
2021/22 → 2022/23	Elastic net M4	20	α = 1.528	0.05	239.1
2021/22 → 2022/23	PLS M1	5	1 component	—	236.2
2021/22 → 2022/23	PLS M2	10	1 component	—	237.3
2021/22 → 2022/23	PLS M3	15	1 component	—	239.3
2021/22 → 2022/23	PLS M4	20	1 component	—	239.8
2022/23 → 2021/22	M0P	40 cell indicators	ridge λ = 0.0316228	—	280.0
2022/23 → 2021/22	Elastic net M1	5	α = 0.5135	0.05	279.4
2022/23 → 2021/22	Elastic net M2	10	α = 4.549	0.95	278.7
2022/23 → 2021/22	Elastic net M3	15	α = 0.1	0.05	276.4
2022/23 → 2021/22	Elastic net M4	20	α = 0.1725	0.05	276.9
2022/23 → 2021/22	PLS M1	5	2 components	—	279.1
2022/23 → 2021/22	PLS M2	10	2 components	—	279.2
2022/23 → 2021/22	PLS M3	15	5 components	—	275.0
2022/23 → 2021/22	PLS M4	20	3 components	—	278.0

The table reports selected hyperparameters for the primary compact cumulative elastic-net models, the corresponding PLS sensitivity models and the ridge-regularised management comparator M0P. Four leave-one-block-out folds were used within the development season. Predictor counts refer to cumulative phenotypic variables, except for the 40 M0P cell indicators. RMSE, root mean squared error. Elastic-net candidates comprised the 20 α values defined in the Supplementary Methods crossed with L1 proportions 0.05, 0.25, 0.50, 0.75 and 0.95. The reported inner RMSE was used for selection and is distinct from the nested outer-fold assessment in Table S5.

Supplementary Figures

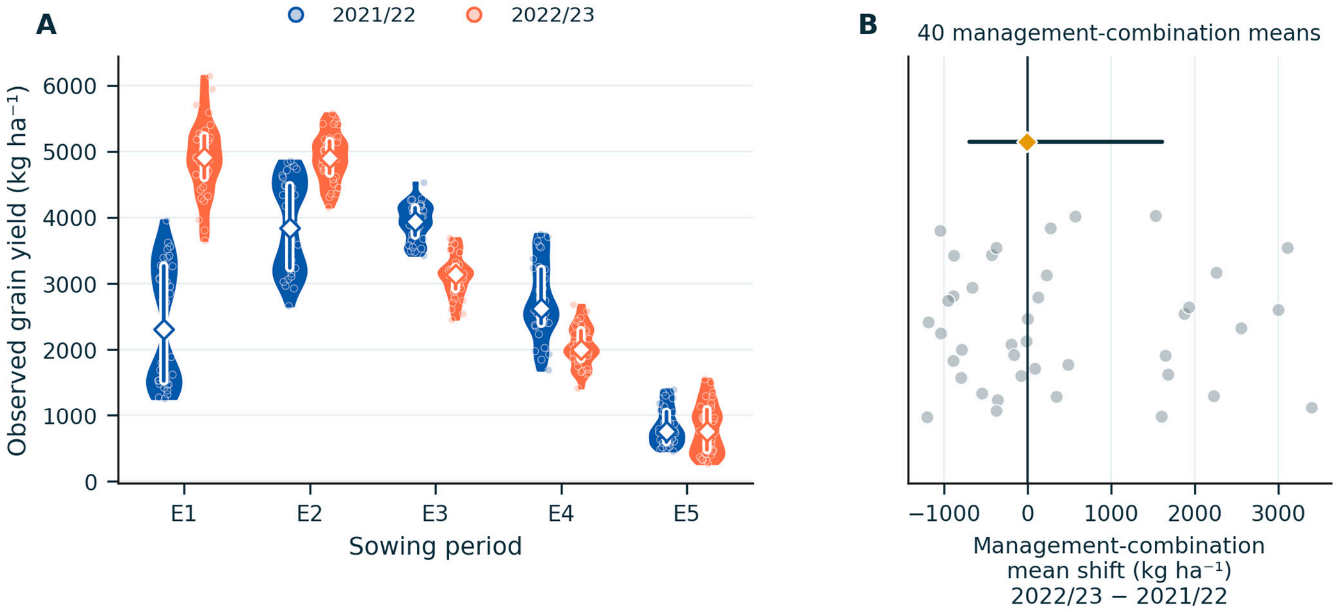

Figure S1. Observed grain yield by sowing period and management-combination shifts between seasons. Panel **A** shows season-specific violin distributions, jittered subplot observations, interquartile ranges and median diamonds within sowing periods (n = 32 per season × period). Panel **B** shows the 2022/23–2021/22 difference in mean yield for each of the 40 sowing-period × cultivar × seeding-rate combinations. Each seasonal combination mean averages four blocks; points denote combination-specific differences, the segment spans their interquartile range and the diamond marks their median.

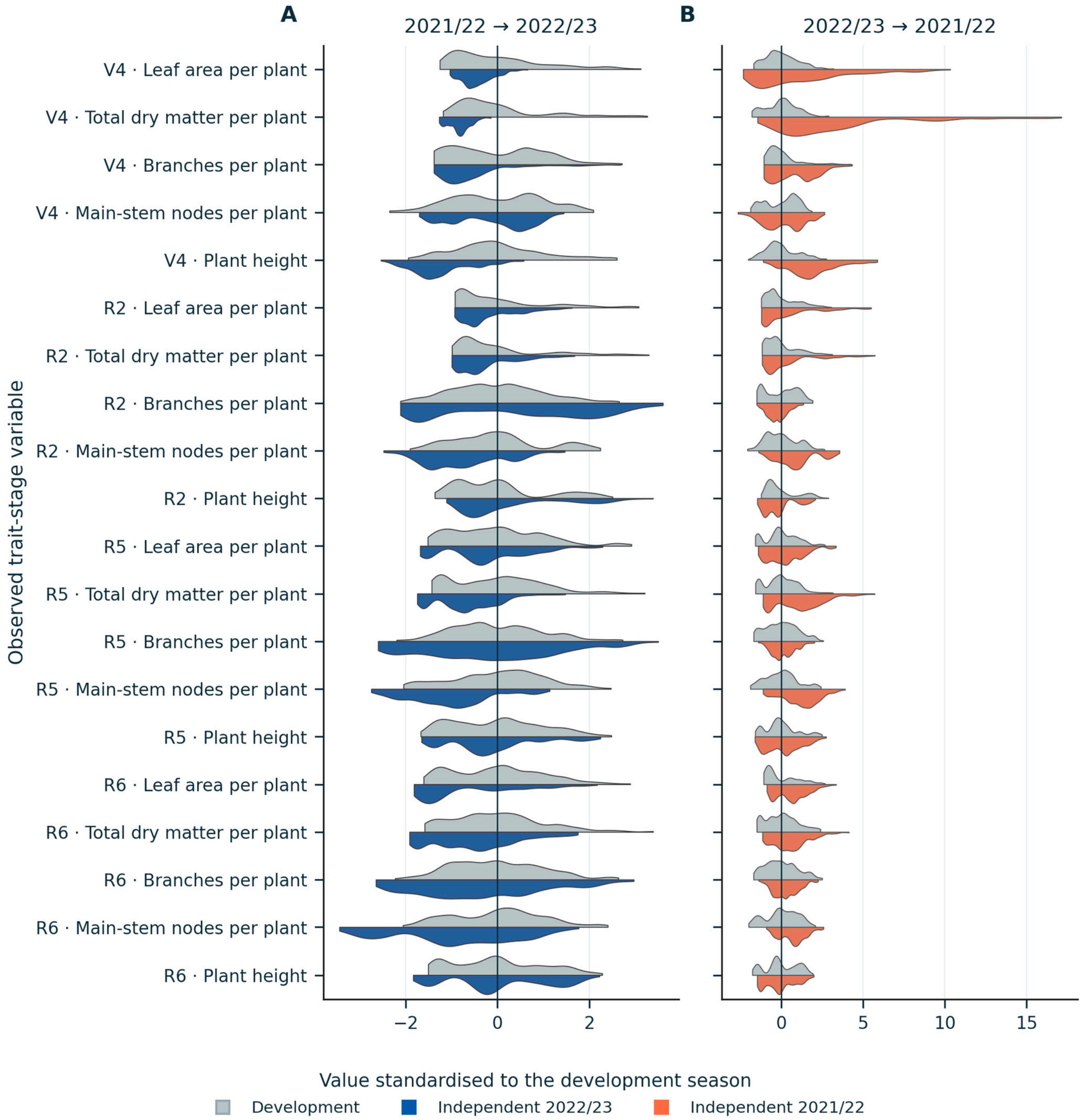

Figure S2. Development-standardised predictor domains in reciprocal transfer. Panels **A** and **B** show split-violin distributions for the 20 compact trait–stage variables in the two reciprocal transfer directions. Within each panel, development and independent-season observations (n = 160 per distribution) are expressed relative to the development-season mean and standard deviation; zero denotes the development-season mean. The values are dimensionless and compare the distributional support across temporal transfers.

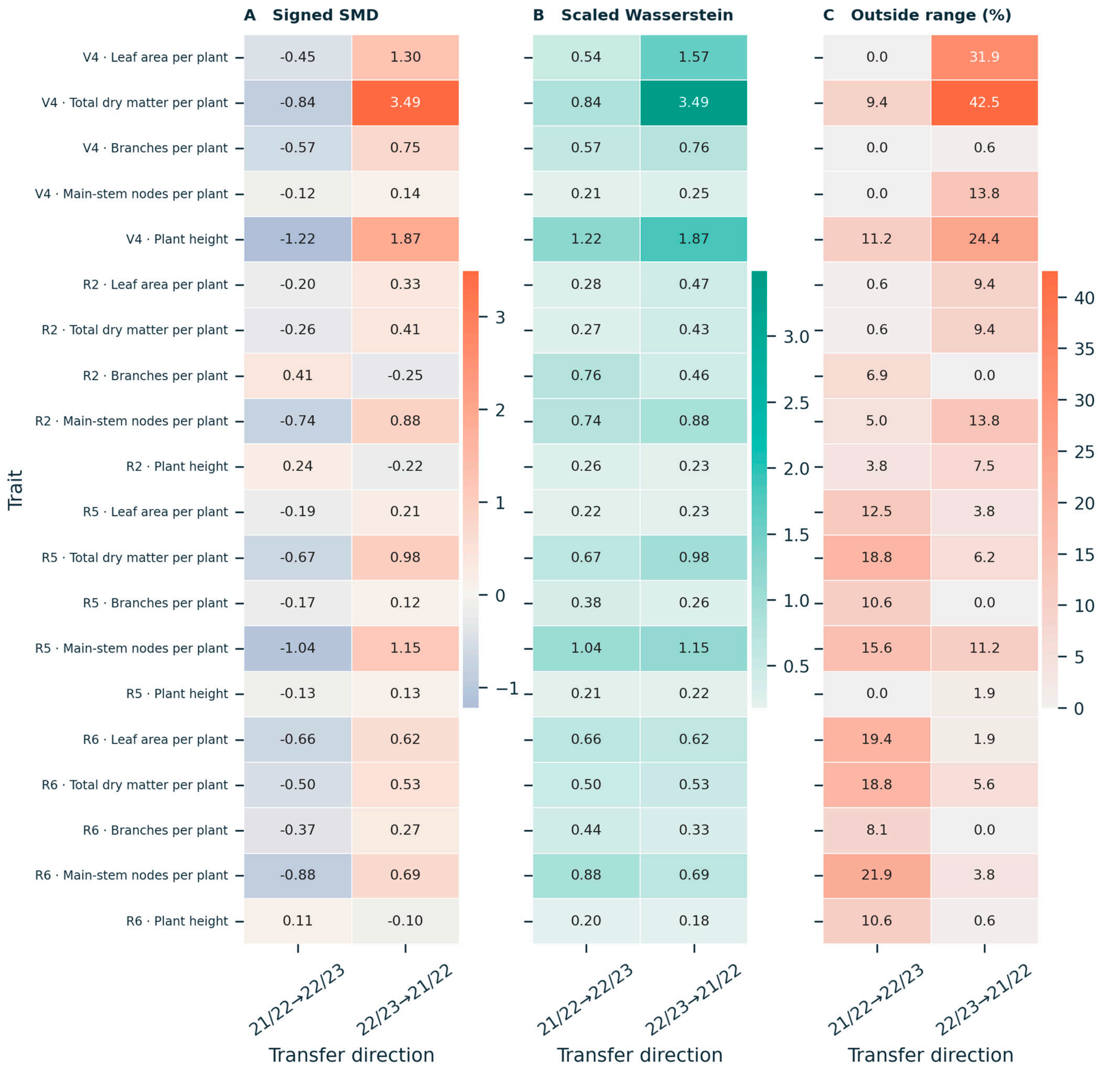

Trait	A 21/22→22/23	A 22/23→21/22	B 21/22→22/23	B 22/23→21/22	C 21/22→22/23	C 22/23→21/22
V4 · Leaf area per plant	-0.45	1.30	0.54	1.57	0.0	31.9
V4 · Total dry matter per plant	-0.84	3.49	0.84	3.49	9.4	42.5
V4 · Branches per plant	-0.57	0.75	0.57	0.76	0.0	0.6
V4 · Main-stem nodes per plant	-0.12	0.14	0.21	0.25	0.0	13.8
V4 · Plant height	-1.22	1.87	1.22	1.87	11.2	24.4
R2 · Leaf area per plant	-0.20	0.33	0.28	0.47	0.6	9.4
R2 · Total dry matter per plant	-0.26	0.41	0.27	0.43	0.6	9.4
R2 · Branches per plant	0.41	-0.25	0.76	0.46	6.9	0.0
R2 · Main-stem nodes per plant	-0.74	0.88	0.74	0.88	5.0	13.8
R2 · Plant height	0.24	-0.22	0.26	0.23	3.8	7.5
R5 · Leaf area per plant	-0.19	0.21	0.22	0.23	12.5	3.8
R5 · Total dry matter per plant	-0.67	0.98	0.67	0.98	18.8	6.2
R5 · Branches per plant	-0.17	0.12	0.38	0.26	10.6	0.0
R5 · Main-stem nodes per plant	-1.04	1.15	1.04	1.15	15.6	11.2
R5 · Plant height	-0.13	0.13	0.21	0.22	0.0	1.9
R6 · Leaf area per plant	-0.66	0.62	0.66	0.62	19.4	1.9
R6 · Total dry matter per plant	-0.50	0.53	0.50	0.53	18.8	5.6
R6 · Branches per plant	-0.37	0.27	0.44	0.33	8.1	0.0
R6 · Main-stem nodes per plant	-0.88	0.69	0.88	0.69	21.9	3.8
R6 · Plant height	0.11	-0.10	0.20	0.18	10.6	0.6

Figure S3. Directional cross-season shift of compact predictors. Aligned heatmaps report the 20 compact trait–stage variables for 2021/22 → 2022/23 and 2022/23 → 2021/22. Panel **A** shows the signed standardised mean difference, panel **B** shows the first Wasserstein distance divided by the development-season standard deviation, and panel **C** shows the percentage of independent-season observations outside the development-season range. Each directional estimate is based on 160 observations per season. The colour scales encode descriptive distributional-shift measures; the corresponding independent-to-development variance ratios are reported in Table S3.

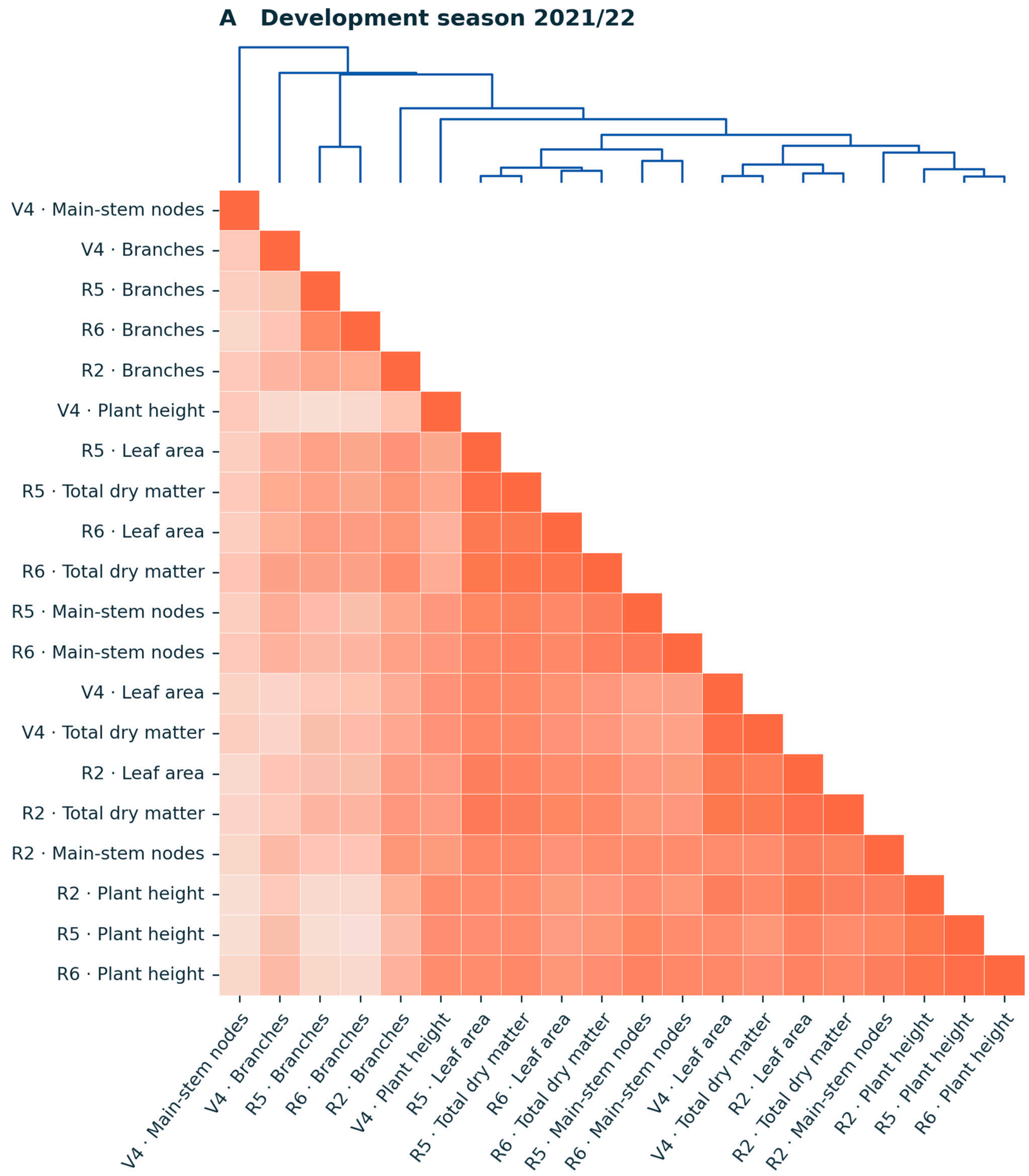
A Development season 2021/22
V4 · Main-stem nodes
V4 · Branches
R5 · Branches
R6 · Branches
R2 · Branches
V4 · Plant height
R5 · Leaf area
R5 · Total dry matter
R6 · Leaf area
R6 · Total dry matter
R5 · Main-stem nodes
R6 · Main-stem nodes
V4 · Leaf area
V4 · Total dry matter
R2 · Leaf area
R2 · Total dry matter
R2 · Main-stem nodes
R2 · Plant height
R5 · Plant height
R6 · Plant height

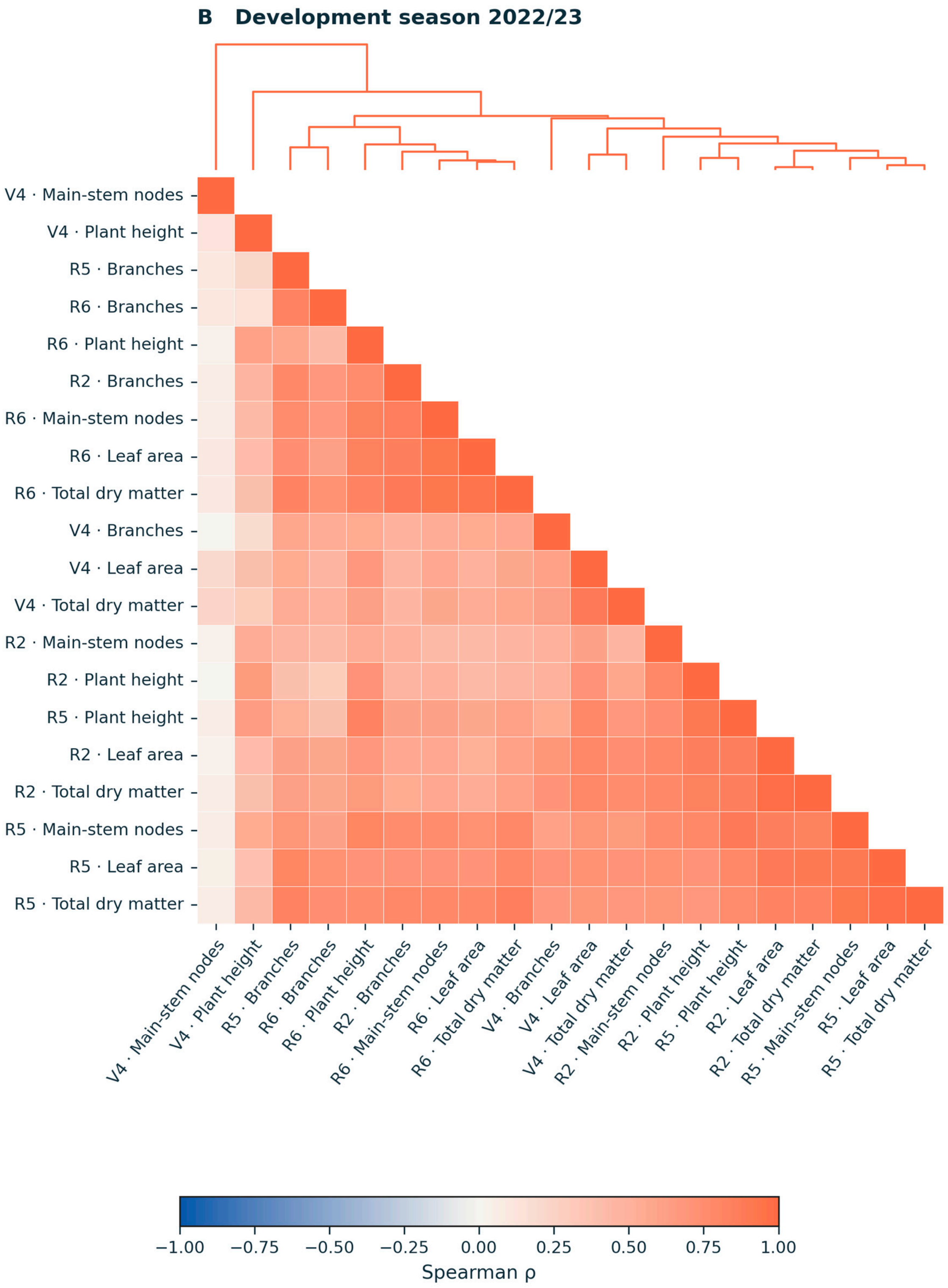

Figure S4. Development-season correlation structure of the compact predictors. Panels **A** and **B** show 2021/22 and 2022/23, respectively (160 subplot observations per season). Triangular matrices display Spearman's ϱ; variables are ordered separately within each season by average-linkage clustering of 1 − |ϱ|. Dendrograms show the corresponding hierarchy. Grain yield was not used in the ordering.

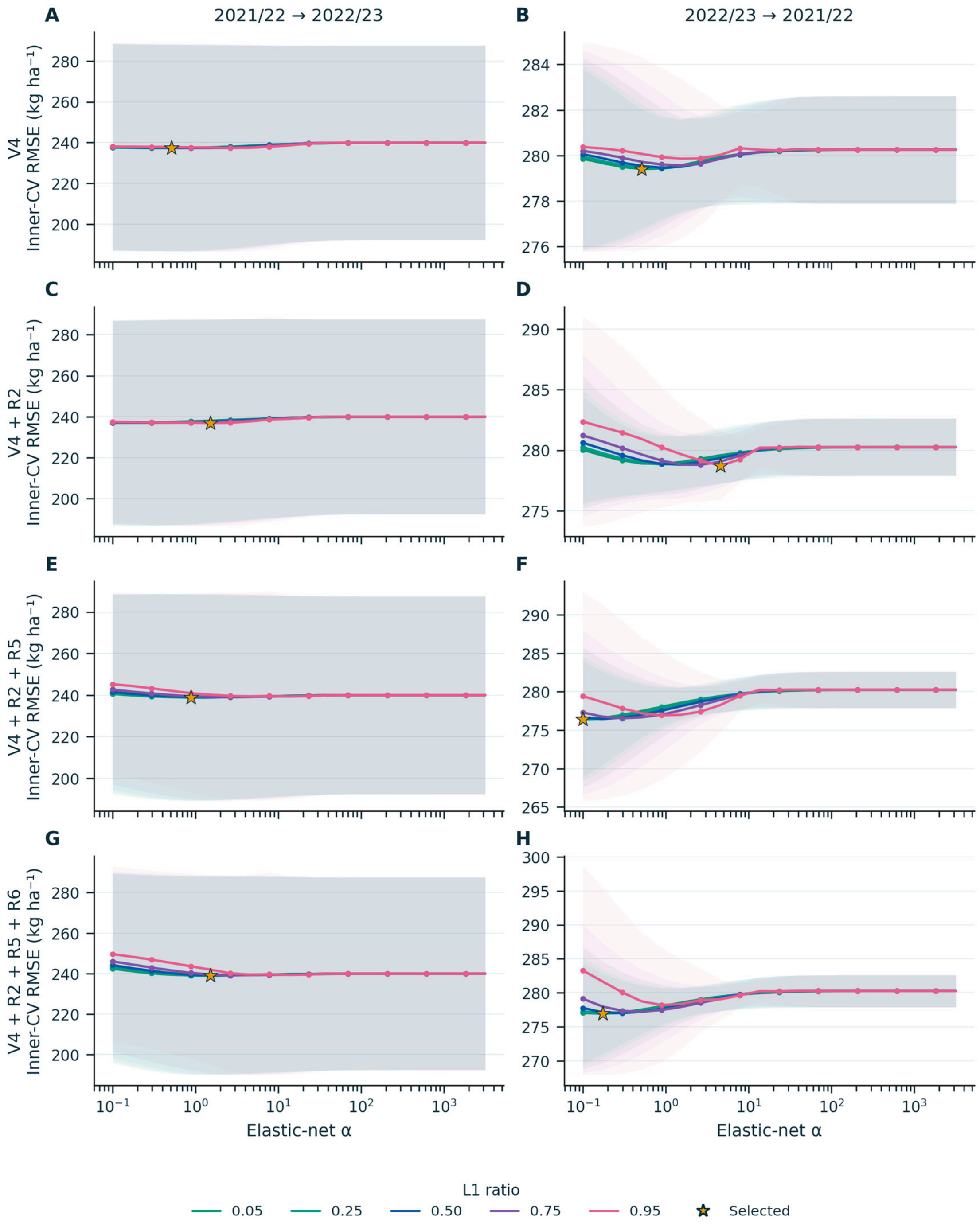

Figure S5. Grouped inner cross-validation for compact elastic-net models. The mean inner-validation RMSE is shown across elastic-net penalty strengths for each L1 proportion and information gate. Curves are means across the four leave-one-block-out folds, ribbons show ±1 standard deviation across folds, and symbols identify the selected combinations. Panels **A–B** show V4, panels **C–D** V4 + R2, panels **E–F** V4 + R2 + R5 and panels **G–H** V4 + R2 + R5 + R6. Within each pair, the left panel uses 2021/22 for development and 2022/23 for independent assessment, whereas the right panel uses the reciprocal direction. All preprocessing quantities and management offsets were re-estimated within each inner development fold.

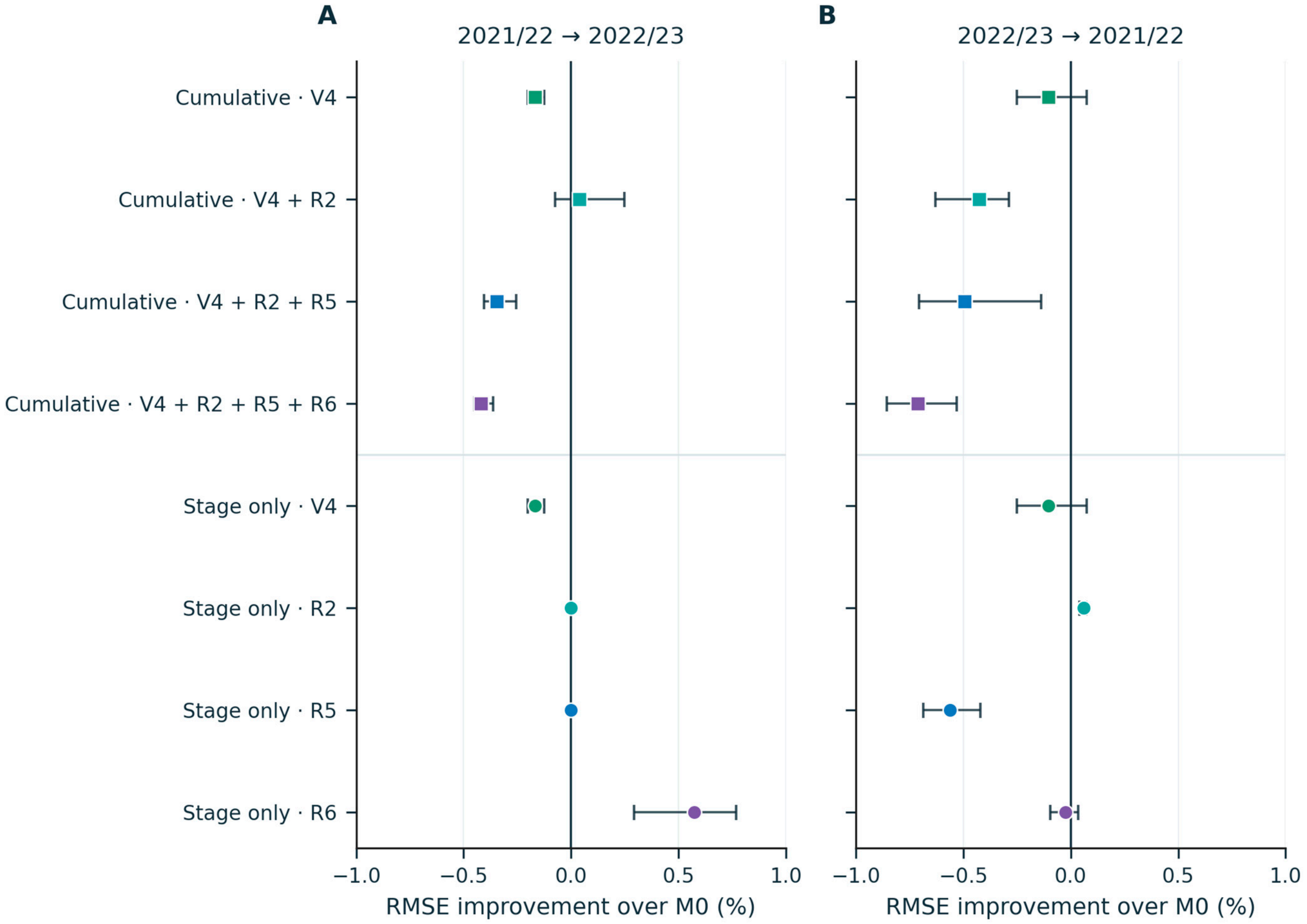

Figure S6. Stage-only and cumulative phenotype representations. Panels **A** and **B** show the 2021/22 → 2022/23 and 2022/23 → 2021/22 transfer directions, respectively. Squares denote cumulative models, and circles denote stage-only models. The cumulative labels are V4, V4 + R2, V4 + R2 + R5 and V4 + R2 + R5 + R6; the stage-only labels are V4, R2, R5 and R6. Points show paired RMSE gains over matched M0 in both transfer directions. Horizontal bars are primary conditional within-season whole-block bootstrap 95% intervals from 2,000 paired resamples; lighter bars show the sowing-period-stratified robustness intervals. Positive values favour phenotype augmentation, and the vertical line denotes no change.

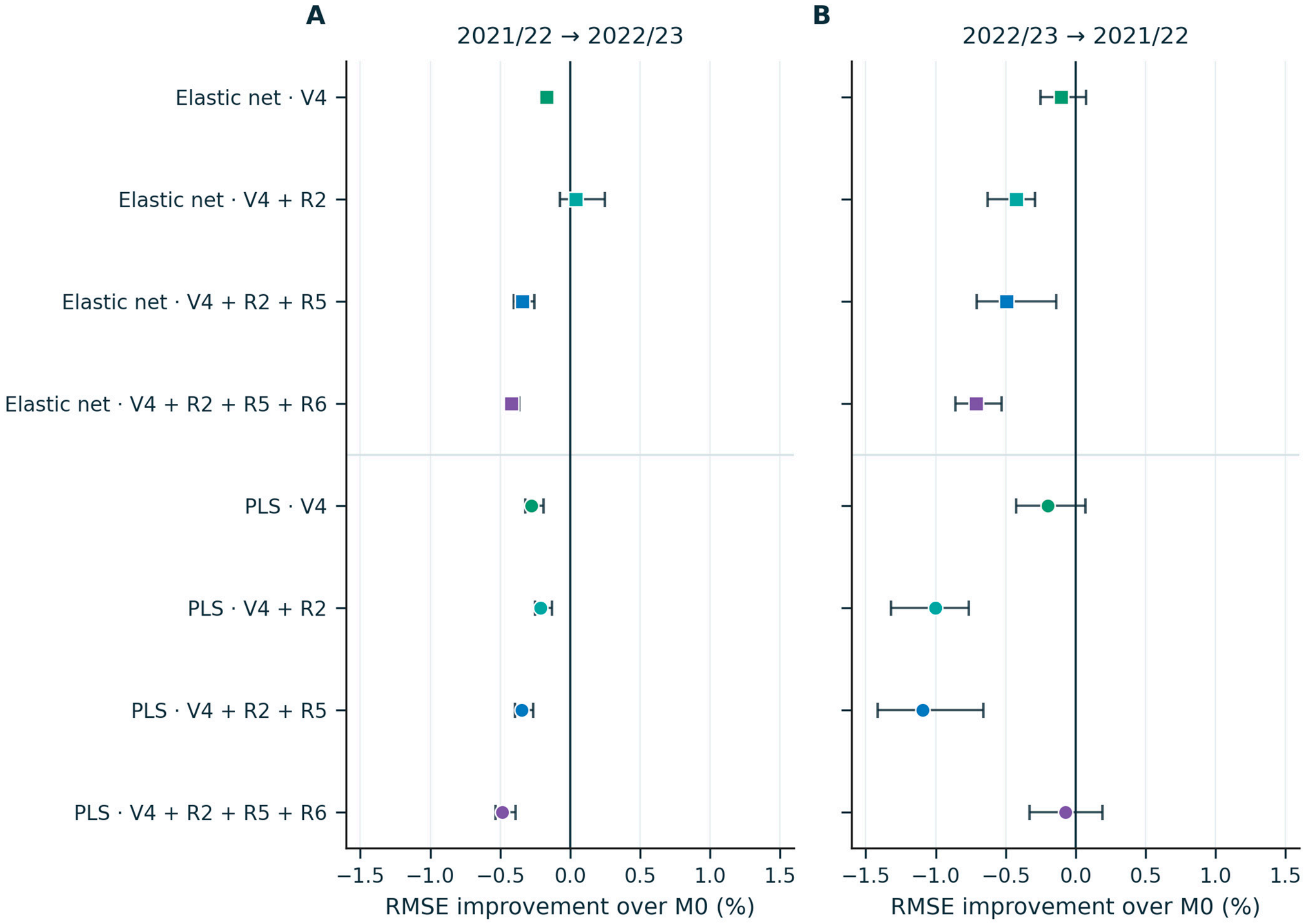

Figure S7. Elastic-net and partial least squares models. Panels **A** and **B** show the 2021/22 → 2022/23 and 2022/23 → 2021/22 transfer directions, respectively. The paired RMSE gain over matched M0 is shown for the compact cumulative elastic-net and partial least-squares models in both transfer directions. Hyperparameters were selected exclusively by grouped cross-validation during the development season. Horizontal bars are primary conditional within-season whole-block bootstrap 95% intervals from 2,000 paired resamples, and lighter bars show the sowing-period-stratified robustness intervals. Each algorithm–gate combination is compared with M0 on identical observations.

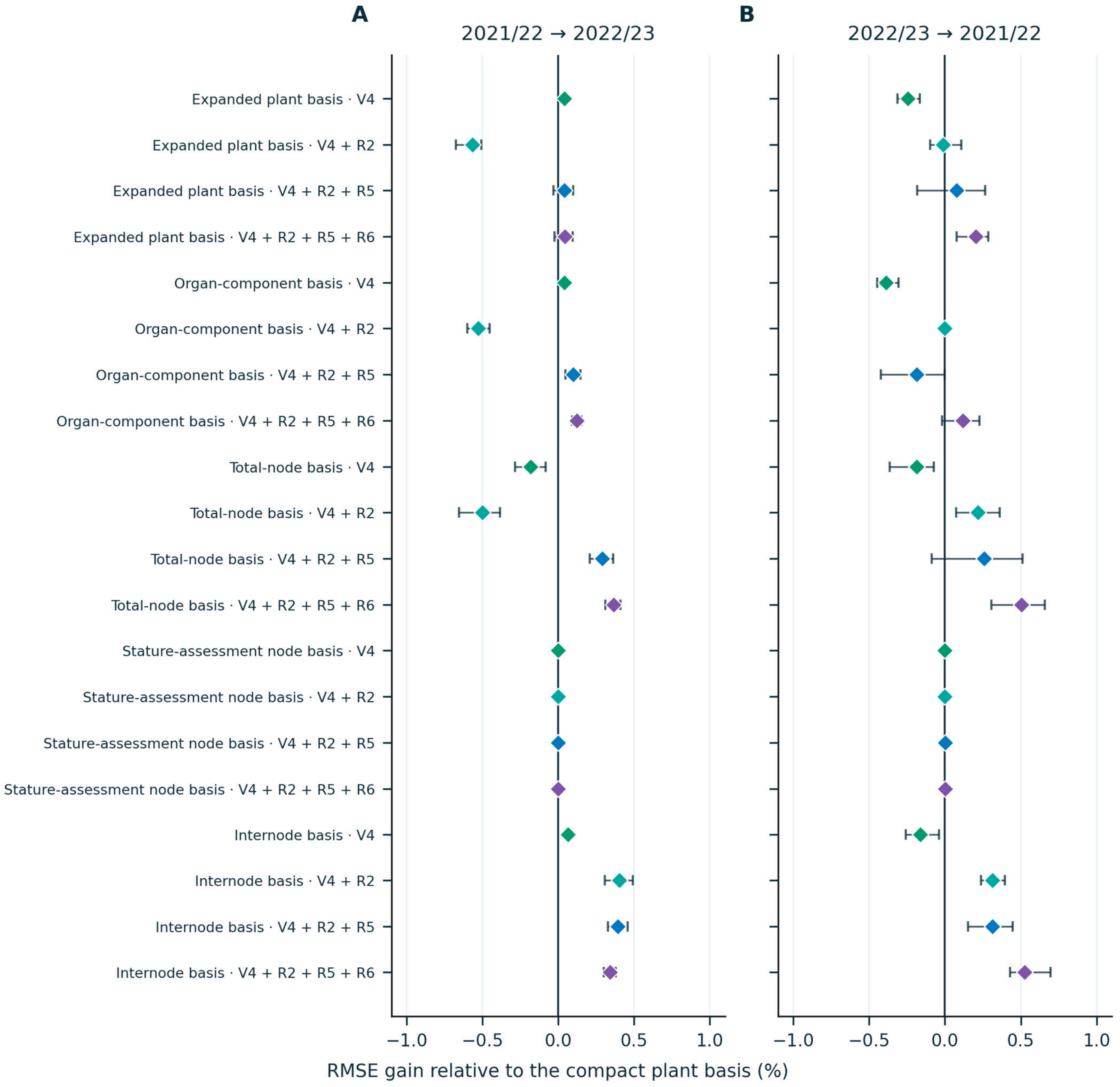

Figure S8. Direct contrasts among plant-scale predictor representations. Panels **A** and **B** show the 2021/22 → 2022/23 and 2022/23 → 2021/22 transfer directions, respectively. In each direction, the expanded plant, organ component, total node, stature assessment node and internode representations are contrasted directly with the compact plant representation at the same cumulative information gate. Positive RMSE gains favour the alternative representation. Points are paired estimates, and horizontal bars are conditional within-season whole-block bootstrap 95% intervals from 2,000 resamples. All comparisons use identical independent-season observations; management-comparator robustness is reported in Figure 9 and Table S9.

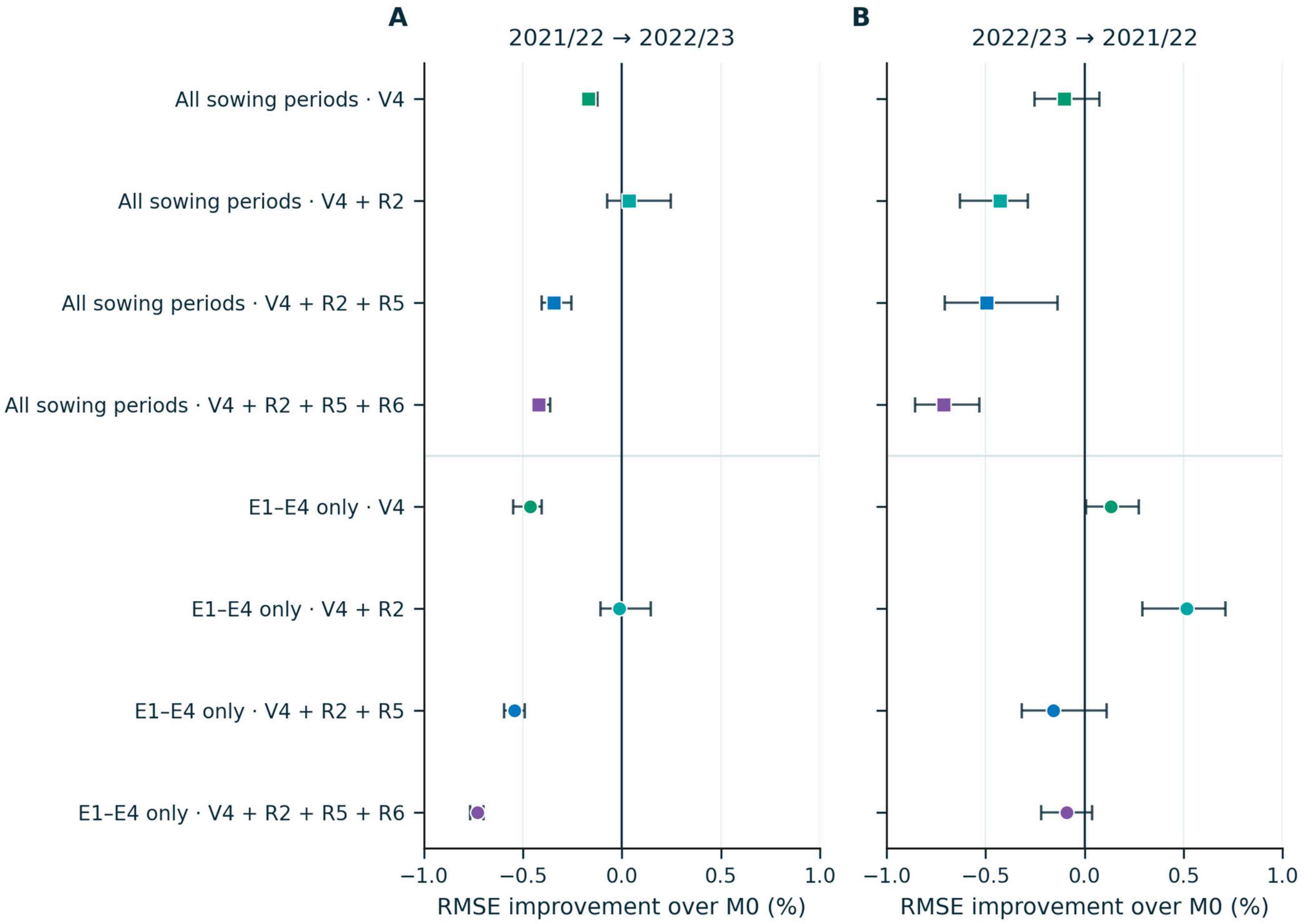

Figure S9. Sensitivity across the E1–E5 and E1–E4 sowing domains. Panels **A** and **B** show the 2021/22 → 2022/23 and 2022/23 → 2021/22 transfer directions, respectively. The paired RMSE gain over matched M0 is shown for the compact cumulative elastic-net model on the original yield scale evaluated within the complete E1–E5 sowing domain and the E1–E4 domain. The E1–E5 estimates use n = 160 independent-season plots, and the E1–E4 estimates use n = 128. Each phenotype model is compared with an M0 estimated for the corresponding sowing-domain population. The horizontal bars are 95% intervals from 2,000 paired resamples under the resampling structure appropriate for the displayed domain.

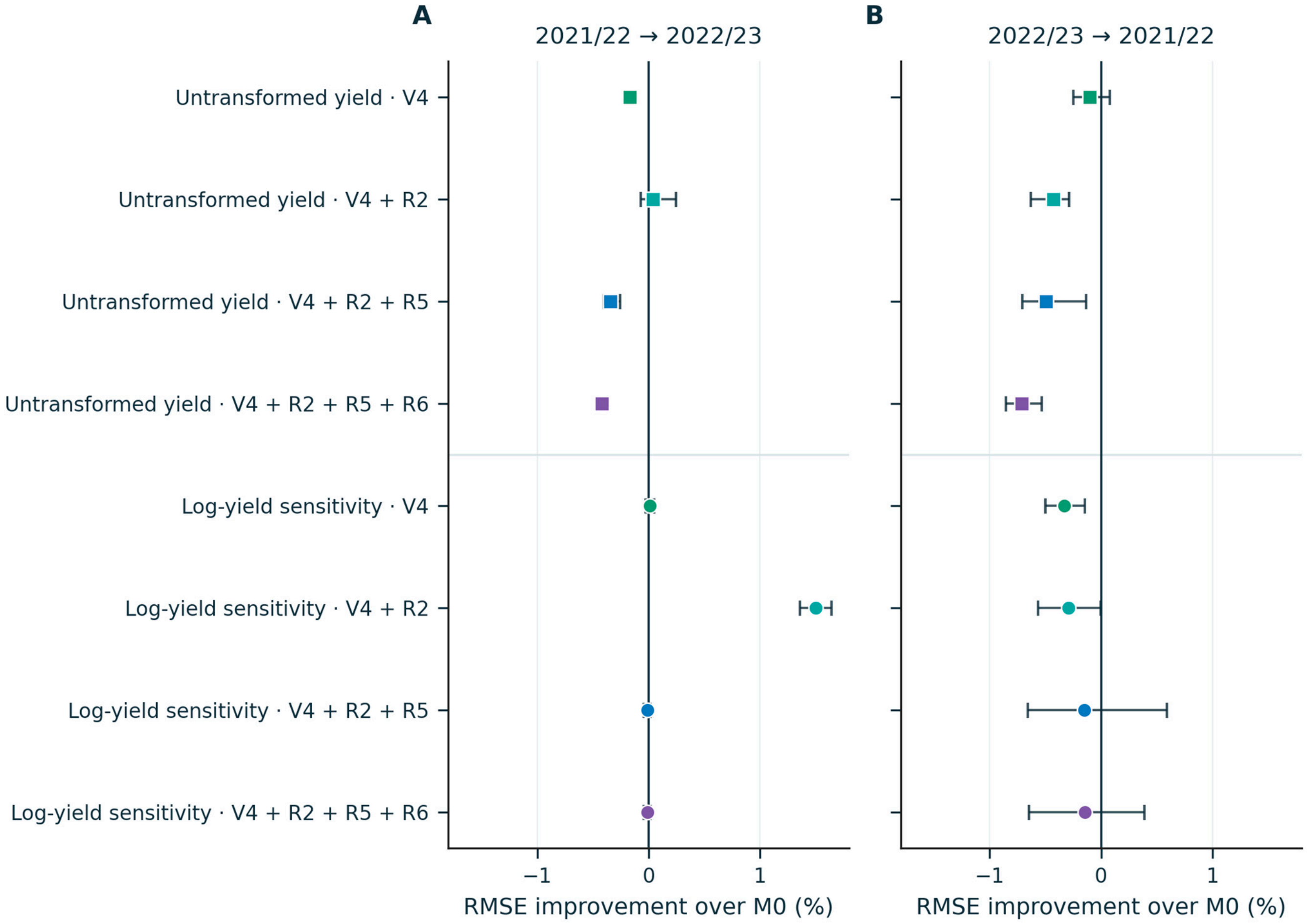

Figure S10. Untransformed and log-transformed yield targets. Panels **A** and **B** show the 2021/22 → 2022/23 and 2022/23 → 2021/22 transfer directions, respectively. Paired RMSE gain over M0 on the original yield scale is shown for compact cumulative elastic-net models fitted to untransformed or log-transformed yield. Log predictions were returned to kg ha^{-1} with a Duan smearing factor estimated exclusively from the development season, after which all errors and contrasts were evaluated on the original yield scale. Horizontal bars are primary conditional within-season whole-block bootstrap 95% intervals from 2,000 paired resamples, with the sowing-period-stratified intervals shown as a robustness layer.

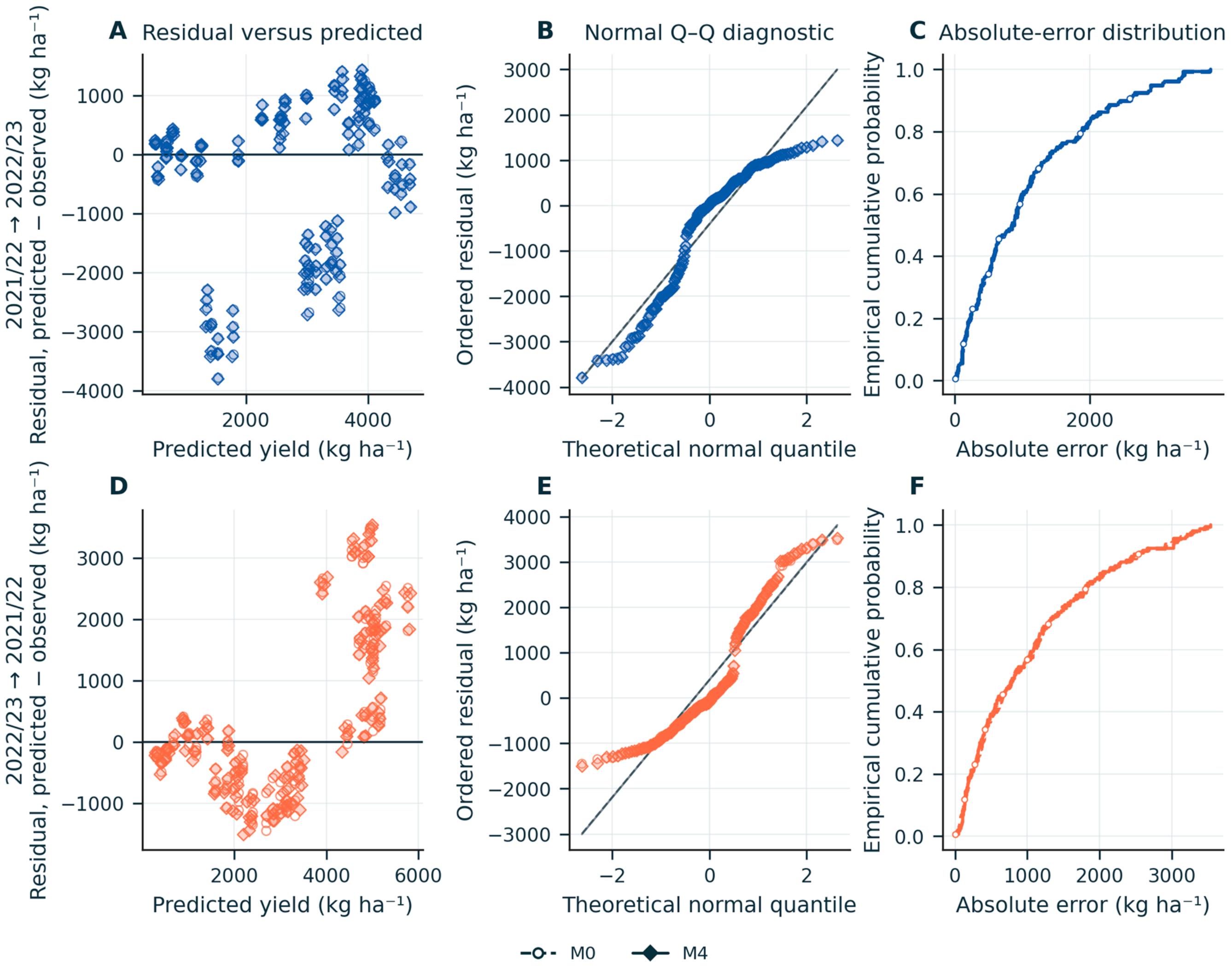

Figure S11. Residual diagnostics for management and terminal phenotype gates. **(A–F)** Rows represent the two transfer directions; columns show residual-versus-predicted scatterplots, normal quantile–quantile diagnostics and empirical cumulative distributions of absolute error for M0 and M4. Each model–direction combination contains 160 independent-season plot observations. These diagnostic methods characterise heteroscedasticity, tail behaviour and directional error concentration across reciprocal temporal predictions.
